# Impact of physical activity programs and services for older adults: a rapid review

**DOI:** 10.1186/s12966-022-01318-9

**Published:** 2022-07-14

**Authors:** Marina B. Pinheiro, Juliana S. Oliveira, Jennifer N. Baldwin, Leanne Hassett, Nathalia Costa, Heidi Gilchrist, Belinda Wang, Wing Kwok, Bruna S. Albuquerque, Luiza R. Pivotto, Ana Paula M. C. Carvalho-Silva, Sweekriti Sharma, Steven Gilbert, Adrian Bauman, Fiona C. Bull, Juana Willumsen, Catherine Sherrington, Anne Tiedemann

**Affiliations:** 1 Institute for Musculoskeletal Health, The University of Sydney, Sydney Local Health District, Sydney, Australia; 2 School of Public Health, Faculty of Medicine and Health, The University of Sydney, Sydney, Australia; 3 School of Health Sciences, Faculty of Medicine and Health, The University of Sydney, Sydney, Australia; 4 WHO Collaborating Centre for Physical Activity, Nutrition and Obesity, Charles Perkins Centre, The University of Sydney, Sydney, Australia; 5 Physical Activity Unit, Department of Health Promotion, Division of Universal Health Coverage and Healthier Populations, World Health Organization (WHO), Geneva, Switzerland

**Keywords:** Physical activity, Older adults, Aged, Exercise, Healthy ageing

## Abstract

**Background:**

Knowledge of which physical activity programs are most effective for older adults in different sub-populations and contexts is limited. The objectives of this rapid review were to: 1) Overview evidence evaluating physical activity programs/services for older adults; and 2) Describe impact on physical activity, falls, intrinsic capacity (physical domain), functional ability (physical, social, and cognitive/emotional domains), and quality of life.

**Methods:**

We conducted a rapid review of primary studies from 350 systematic reviews identified in a previous scoping review (March 2021: PEDro, MEDLINE, CINAHL, Cochrane Database). For Objective 1, we included intervention studies investigating physical activity programs/services in adults ≥ 60 years. Of these, we included good quality (≥ 6/10 PEDro scale) randomised controlled trials (RCTs) with ≥ 50 participants per group in Objective 2.

**Results:**

Objective 1: Of the 1421 intervention studies identified from 8267 records, 79% were RCTs, 87% were in high income countries and 39% were good quality. Objective 2: We identified 87 large, good quality RCTs (26,861 participants). Overall activity promotion, structured exercise and recreation/sport had positive impacts (≥ 50% between-group comparisons positive) across all outcome domains. For overall activity promotion (21 intervention groups), greatest impacts were on physical activity (100% positive) and social outcomes (83% positive). Structured exercise (61 intervention groups) had particularly strong impacts on falls (91% positive), intrinsic capacity (67% positive) and physical functioning (77% positive). Recreation/sport (24 intervention groups) had particularly strong impacts on cognitive/emotional functioning (88% positive). Multicomponent exercise (39 intervention groups) had strong impacts across all outcomes, particularly physical activity (95% positive), falls (90% positive) and physical functioning (81% positive). Results for different populations and settings are presented.

**Conclusion:**

Evidence supporting physical activity for older adults is positive. We outline which activity types are most effective in different populations and settings.

**Supplementary Information:**

The online version contains supplementary material available at 10.1186/s12966-022-01318-9.

## Introduction

Globally there are more than 1 billion adults aged over 60 years and this number is forecast to increase to 2 billion by 2050 [1]. The 2020 World Health Organization (WHO) *Guidelines on Physical Activity and Sedentary Behaviour *[2] recommend that older adults should engage in regular aerobic activity, muscle strengthening activities as well as multicomponent physical activity that incorporates strength and balance training to improve physical function and prevent falls [2]. However older adults have higher rates of physical inactivity, with 19–25% of adults aged 60–69 years, and 42–59% of adults aged 80 years and older, not meeting physical activity guidelines for aerobic activity [3] 

The WHO Global Action Plan on Physical Activity (GAPPA) was launched in 2018 [4]. GAPPA set out four strategic policy objectives (Active Societies, Active Environments, Active People and Active Systems) and 20 recommended policy actions that are applicable to all countries and that aim to address the social, cultural, environmental and individual determinants of physical inactivity. To assist countries to adopt, tailor and implement the 20 policy recommendations of GAPPA into local contexts, WHO is developing ACTIVE [5], a technical toolkit with implementation guidance for key approaches, settings and populations.

To inform the development of ACTIVE, our group undertook a scoping review of systematic reviews investigating physical activity interventions and programs for older adults [6]. This scoping review mapped the existing literature and identified gaps in the evidence. We identified 39 systematic reviews for interventions aimed at increasing overall physical activity in older adults, and 342 reviews investigating specific physical activity programs and services (GAPPA Objective 3, Active People). However, sport and workplace interventions, and physical activity in diverse populations, were under-investigated.

As most of the reviews identified in our scoping review focused on a mixture of settings, populations and interventions [6], it was not possible to make specific recommendations about programs and services to implement for different populations and in different contexts. We concluded that a review of primary studies would be needed to develop more specific recommendations. Therefore, the aim of the present review was to inform the WHO and others on the effectiveness of physical activity programs and services for older adults. Specific objectives included:

Objective 1: To describe the extent and quality of evidence evaluating the effectiveness of physical activity programs and services for older adults.

Objective 2: To describe the impact of different physical activity programs (physical activity promotion, structured exercise, recreation/sport) compared to no intervention, on outcomes of physical activity, falls, intrinsic capacity (physical domain), functional ability (physical, social, cognitive and emotional domains), wellbeing and quality of life, and also to describe the impact of interventions undertaken in different populations, locations and settings.

## Methods

We conducted a rapid review and followed the Preferred Reporting Items for Systematic Reviews and Meta-Analyses *(PRISMA)* [7]*.* A protocol was prepared in advance and published on the Open Science Framework website [8].

### Data sources

Our previous scoping review identified 39 systematic reviews of physical activity interventions and 342 systematic reviews of physical activity programs/services for older adults [6]. Of the 39 reviews of interventions, 31 reviews were also included again as programs/services, resulting in 350 unique reviews. Therefore, for the current rapid review, we screened primary studies included in these 350 systematic reviews. The search for the previous review was conducted from 1 January 2010 to 1 November 2020 on PEDro, MEDLINE, CINAHL, and the Cochrane Database of Systematic Reviews (Appendix 1). For the present review, we updated this search on 18 March 2021 using the same strategy and databases. This identified 25 additional reviews. We also screened four reviews identified via hand searching. The flow chart showing selection of reviews from the updated search is provided in Appendix 2 and a description of the reviews in Appendix 3.

Our previous review [6] identified limited studies investigating sports in older adults. Since the previous search strategy did not include keywords related to “sports”, we developed a new search strategy that included specific keywords related to sports and searched MEDLINE, CINAHL, SPORTDiscus, PEDro. No date limit was applied, and searches were performed on 19 and 20 April 2021. The search strategy and the flow chart describing the selection of sports primary studies is provided in Appendix 4.

### Study selection and data extraction

A pool of eight reviewers assessed the eligibility of primary studies based on title and abstract, followed by full text where indicated. All reviewers received training on the eligibility criteria and attended regular meetings to discuss questions regarding the criteria. One reviewer assessed the eligibility of all primary studies, and a second reviewer checked the eligibility assessment of a randomly selected sample of studies (5%). Any disagreements were discussed and resolved.

Screening and data extraction were divided into two objectives:

Objective 1. Primary studies identified in the reviews and searches were screened according to the following eligibility criteria (additional details on eligibility criteria are available in Appendix 5):

i) Population: adults aged 60 years and older, or studies in which the mean age of participants was ≥ 60 years if age was not specified as an inclusion criterion. We included studies in which all participants had a physical impairment (e.g., mobility limitation) or particular symptoms (e.g., low mood or mild cognitive impairment). We excluded studies in which all participants had experienced a particular health event (e.g., stroke) or had a formal diagnosis (e.g., major mental illness or dementia).
ii) Intervention: physical activity program or service.
iii) Comparator: any comparator.
iv) Outcome: physical activity, falls, intrinsic capacity (physical domain), functional ability (physical, social, and cognitive and emotional domains), and wellbeing and quality of life; and
v) Study design: interventional studies.

The following information was extracted for all studies included in Objective 1: study design, sample size, country, type of physical activity, comparator, methodological quality for randomised trials using the Physiotherapy Evidence Database (PEDro) scale score [9]. One reviewer from the pool of eight extracted data for studies included in Objective 1 and a second reviewer checked 5% of studies.

Objective 2: Studies identified in Objective 1 were included in Objective 2 analyses if they met the following additional criteria:

i) Comparison: no active intervention.
ii) Study design: randomised controlled trial (RCT).
iii) Sample size: at least 50 participants per group on average.
iv) Methodological quality: good quality, as determined by a score of at least 6 on the PEDro scale (0 to 10 scale).
v) Results of between-group statistical comparisons reported for relevant outcomes.

Studies were excluded if they did not include a non-active intervention comparison, if the study design was not an RCT, if there were fewer than 50 participants per group, if the methodological quality was ≤ 5 out of 10 on the PEDro scale, and if no between-group comparisons were reported for relevant outcomes.

Where multiple publications were from the same trial, we only included those that reported different outcomes. Duplicate publications reporting the same participants and outcomes were excluded.

The sample size of 50 per group was chosen as small studies tend to have greater effects that are not replicated in larger samples [10]. We were also more interested in programs that had been delivered to larger numbers of people as these programs were likely more suitable for scaling up.

The PEDro scale was developed to assess the methodological quality of trials indexed by the Physiotherapy Evidence Database. A score of 6/10 or higher is considered to reflect “good” methodological quality although the validity of these cut-off scores has not been evaluated [11]. Two items are for blinding of those receiving and delivering interventions so the maximum score for trials evaluating physical activity interventions is 8/10. PEDro scores were downloaded from the PEDro database (https://www.pedro.org.au/).

We extracted detailed information (Population, Intervention, Comparison, Outcomes) for studies included in Objective 2 using a modified version of the framework our team developed for our previous review. [6]. The framework covers detailed information to describe the study population or sample, characteristics of the intervention and comparison, and outcomes used to evaluate the intervention. Development of the structure and content of the framework was informed by the WHO *Global Action Plan on Physical Activity* (GAPPA) [4], the Consolidated Standards of Reporting Trials (CONSORT) Template for Intervention Description and Replication (TIDieR) framework [12], and the Prevention of Falls Network Europe (ProFaNE) taxonomy [13]. The classification of outcomes was guided by the *World report on ageing and health* [14]*,* the *Decade of healthy ageing: baseline report *[15] and the *International classification of functioning, disability and health (ICF)* [16]*.* The framework was extensively pilot tested during our previous scoping review of physical activity interventions [6].

The same pool of eight reviewers undertook data extraction for Objective 2. One reviewer performed initial data extraction for included studies and a second reviewer checked the data for most (> 80%) studies.

All relevant outcomes reported by the primary studies were classified as per our framework [6]. Briefly, outcome domains included physical activity, falls, intrinsic capacity (physical domain), functional ability (physical, social, cognitive and emotional domains), and wellbeing and quality of life. Intrinsic capacity refers to the composite of all the mental and physical capacities of an individual, while functional ability comprises the health-related attributes that enable people to be and to do what they have reason to value, encompassing both intrinsic capacity and environmental characteristics*. *[14]. Further information regarding these resources is provided in Appendix 6.

Intervention effect for each relevant outcome was extracted and categorised as follows: positive significant, positive non-significant, no/negligible effect, negative non-significant, negative significant. In choosing this approach, we were guided by the Cochrane Collaboration Handbook for systematic reviews chapter describing methods for synthesising data from trials without undertaking meta-analysis [17].

### Data synthesis

We summarised descriptive information for all included studies in figures and tables. We also summarised the studies’ effects based on recommendations for synthesising and presenting findings of reviews when meta-analysis is not possible or appropriate [18]. For each study we calculated the proportion of effects that were positive as well as positive and statistically significant. We also calculated these proportions across all studies by outcomes and by outcome domains. These results are summarised in tables and plotted in figures.

## Results

**Fig. 1:**
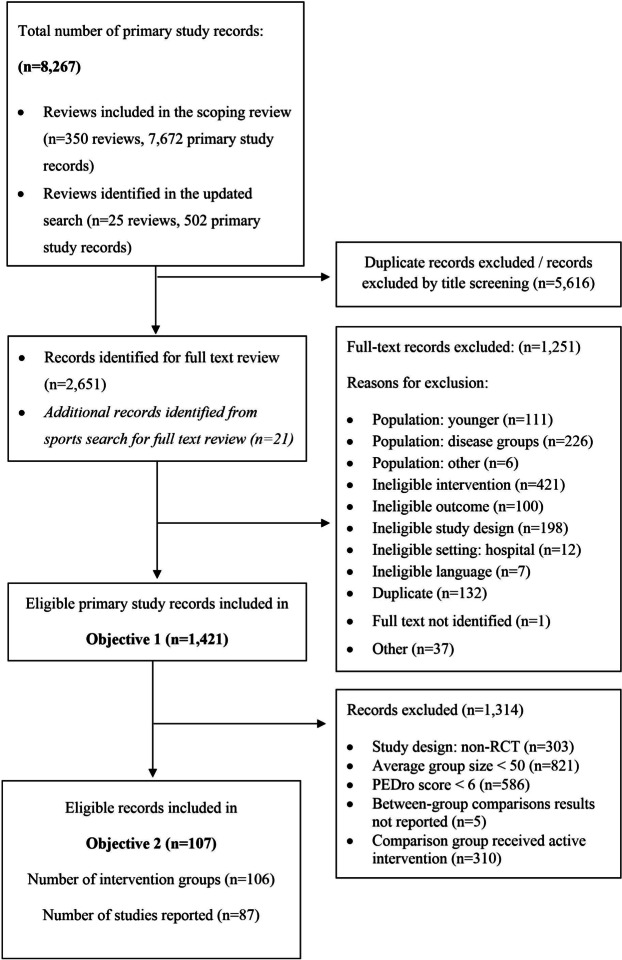
Flow chart for selection of primary studies investigating physical activity programs for older adults

We identified and screened 8267 primary study records from the systematic reviews included in our previous scoping review and in our hand search. Of these, 1421 records were included in Objective 1 and 107 records reporting 87 unique trials were included in Objective 2. The flow chart for selection of primary studies for Objectives 1 and 2 is shown in Fig. 1.

### Objective 1: Studies investigating physical activity programs/services in older adults

Most records (79%) identified in Objective 1 were RCTs investigating the effectiveness of physical activity interventions compared with no intervention (57%) (Table 1). The most common type of physical activity investigated was structured exercise (79%), followed by promotion of overall physical activity (9%). Overall, the records had a relatively small sample size (median = 55 participants). Most RCTs (61%) were of poor/fair methodological quality (median PEDro score 5, 0 to 10 scale).

**Table 1:** Characteristics of the 1421 primary studies of physical activity interventions for older adults (Objective 1)

Characteristics	N (%)
*Country* [n (%)]^*^	
High income	1240 (87)
Upper-middle income	171(12)
Lower-middle income	6 (0.4)
Low income	0 (0)
Mixed	1 (0.1)
Not specified	3 (0.2)
*Sample size* [mean (SD), median, range]	126 (295), 55, 6–5893
*Study design*	
RCT [n (%)]	1124 (79)
Non-RCT [n (%)]	297 (21)
*Type of physical activity* [n (%)]	
Promoting overall activity	127 (9)
Structured exercise	1,120 (79)
Recreation	158 (11)
Sport	16 (1)
*Comparison* [n (%)]^**^	
No intervention	812 (57)
Higher dose of same activity	21 (2)
Behaviour change support to PA	8 (1)
Different delivery mode of same activity	39 (3)
Different physical activity	199 (14)
Nutrition	6 (0.4)
Education	86 (6)
Combination of interventions	21(2)
Other	229 (16)
PEDro score /10^***^	
Poor 0–3 [n (%)]	73 (7)
Fair 4–5 [n (%)]	601 (54)
Good 6–8 [n (%)]	438 (39)
Median score^****^ [(SD), range]	5 (1.3), 1–8

*RCT* randomised controlled trial, *SD* standard deviation

^*^Income classification strata as per World Bank Country and Lending Groups 2021

^**^Percentages total > 100 as some studies included more than one intervention group

^***^PEDro scores are available only for RCTs (*n* = 1124), PEDro score not available for *n* = 12 trials. The optimal score for trials evaluating physical activity interventions is 8/10

^****^The median and average PEDro score was the same among studies that met the inclusion criteria for Objective 1

The included records investigated a total of 178,622 participants, although the actual number of participants included in the studies is smaller as there are multiple publications from the same study, however we did not calculate the actual total number of participants included across individual studies in Objective 1. Most studies were conducted in high income countries (87%). None of the included studies were conducted in low-income countries and only six in lower-middle income countries.

### Objective 2: Large, good quality trials investigating physical activity programs/services in older adults compared with no intervention

#### Description of included studies

We extracted data from 107 records reporting 87 large, good quality (≥ 6/10 PEDro scale), RCTs investigating physical activity programs and services for older adults. A total of 106 intervention groups were included as some studies had two intervention groups compared to control. Appendix 7 outlines the Population, Intervention and Outcome data for these 87 trials, classified according to our framework.

#### Setting

All studies eligible for inclusion in Objective 2 were conducted in high- and upper-middle income countries. No studies were undertaken entirely in vulnerable/underserved populations according to cultural, linguistic, migrant, indigenous or socio-economic status. One study was conducted in a combination of urban and rural/remote areas, with most studies not specifying remoteness (*n* = 73 studies) or conducted in urban areas (*n* = 13 studies).

#### Participants

Most studies recruited both males and females (*n* = 76 studies). Most participants were aged ≥ 60 years (*n* = 24 studies) or ≥ 65 years (*n* = 32 studies), with a smaller number investigating older subgroups such as ≥ 80 years (*n* = 3 studies) or ≥ 85 years (*n* = 1 study). Some studies were entirely undertaken in participants with physical impairments/limitations (mobility: *n* = 11 studies, frailty: *n* = 6, falls risk: *n* = 11), mild cognitive impairment (*n* = 9), symptoms of depression (*n* = 1) and mixed chronic conditions (*n* = 3). We did not include studies entirely undertaken in adults with a particular health condition as per the inclusion criteria. The total number of participants across studies included in Objective 2 was 26,861 (mean 309, standard deviation 259, median 207, range 100 to 1,635).

#### Type of physical activity

Of the 106 intervention groups included in the 87 trials, overall activity promotion was investigated in 21 (20%) intervention groups [19–38]. Structured exercise was investigated in 61 (57%) intervention groups, including strength/resistance/power exercise (14 intervention groups) [25, 39–50], balance/functional/neuromotor exercise (4 intervention groups) [43, 51–53], walking (1 intervention group) [54], endurance exercise (2 intervention groups) [55, 56], and multicomponent exercise (39 intervention groups) [40, 42, 43, 49, 57–99]. Recreation/sport was investigated in 24 (25%) intervention groups, including Tai chi (15 intervention groups),[48, 50, 78, 100–115], yoga and Pilates (3 intervention groups) [116–118], dance (5 intervention groups)[119–124] and competitive sport (1 intervention group) [125].

#### Location

A description of included studies categorised according to location is provided in Appendix 8. Thirty-eight studies were conducted in community facilities, 22 were conducted at home, 3 in outpatient health facilities, 8 in residential aged care facilities, 4 in retirement villages and 14 in no set location.

#### Outcomes investigated

**Fig. 2:**
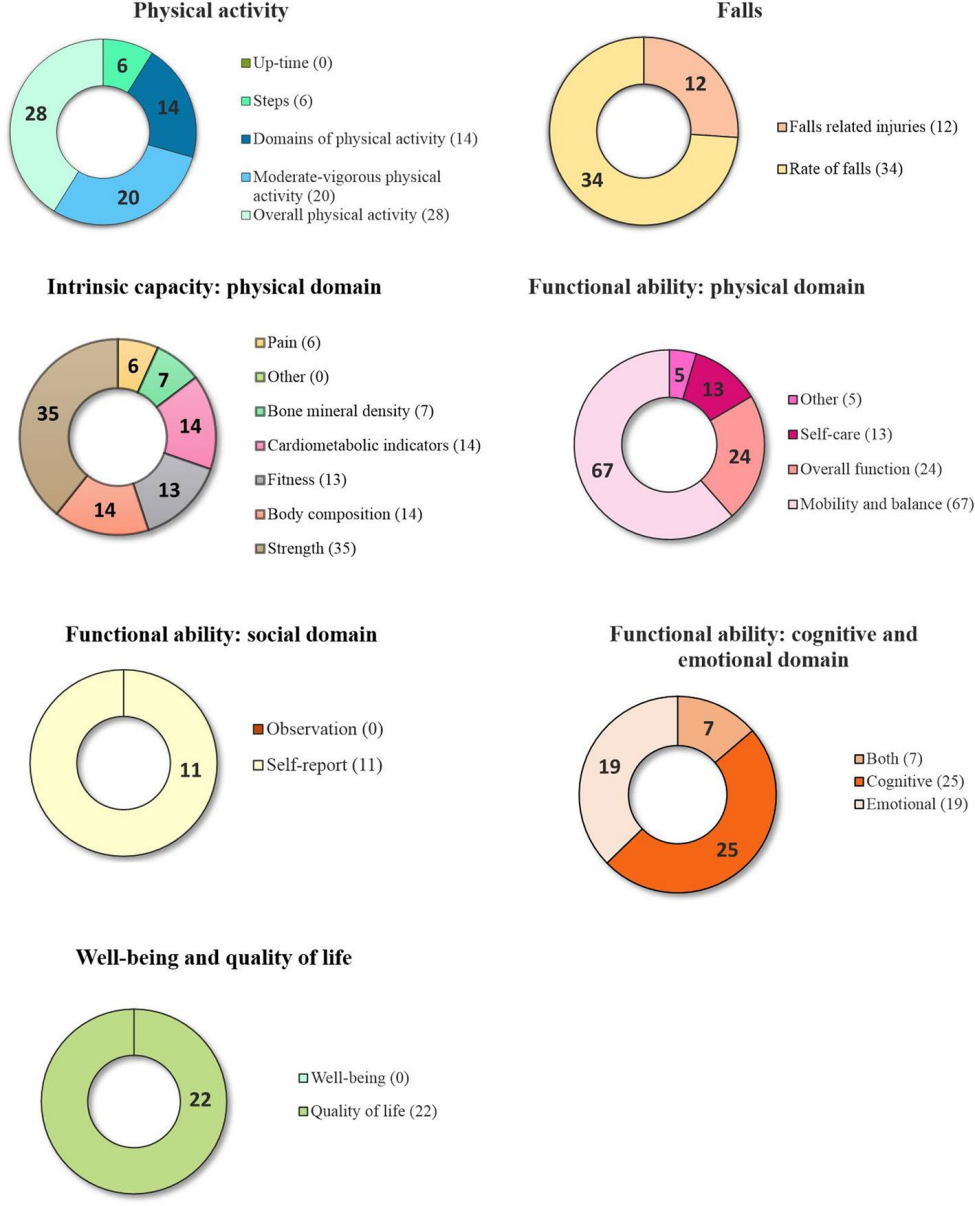
Number of intervention groups investigating different outcomes in Objective 2 (*n* = 87 studies)

The number of intervention groups investigating each outcome domain is shown in Fig. 2. Outcomes most commonly collected included overall physical activity (*n* = 28 intervention groups), moderate to vigorous physical activity (*n* = 20), rate of falls (*n* = 34), strength (*n* = 35), mobility and balance (*n* = 67), cognitive and emotional functioning (*n* = 25 and 27 respectively, *n* = 7 collected both) and quality of life (*n* = 22).

#### Physical activity in all populations and locations

**Fig. 3:**
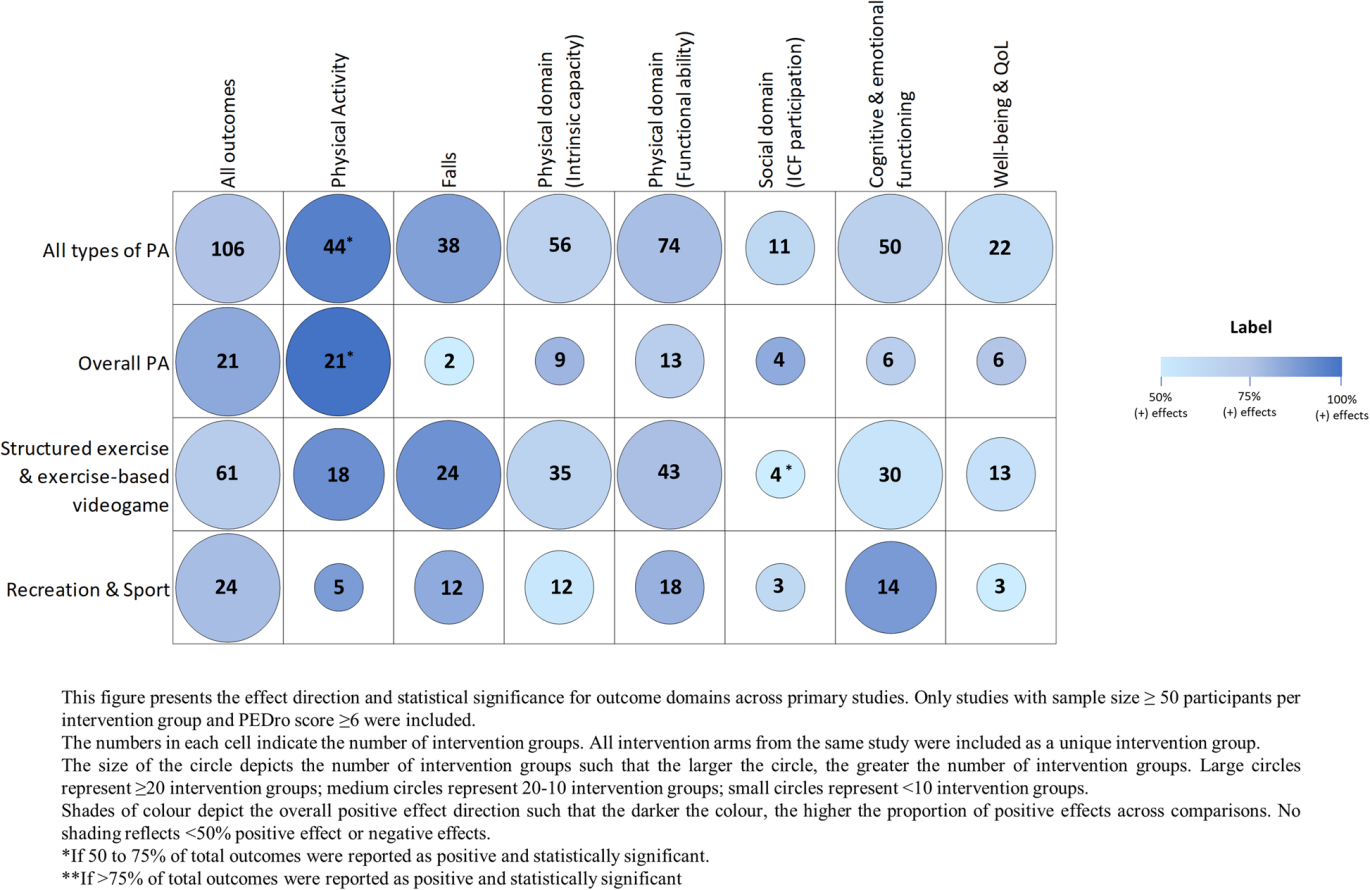
Physical activity interventions for older adults by type of activity: impact on different outcome domains

The results of the large, good quality RCTs were overall positive. Across all studies combined, positive effects (≥ 50% between-group comparisons positive) were evident for outcomes of physical activity (44 intervention groups, 125 individual outcomes), falls (38 intervention groups, 82 outcomes), intrinsic capacity: physical domain (56 intervention groups, 231 outcomes), functional ability: physical domain (74 intervention groups, 295 outcomes), social domain (11 intervention groups, 22 outcomes), cognitive and emotional domain (50 intervention groups, 190 outcomes), and quality of life (22 intervention groups, 31 outcomes) (Fig. 3, Appendix 9: Table A.9.1). For each individual physical activity type (overall activity promotion, structured exercise, and recreation/sport), positive effects (≥ 50% outcomes positive) were seen for all outcome domains. For physical activity outcomes, evidence was particularly strong for overall activity promotion (21 intervention groups, 84% outcomes statistically significant). For outcomes of falls and the physical domains of intrinsic capacity and functional ability, the strongest evidence was for structured exercise (24 intervention groups [91% positive], 35 intervention groups [67% positive] and 43 intervention groups [77% positive], respectively). For cognitive and emotional outcomes, the strongest impacts were seen for recreation and sport (14 groups, 88% positive) and structured exercise (30 intervention groups, 56% positive). Overall activity promotion and structured exercise had the strongest impacts on quality of life (6 intervention groups [75% positive] and 13 intervention groups [58% positive], respectively).

#### Physical activity in adults with physical impairments/limitations

In adults with physical impairments/limitations (32 intervention groups, 349 outcomes in total) there were positive effects of structured exercise on most outcomes except social domain (Appendix 10: Figure A.10, Table A.10). Strong impacts were seen for interventions promoting overall physical activity on outcomes of physical activity (2 intervention groups, 79% positive and statistically significant), and for structured exercise on functional ability: physical domain (23 intervention groups, 50% positive and statistically significant). Recreation/sport had positive impacts on falls (4 intervention groups, 67% positive and statistically significant) and functional ability: physical domain (7 intervention groups, 90% positive) in adults with physical impairments/limitations.

### Physical activity in adults with mild cognitive impairment or low mood

In adults with mild cognitive impairment or low mood (11 intervention groups, 79 outcomes in total) structured exercise had positive effects on most outcome domains except social outcomes which were not commonly investigated (Appendix 11: Figure A.11, Table A.11). Statistical significance was reached in ≥ 50% of comparisons for the impact of structured exercise on functional ability: physical domain (3 intervention groups), cognitive and emotional outcomes (6 intervention groups) and quality of life (1 intervention group), and for recreation/sport on cognitive and emotional outcomes (3 intervention groups).

#### Physical activity in different locations

Physical activity interventions undertaken in community facilities (45 intervention groups, 391 outcomes) and people’s own homes (24 intervention groups, 230 outcomes) had positive impacts (≥ 50% of outcomes positive) across most outcomes except social domain (Appendix 12: Figure A.12, Table A.12). Physical activity delivered in outpatient health facilities was investigated in 4 intervention groups with ≥ 50% outcomes statistically significant in domains of physical activity, functional ability: physical and social domains (1 intervention group each), and cognitive and emotional outcomes (2 intervention groups). Physical activity undertaken in residential aged care facilities had positive impacts on falls (3 intervention groups, 100% positive) and functional ability: physical domain (11 intervention groups, 50% positive). Physical activity undertaken in retirement villages (4 intervention groups) had moderately positive effects across most outcomes except quality of life.

#### Types of structured exercise

**Fig. 4:**
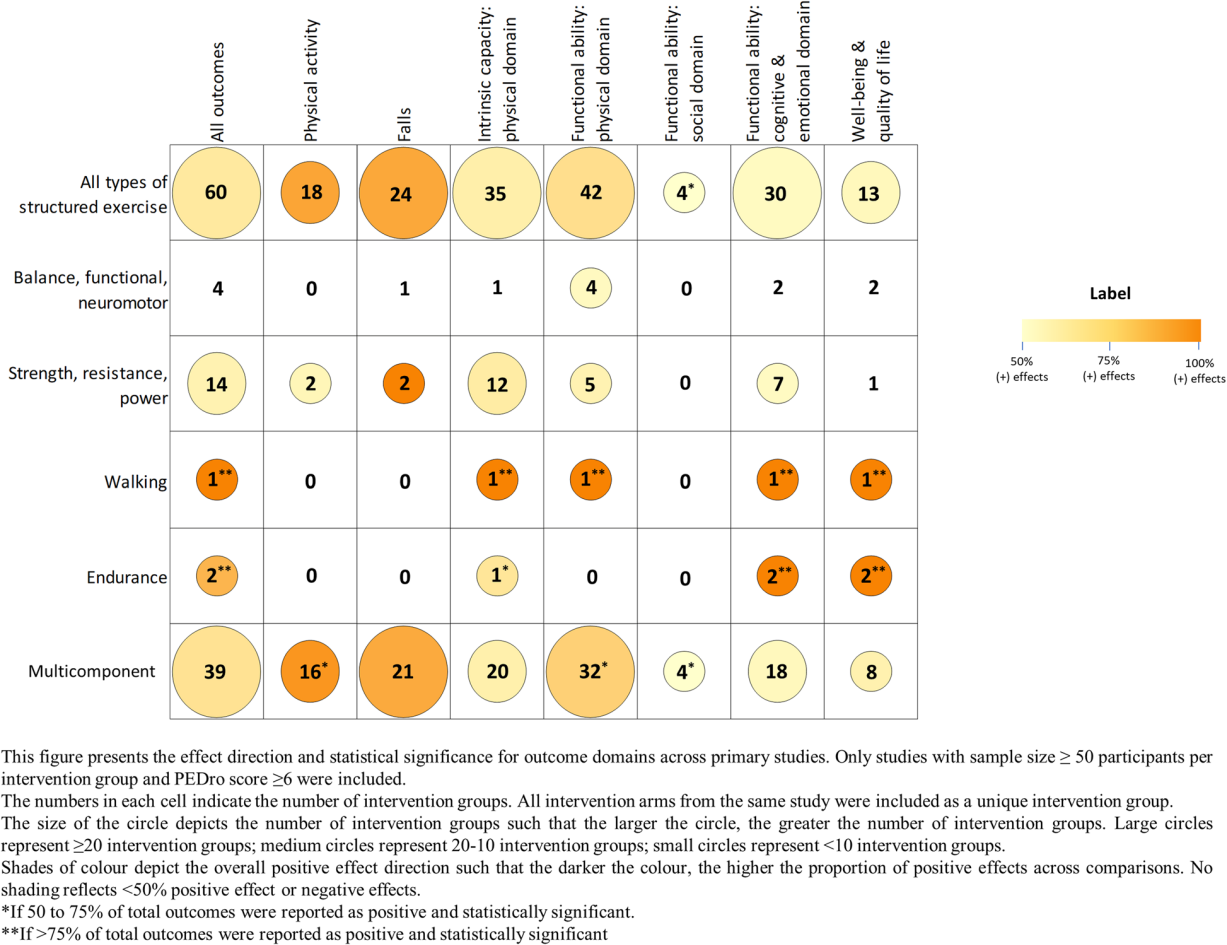
Physical activity interventions for older adults by type of structured exercise: impact on different outcome domains

Multicomponent exercise, defined in our framework as structured exercise programs containing more than one type of exercise, was the most investigated type of structured exercise (39 intervention groups, 398 outcomes) (Fig. 4, Appendix 9: Table A.9.2). Positive effects for multicomponent exercise were seen across all outcome domains, with particularly strong impacts on physical activity (16 intervention groups, 50% of outcomes statistically significant), falls (21 intervention groups, 90% positive) and functional ability: physical domain (32 intervention groups, 81% statistically significant). Walking and endurance interventions had strongly positive effects on intrinsic capacity: physical domain, cognitive and emotional functioning, and quality of life (≥ 50% outcomes statistically significant). No studies investigated interventions for individuals with wheelchair mobility. Strength training as a single form of exercise also showed positive impacts across most outcome domains except social and quality of life, while balance training alone only had positive impacts on functional ability: physical domain (4 intervention groups, 56% positive). Evidence for structured exercise in community facilities, homes, outpatient health facilities, residential aged care facilities and retirement villages, is presented in Appendix 13: Figures A.13.1- A.13.5, Tables A.13.1 – A.13.5.

**Fig. 5:**
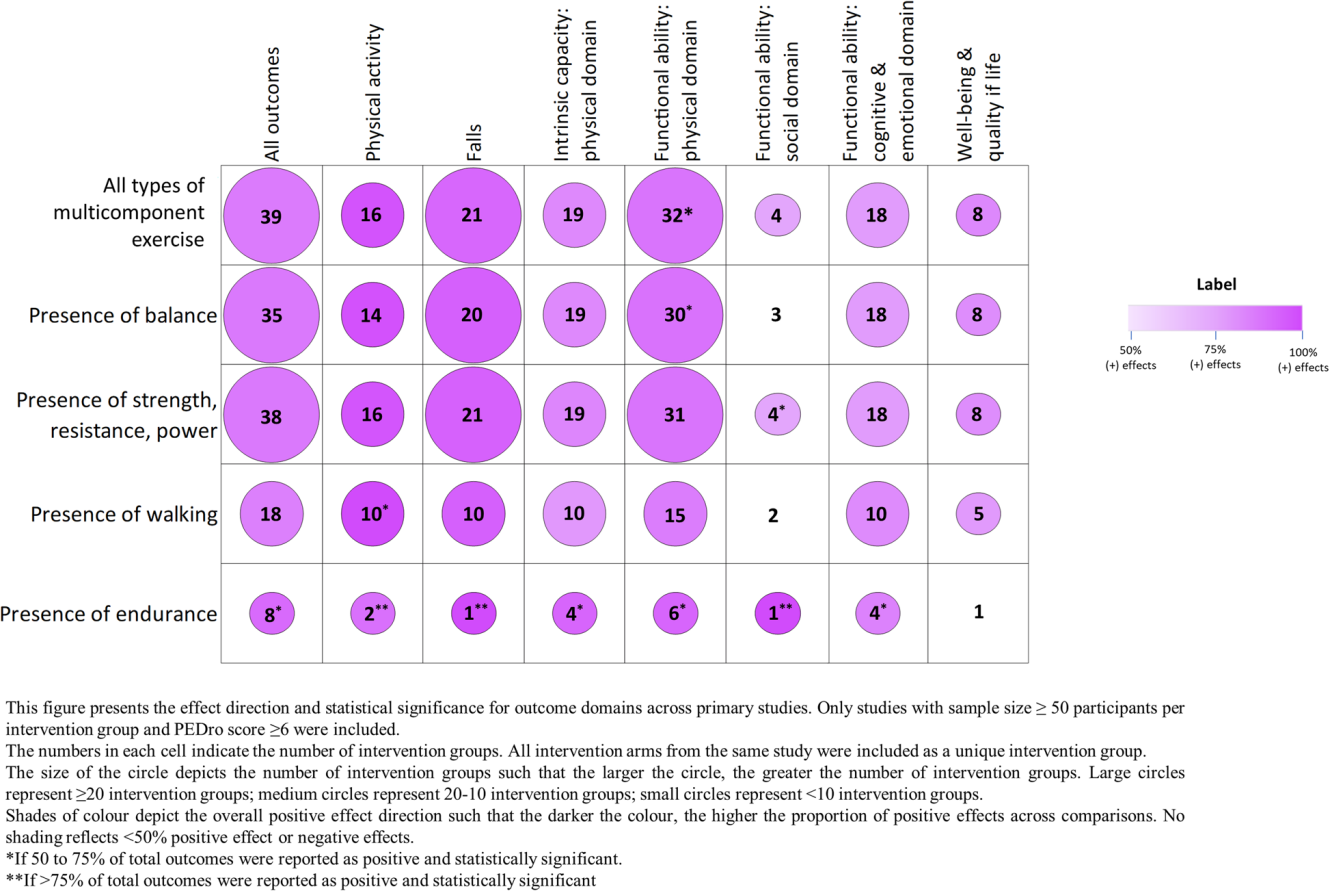
Physical activity interventions for older adults by type of multicomponent exercise: impact on different outcome domains

When the components of multicomponent exercise interventions were analysed, positive impacts were seen across all outcomes for programs that included strength (38 intervention groups, ≥ 50% positive). Positive effects were also evident for most outcomes from programs that included balance (35 intervention groups), walking (18 intervention groups) and endurance (8 intervention groups) (Fig. 5, Appendix 9: Table A.9.3).

#### Types of recreation/sport

**Fig. 6:**
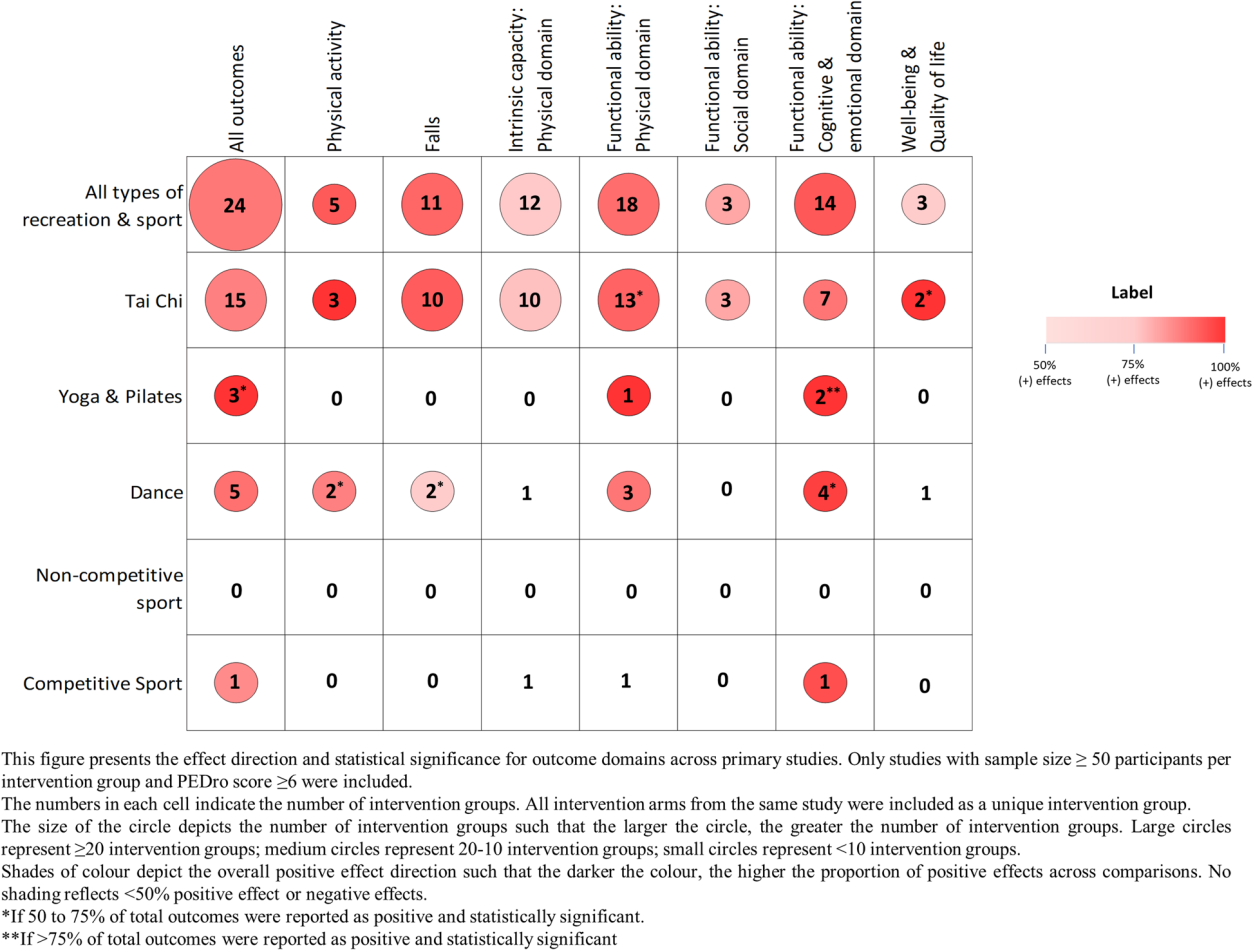
Physical activity interventions for older adults by type of recreation/sport: impact on different outcome domains

Tai chi (15 intervention groups) had strong positive impacts across all outcomes, reaching statistical significance in ≥ 50% outcomes for functional ability: physical domain (13 intervention groups) and quality of life (2 intervention groups) (Fig. 6, Appendix 9: Table A.9.4). Yoga/Pilates (3 intervention groups) had positive effects on functional ability: physical domain (1 intervention group) and cognitive and emotional outcomes (2 intervention groups, 100% statistically significant). Dance (5 intervention groups) was effective for outcomes of physical activity and falls (2 intervention groups each, ≥ 50% statistically significant), functional ability: physical domain (3 intervention groups, 78% positive) and cognitive and emotional outcomes (4 intervention groups, 56% statistically significant), although no impact was seen for intrinsic capacity: physical domain or quality of life. Competitive sport had positive impacts on cognitive and emotional outcomes (1 intervention group, 91% outcomes positive), although no impact on the physical domains of intrinsic capacity or functional ability. Results for recreation/sport in community facilities, residential aged care facilities and retirement villages are presented in Appendix 14: Figures A.14.1 – A.14.3, Tables A.14.1 – A.14.3).

## Discussion

We identified 1421 studies evaluating physical activity programs/services for older adults, although only 87 of these studies were large, good quality RCTs. Overall, the evidence supporting physical activity was positive. Positive effects were seen in ≥ 50% of all outcomes for structured exercise, recreation/sport and interventions promoting overall activity, with multicomponent exercise identified as a particularly effective form of structured exercise. We have presented results for different populations, including older adults with physical impairments and mild cognitive impairments or low mood, as well as different locations. These findings will be valuable for program providers and policymakers when planning and implementing physical activity programs/services.

### Implications for practice

While the evidence in this review was positive in favour of physical activity programs and services overall, different impacts were seen across different physical activity types, populations, and locations. It is therefore important for policymakers and program providers to consider the context and desired outcomes when planning a physical activity program or service. For structured exercise, the strongest impacts were on falls and the physical domains of intrinsic capacity and functional ability. There was strong evidence for multicomponent exercise that included balance or strength exercises across all outcome domains, except social outcomes. For recreation/sport, the strongest impact was on cognitive and emotional functioning. For interventions promoting overall activity, the strongest positive impact was on physical activity and social outcomes. Positive impacts were also seen across all outcome domains for older adults with physical limitations and mild cognitive impairment/low mood, except social outcomes which were not investigated. Physical activity programs/services also had positive impacts across diverse locations, including community facilities, people’s own homes, outpatient health facilities, residential aged care facilities and retirement villages.

Many older adults have chronic health conditions [126]. As we were seeking to inform interventions for broad groups of older adults rather than people with particular conditions, we did not include studies in which all participants had been diagnosed with particular health conditions (e.g., stroke, heart failure). Many of the participants in the included studies would have had various health conditions so the findings are still relevant for adults with health conditions. We also considered the functional impact of a health condition to be more important than the condition itself.

For the present review we did not differentiate outcome according to measurement tool. Different tools measure different aspects of outcomes (e.g., self-reported versus device-based physical activity) and may have different psychometric properties (such as validity, reliability, responsiveness) and different inherent biases (possibility for blinding of assessors to intervention group allocation, greater risk of loss to follow up from more inconvenient measures). Given the importance of these issues we included measurement tools in our framework but considering the broad scope and aim of this study—to provide information about effectiveness of physical activity programs – we did not extract information regarding measurement tools from individual studies included in our review. Furthermore, as our aim was, we did not include studies that primarily sought to implement physical activity interventions previously found to be effective rather than establishing the effectiveness of an intervention. A recent review [127] identified 137 studies about implementation of physical activity interventions for older adults and called for implementation research that extends beyond analysis at an individual level.

### Perspectives for future research

Our review highlights several gaps in current literature. Further primary studies are required in a number of areas: i) sport interventions (only one high-quality, large clinical trial investigating the impact of sport in people aged ≥ 60 years was identified); ii) social and wellbeing outcomes (no studies were found investigating the impact of physical activity on wellbeing outcomes in older adults, and no studies investigated social outcomes for older adults with physical impairments, mild cognitive impairment or mood symptoms); iii) low and lower-middle income countries (no studies investigated structured exercise and overall activity in lower-middle income countries, and no studies were conducted in low income countries); iv) physical activity in underserved/disadvantaged populations (no studies were identified that investigated physical activity in culturally or linguistically diverse populations, migrant or indigenous populations, socio-economically disadvantaged populations or rural/remote settings, or interventions for older adults with wheelchair mobility), and v) the effectiveness of physical activity programs in the oldest age group ≥ 80 years (four out of 87 studies focused on participants aged ≥ 80 years).

### Limitations

We endeavoured to provide a rapid, broad but rigorous overview of the evidence to guide physical activity approaches for older adults around the world. There are inherent limitations of this approach compared to detailed systematic reviews that focus on particular interventions, outcomes, or settings. These limitations apply to the approach we took to searching, classifying, and synthesising evidence. First, we mostly searched for reviews rather than primary studies for this review to be comprehensive in types of activities, settings and outcomes covered. The exception to this was sport, for which we did not identify any reviews, so we did search for primary studies. We are confident we identified the bulk of important studies; however, we could have missed studies. There is also the likelihood of publication bias favouring physical activity benefits within studies we identified. Second, we classified studies using the framework we developed in consultation with experts in the field and WHO. We were only able to classify interventions based on what was reported in the articles, so may have misinterpreted elements, or made incorrect assumptions. We did not classify specific exercise types, such as different strengthening exercises, nor did we classify exercise prescription, intensity, or progression. We also did not classify studies according to measurement tool, which could have affected overall results as different measurement tools have different properties such as the ability to detect change. Future reviews could investigate these components in greater detail. Our definition of multicomponent exercise (as exercise containing two or more types of structured exercise) differs to that of a previous Cochrane review of exercise for preventing falls where to be classified as multicomponent a program had to meet the definitions from the ProFaNE taxonomy [13] for more than one type of exercise, and had to equally focus on two or more components [128]. Hence, given that we classified a high number of interventions as ‘multicomponent’ in the current review, it is possible that some of these interventions could be re-classified as another type of structured exercise (e.g. strength/resistance/power or balance/functional/neuromotor) using the definition from the Cochrane review. However, our definition of multicomponent exercise is consistent with the WHO *Guidelines on Physical Activity and Sedentary Behaviour *[2] which emphasise incorporating strength and balance exercises for health benefits. Future reviews could also focus on primary studies reported in languages other than English, as we only included reviews written in English, which could have contributed to the lack of studies from low and lower-middle income countries. Effect sizes for interventions could also be investigated as it is likely that some interventions have bigger effects (i.e., larger improvements compared to control) than others.

Third, we did not undertake meta-analysis. Instead, we used a form of ‘vote-counting’ to report the proportion of outcomes for which comparisons between groups were positive. The Cochrane Collaboration Handbook cautions against traditional vote counting which only considers the statistical significance of comparisons, as many trials will be underpowered so potentially important effects could be missed [17]. Rather, we estimated the proportion of effects in individual trials that were positive. Our figures show the outcome domains for which ≥ 50% of outcomes in individual studies were positive for a particular combination of population, location and type of intervention. As between-group comparisons in trials are seeking to estimate ‘true’ effects of interventions, if an intervention has a ‘true’ positive effect, more of the effects found in individual trials would be on the positive rather than negative side of the point of no effect (zero between group difference). The approach we have taken is considered an ‘acceptable’ form of vote counting [17]. We acknowledge that as we did not consider effect sizes this approach remains inferior to meta-analysis. This rapid review provides a platform for future meta-analyses. Finally, our findings do not enable program providers to make decisions about weighing up the benefits of programs against program costs and efforts. Nonetheless we consider the breadth of programs, settings and outcomes summarised in our review to illustrate the benefits of the approach we have taken.

## Conclusions

We identified 1421 physical activity intervention studies, although studies were generally small and of poor/fair quality. Overall, findings from the 87 good quality large trials were positive. Positive impacts were evident for overall activity, structured exercise, and recreation/sport across the outcomes of physical activity, falls, intrinsic capacity (physical domain), functional ability (physical, social and cognitive and emotional domains) and quality of life. The evidence presented in this review for different populations and settings will provide guidance for developing physical activity programs for different populations, locations and settings globally.

## Supplementary Information

**Additional file 1:** PRISMA-ScR_checklist

**Additional file 2:** **Appendix 1.** Eligibility criteriaand search strategies used to identify systematic reviews. **Appendix 2.** Flow chart ofselection of reviews from the updated search. **Appendix 3.** Systematic reviewsidentified in the updated search. **Appendix 4.** Search strategies andflow chart of the selection of primary studies investigating sports for older adults. **Appendix 5.** Eligibility criteriafor selection of primary studies. **Appendix 6.** Overview of relevant concepts incategorising outcomes of physical activity interventions for older adults. **Appendix 7.** Overview of primary studies ofphysical activity programs and services for older adults included in Objective2 (k=87 studies). **Appendix 8.** Description of studiesincluded in Objective 2 according to intervention location. **Appendix 9.** Dataused to create Figures 2-5 presenting the evidence of physical activityservices and programs for older adults. **Appendix 10.** Impact of physical activity typeson different outcome domains in adults with physical impairments. **Appendix 11.** Impact of physicalactivity types on outcome domains in adults with mild cognitive impairment orlow mood. **Appendix 12.** Impact of physicalactivity on different outcome domains by location. **Appendix 13.** Impact of structuredexercise in different locations on different outcome domains. **Appendix 14.** Impact ofrecreation/sport in different locations on different outcome domains. 

**Additional file 3:** 

## Data Availability

Data are available in a searchable Excel database which can be downloaded at https://crepreventfallsinjuries.org.au/publications/.

## Abbreviations

CONSORT: Consolidated Standards of Reporting Trials
GAPPA: Global Action Plan on Physical Activity
ProFaNE: Prevention of Falls Network Europe
TIDieR: Template for Intervention Description and Replication
WHO: World Health Organization

## References

[1] World Health Organization (2017). Global strategy and action plan on ageing and health.

[2] World Health Organization. WHO guidelines on physical activity and sedentary behaviour. World Health Organization (WHO); 2020. 33369898

[3] Bauman A, et al. Updating the evidence for physical activity: summative reviews of the epidemiological evidence, prevalence, and interventions to promote “active aging.” Gerontologist. 2016;56(Suppl 2):S268–80. 10.1093/geront/gnw031 26994266

[4] World Health Organization. More active people for a healthier world. World Health Organization (WHO); 2018.

[5] World Health Organization. Active: a technical package for increasing physical activity. 2018.

[6] Taylor J (2021). A scoping review of physical activity interventions for older adults. Int J Behav Nutr Phys Act.

[7] Moher D, et al. Preferred reporting items for systematic reviews and meta-analyses: the PRISMA statement. PLoS Med. 2009;6(7):e1000097. 10.1371/journal.pmed.1000097 PMC2707599 19621072

[8] Pinheiro MB. Systematic review of physical activity interventions for older adults: protocol. 2021. Available from: https://bit.ly/2W00Q6U. [cited 2021 7 October].

[9] Maher CG (2003). Reliability of the PEDro scale for rating quality of randomized controlled trials. Phys Ther.

[10] Sterne JA, Gavaghan D, Egger M (2000). Publication and related bias in meta-analysis: power of statistical tests and prevalence in the literature. J Clin Epidemiol.

[11] Cashin AG, McAuley JH (2020). Clinimetrics: Physiotherapy Evidence Database (PEDro) Scale. J Physiother.

[12] Hoffmann TC, et al. Better reporting of interventions: template for intervention description and replication (TIDieR) checklist and guide. BMJ. 2014;348:g1687. 10.1136/bmj.g1687 24609605

[13] Lamb SE (2005). Development of a common outcome data set for fall injury prevention trials: the prevention of falls network Europe consensus. J Am Geriatr Soc.

[14] World Health Organization. World report on ageing and health. World Health Organization (WHO); 2015.

[15] World Health Organization (2020). Decade of healthy ageing: baseline report.

[16] WHO. International Classification of Functioning, Disability and Health (ICF). World Health Organization (WHO); 2018.

[17] McKenzie JE, Brennan SE. Chapter 12: Synthesizing and presenting findings using other methods. In: Cochrane Handbook for Systematic Reviews of Interventions version 6.2. 2021.

[18] McKenzie JE, Brennan SE. Synthesizing and presenting findings using other methods. In: Higgins J, et al., editors. Cochrane Handbook for Systematic Reviews of Interventions. Cochrane; 2021. p. 321–47.

[19] Broekhuizen K, de Jelle G, Wijsman CA, Wijsman LW, Westendorp RGJ, Verhagen E, et al. An internet-based physical activity intervention to improve quality of life of inactive older adults: a randomized controlled trial. J Med Internet Res. 2016;18(4):e74. 10.2196/jmir.4335 PMC4917725 27122359

[20] Dubbert PM, Morey MC, Kirchner KA, Meydrech EF, Grothe K (2008). Counseling for home-based walking and strength exercise in older primary care patients. Arch Intern Med..

[21] Herghelegiu AM, Moser A, Prada GI, Born S, Wilhelm M, Stuck AE. Effects of health risk assessment and counselling on physical activity in older people: a pragmatic randomised trial. PLoS One. 2017;12(7):e0181371. 10.1371/journal.pone.0181371 PMC5519086 28727796

[22] Kerse N, Elley CR, Robinson E, Arroll B (2005). Is physical activity counseling effective for older people? A cluster randomized, controlled trial in primary care. J Am Geriatr Soc..

[23] King AC, Friedman R, Marcus B, Castro C, Napolitano M, Ahn D, Baker L (2007). Ongoing physical activity advice by humans versus computers: the Community Health Advice by Telephone (CHAT) trial. Health Psychol..

[24] Kolt GS, Schofield GM, Kerse N, Garrett N, Oliver M (2007). Effect of telephone counseling on physical activity for low-active older people in primary care: a randomized, controlled trial. J Am Geriatr Soc..

[25] Komulainen P (2021). Exercise, diet, and cognition in a 4-year randomized controlled trial: Dose-Responses to Exercise Training (DR’s EXTRA). Am J Clin Nutr..

[26] Lautenschlager NT, Cox KL, Flicker L, Foster JK, van Bockxmeer FM, Xiao J, Greenop KR, Almeida OP (2008). Effect of physical activity on cognitive function in older adults at risk for Alzheimer disease: a randomized trial. JAMA..

[27] Manty M, Heinonen A, Leinonen R, Tormakangas T, Hirvensalo M, Kallinen M, Sakari R, von Bonsdorff MB, Heikkinen E, Rantanen T (2009). Long-term effect of physical activity counseling on mobility limitation among older people: a randomized controlled study. J Gerontol A Biol Sci Med Sci..

[28] McMurdo ME, Sugden J, Argo I, Boyle P, Johnston DW, Sniehotta FF, Donnan PT (2010). Do pedometers increase physical activity in sedentary older women? A randomized controlled trial. J Am Geriatr Soc..

[29] Morey MC, Ekelund C, Pearson M, Crowley G, Peterson M, Sloane R, Pieper C, McConnell E, Bosworth H (2006). Project LIFE: a partnership to increase physical activity in elders with multiple chronic illnesses. J Aging Phys Act..

[30] Morey MC, Peterson MJ, Pieper CF, Sloane R, Crowley GM, Cowper PA, McConnell ES, Bosworth HB, Ekelund CC, Pearson MP (2009). The Veterans Learning to Improve Fitness and Function in Elders Study: a randomized trial of primary care-based physical activity counseling for older men. J Am Geriatr Soc..

[31] Oliveira JS, Sherrington C, Paul SS, Ramsay E, Chamberlain K, Kirkham C, O'Rourke SD, Hassett L, Tiedemann A (2019). A combined physical activity and fall prevention intervention improved mobility-related goal attainment but not physical activity in older adults: a randomised trial. J Physiother..

[32] Petrella RJ, Koval JJ, Cunningham DA, Paterson DH (2003). Can primary care doctors prescribe exercise to improve fitness? The step test exercise prescription (STEP) project. Am J Prev Med..

[33] Rasinaho M, Hirvensalo M, Törmäkangas T, Leinonen R, Lintunen T, Rantanen T (2012). Effect of physical activity counseling on physical activity of older people in Finland (ISRCTN 07330512). Health Promot Int..

[34] Thomas GN, Macfarlane DJ, Guo B, Cheung BM, McGhee SM, Chou KL, Deeks JJ, Lam TH, Tomlinson B (2012). Health promotion in older Chinese: a 12-month cluster randomized controlled trial of pedometry and "peer support". Med Sci Sports Exerc..

[35] von Bonsdorff MB, Leinonen R, Kujala UM, Heikkinen E, Törmäkangas T, Hirvensalo M, Rasinaho M, Karhula S, Mänty M, Rantanen T (2008). Effect of physical activity counseling on disability in older people: a 2-year randomized controlled trial. J Am Geriatr Soc..

[36] Voukelatos A, Merom D, Sherrington C, Rissel C, Cumming RG, Lord SR (2015). The impact of a home-based walking program on falls in older people: the Easy Steps randomised controlled trial. Age Ageing..

[37] Wijsman CA, Westendorp RG, Verhagen EA, Catt M, Slagboom PE, de Craen AJ, Broekhuizen K, van Mechelen W, van Heemst D, van der Ouderaa F, Mooijaart SP. Effects of a web-based intervention on physical activity and metabolism in older adults: randomized controlled trial. J Med Internet Res. 2013;15(11):e233. 10.2196/jmir.2843 PMC3841355 24195965

[38] Yates T, Edwardson CL, Henson J, Gray LJ, Ashra NB, Troughton J, Khunti K, Davies MJ (2017). Walking Away from Type 2 diabetes: a cluster randomized controlled trial. Diabet Med..

[39] Best JR, Chiu BK, Liang Hsu C, Nagamatsu LS, Liu-Ambrose T (2015). Long-Term Effects of Resistance Exercise Training on Cognition and Brain Volume in Older Women: Results from a Randomized Controlled Trial. J Int Neuropsychol Soc..

[40] Bogaerts AC, Delecluse C, Claessens AL, Troosters T, Boonen S, Verschueren SM (2009). Effects of whole body vibration training on cardiorespiratory fitness and muscle strength in older individuals (a 1-year randomised controlled trial). Age Ageing..

[41] Chandler JM, Duncan PW, Kochersberger G, Studenski S (1998). Is lower extremity strength gain associated with improvement in physical performance and disability in frail, community-dwelling elders?. Arch Phys Med Rehabil..

[42] Chin A Paw MJM, van Poppel MNM, Twisk JWR, van Mechelen W. Effects of resistance and all-round, functional training on quality of life, vitality and depression of older adults living in long-term care facilities: a 'randomized' controlled trial [ISRCTN87177281]. BMC Geriatr. 2004;4:5. 10.1186/1471-2318-4-5 PMC471553 15233841

[43] Chin A Paw MJM, van Poppel MNM, Twisk JWR, van Mechelen W. Once a week not enough, twice a week not feasible? A randomised controlled exercise trial in long-term care facilities [ISRCTN87177281]. Patient Educ Couns. 2006;63(1–2):205–14. 10.1016/j.pec.2005.10.008 16426800

[44] Jette AM, Harris BA, Sleeper L, Lachman ME, Heislein D, Giorgetti M, Levenson C (1996). A home-based exercise program for nondisabled older adults. J Am Geriatr Soc..

[45] Jette AM, Lachman M, Giorgetti MM, Assmann SF, Harris BA, Levenson C, Wernick M, Krebs D (1999). Exercise–it's never too late: the strong-for-life program. Am J Public Health..

[46] Krebs DE, Jette AM, Assmann SF (1998). Moderate exercise improves gait stability in disabled elders. Arch Phys Med Rehabil..

[47] Sparrow D, Gottlieb DJ, Demolles D, Fielding RA (2011). Increases in muscle strength and balance using a resistance training program administered via a telecommunications system in older adults. J Gerontol A Biol Sci Med Sci..

[48] Thomas GN, Hong AW, Tomlinson B, Lau E, Lam CW, Sanderson JE, Woo J (2005). Effects of Tai Chi and resistance training on cardiovascular risk factors in elderly Chinese subjects: a 12-month longitudinal, randomized, controlled intervention study. Clin Endocrinol (Oxf)..

[49] Vogler CM, Sherrington C, Ogle SJ, Lord SR (2009). Reducing risk of falling in older people discharged from hospital: a randomized controlled trial comparing seated exercises, weight-bearing exercises, and social visits. Arch Phys Med Rehabil..

[50] Woo J, Hong A, Lau E, Lynn H (2007). A randomised controlled trial of Tai Chi and resistance exercise on bone health, muscle strength and balance in community-living elderly people. Age Ageing..

[51] Chin A Paw MJM, van Poppel MNM, Twisk JWR, van Mechelen W. Effects of resistance and all-round, functional training on quality of life, vitality and depression of older adults living in long-term care facilities: a 'randomized' controlled trial [ISRCTN87177281]. BMC Geriatr. 2004;4:5. 10.1186/1471-2318-4-5 PMC471553 15233841

[52] Faber MJ, Bosscher RJ, Chin A Paw MJ, van Wieringen PC. Effects of exercise programs on falls and mobility in frail and pre-frail older adults: A multicenter randomized controlled trial. Arch Phys Med Rehabil. 2006;87(7):885–96. 10.1016/j.apmr.2006.04.005 16813773

[53] Peri K, Kerse N, Robinson E, Parsons M, Parsons J, Latham N (2008). Does functionally based activity make a difference to health status and mobility? A randomised controlled trial in residential care facilities (The Promoting Independent Living Study; PILS). Age Ageing..

[54] Maki Y, Ura C, Yamaguchi T, Murai T, Isahai M, Kaiho A, Yamagami T, Tanaka S, Miyamae F, Sugiyama M, Awata S, Takahashi R, Yamaguchi H (2012). Effects of intervention using a community-based walking program for prevention of mental decline: a randomized controlled trial. J Am Geriatr Soc..

[55] Muscari A, Giannoni C, Pierpaoli L, Berzigotti A, Maietta P, Foschi E, Ravaioli C, Poggiopollini G, Bianchi G, Magalotti D, Tentoni C, Zoli M (2010). Chronic endurance exercise training prevents aging-related cognitive decline in healthy older adults: a randomized controlled trial. Int J Geriatr Psychiatry..

[56] Song D, Yu DSF (2019). Effects of a moderate-intensity aerobic exercise program on the cognitive function and quality of life of community-dwelling elderly people with mild cognitive impairment: A randomised controlled trial. Int J Nurs Stud..

[57] Barban F, Annicchiarico R, Melideo M, Federici A, Lombardi MG, Giuli S, et al. Reducing Fall Risk with Combined Motor and Cognitive Training in Elderly Fallers. Brain Sci. 2017;7(2). 10.3390/brainsci7020019 PMC5332962 28208604

[58] Barnett A, Smith B, Lord SR, Williams M, Baumand A (2003). Community-based group exercise improves balance and reduces falls in at-risk older people: a randomised controlled trial. Age Ageing..

[59] Bernocchi P, Giordano A, Pintavalle G, Galli T, Ballini Spoglia E, Baratti D, et al. Feasibility and Clinical Efficacy of a Multidisciplinary Home-Telehealth Program to Prevent Falls in Older Adults: A Randomized Controlled Trial. J Am Med Dir Assoc. 2019;20(3):340–6. 10.1016/j.jamda.2018.09.003 30366759

[60] Boongird C, Keesukphan P, Phiphadthakusolkul S, Rattanasiri S, Thakkinstian A. Effects of a simple home-based exercise program on fall prevention in older adults: A 12-month primary care setting, randomized controlled trial. Geriatr Gerontol Int. 2017;17(11):2157–63. 10.1111/ggi.13052 28436154

[61] Campbell AJ, Robertson MC, Gardner MM, Norton RN, Tilyard MW, Buchner DM. Randomised controlled trial of a general practice program of home based exercise to prevent falls in elderly women. Bmj. 1997;315(7115):1065–9. 10.1136/bmj.315.7115.1065 PMC2127698 9366737

[62] Carlsson M, Littbrand H, Gustafson Y, Lundin-Olsson L, Lindelöf N, Rosendahl E, et al. Effects of high-intensity exercise and protein supplement on muscle mass in ADL dependent older people with and without malnutrition: a randomized controlled trial. J Nutr Health Aging. 2011;15(7):554–60.10.1007/s12603-011-0017-521808934

[63] Clemson L, Fiatarone Singh MA, Bundy A, Cumming RG, Manollaras K, O’Loughlin P, et al. Integration of balance and strength training into daily life activity to reduce rate of falls in older people (the LiFE study): randomised parallel trial. BMJ. 2012;345:e4547. 10.1136/bmj.e4547 PMC3413733 22872695

[64] Conradsson M, Littbrand H, Lindelof N, Gustafson Y, Rosendahl E. Effects of a high-intensity functional exercise program on depressive symptoms and psychological well-being among older people living in residential care facilities: A cluster-randomized controlled trial. Aging Ment Health. 2010;14(5):565–76. 10.1080/13607860903483078 20496181

[65] Day L, Fildes B, Gordon I, Fitzharris M, Flamer H, Lord S. Randomised factorial trial of falls prevention among older people living in their own homes. BMJ. 2002;325(7356):128–31. 10.1136/bmj.325.7356.128 PMC117228 12130606

[66] Duckham RL, Masud T, Taylor R, Kendrick D, Carpenter H, Iliffe S, et al. Randomised controlled trial of the effectiveness of community group and home-based falls prevention exercise programs on bone health in older people: the ProAct65+ bone study. Age Ageing. 2015;44(4):573–9. 10.1093/ageing/afv055 PMC4476850 25906791

[67] El-Khoury F, Cassou B, Latouche A, Aegerter P, Charles MA, Dargent-Molina P. Effectiveness of two year balance training program on prevention of fall induced injuries in at risk women aged 75–85 living in community: Ossébo randomised controlled trial. Bmj. 2015;351:h3830. 10.1136/bmj.h3830 PMC4511529 26201510

[68] Freiberger E, Häberle L, Spirduso WW, Rixt Zijlstra GA. Long‐Term Effects of Three Multicomponent Exercise Interventions on Physical Performance and Fall‐Related Psychological Outcomes in Community‐Dwelling Older Adults: A Randomized Controlled Trial. J Am Geriatr Soc. 2012;60(3):437–46. 10.1111/j.1532-5415.2011.03859.x 22324753

[69] Gill TM, Pahor M, Guralnik JM, McDermott MM, King AC, Buford TW, et al. Effect of structured physical activity on prevention of serious fall injuries in adults aged 70–89: randomized clinical trial (LIFE Study). Bmj. 2016;352:i245. 10.1136/bmj.i245 PMC4772786 26842425

[70] Gschwind YJ, Eichberg S, Ejupi A, de Rosario H, Kroll M, Marston HR, et al. ICT-based system to predict and prevent falls (iStoppFalls): results from an international multicenter randomized controlled trial. Eur Rev Aging Phys Act. 2015;12:10. 10.1186/s11556-015-0155-6 PMC4748323 26865874

[71] Hewitt J, Goodall S, Clemson L, Henwood T, Refshauge K (2018). Progressive Resistance and Balance Training for Falls Prevention in Long-Term Residential Aged Care: A Cluster Randomized Trial of the Sunbeam Program. J Am Med Dir Assoc..

[72] Hsieh T-J, Su S-C, Chen C-W, Kang Y-W, Hu M-H, Hsu L-L, Wu S-Y, Chen L, Chang H-Y, Chuang S-Y, Pan W-H, Hsu C-C (2019). Individualized home-based exercise and nutrition interventions improve frailty in older adults: a randomized controlled trial. Int J Behav Nutr Phys Act..

[73] Iliffe S, Kendrick D, Morris R, Masud T, Gage H, Skelton D, et al. Multicentre cluster randomised trial comparing a community group exercise program and home-based exercise with usual care for people aged 65 years and over in primary care. Health Technol Assess. 2014;18(49):vii-xxvii, 1–105. 10.3310/hta18490 PMC4781144 25098959

[74] King AC, Pruitt LA, Phillips W, Oka R, Rodenburg A, Haskell WL. Comparative effects of two physical activity programs on measured and perceived physical functioning and other health-related quality of life outcomes in older adults. J Gerontol A Biol Sci Med Sci. 2000;55(2):M74–83. 10.1093/gerona/55.2.m74 10737689

[75] Klusmann V, Evers A, Schwarzer R, Schlattmann P, Reischies FM, Heuser I, et al. Complex mental and physical activity in older women and cognitive performance: a 6-month randomized controlled trial. J Gerontol A Biol Sci Med Sci. 2010;65(6):680–8. 10.1093/gerona/glq053 20418350

[76] Korpelainen R, Keinänen-Kiukaanniemi S, Heikkinen J, Vaananen K, Korpelainen J. Effect of exercise on extraskeletal risk factors for hip fractures in elderly women with low BMD: a population-based randomized controlled trial. J Bone Miner Res. 2006;21(5):772–9. 10.1359/jbmr.060116 16734393

[77] Korpelainen R, Keinänen-Kiukaanniemi S, Heikkinen J, Väänänen K, Korpelainen J. Effect of impact exercise on bone mineral density in elderly women with low BMD: a population-based randomized controlled 30-month intervention. Osteoporos Int. 2006;17(1):109–18. 10.1007/s00198-005-1924-2 15889312

[78] Li F, Harmer P, Fitzgerald K, Eckstrom E, Akers L, Chou LS, et al. Effectiveness of a therapeutic Tai Ji Quan intervention versus a multimodal exercise intervention to prevent falls among older adults at high risk of falling: a randomized clinical trial [with consumer summary]. JAMA Intern Med. 2018;178(10):1301–10. 10.1001/jamainternmed.2018.3915 PMC6233748 30208396

[79] Life Study Investigators; Pahor M, Blair SN, Espeland M, Fielding R, Gill TM, et al. Effects of a physical activity intervention on measures of physical performance: Results of the lifestyle interventions and independence for Elders Pilot (LIFE-P) study. J Gerontol A Biol Sci Med Sci. 2006;61(11):1157–65. 10.1093/gerona/61.11.1157 17167156

[80] Liu-Ambrose T, Davis JC, Best JR, Dian L, Madden K, Cook W, et al. Effect of a Home-Based Exercise Program on Subsequent Falls Among Community-Dwelling High-Risk Older Adults After a Fall: A Randomized Clinical Trial. Jama. 2019;321(21):2092–100. 10.1001/jama.2019.5795 PMC6549299 31162569

[81] Lord SR, Castell S, Corcoran J, Dayhew J, Matters B, Shan A, et al. The effect of group exercise on physical functioning and falls in frail older people living in retirement villages: a randomized, controlled trial. J Am Geriatr Soc. 2003;51(12):1685–92. 10.1046/j.1532-5415.2003.51551.x 14687345

[82] Luukinen H, Lehtola S, Jokelainen J, Vaananen-Sainio R, Lotvonen S, Koistinen P. Prevention of disability by exercise among the elderly: a population-based, randomized, controlled trial. Scand J Prim Health Care. 2006;24(4):199–205. 10.1080/02813430600958476 17118858

[83] Luukinen H, Lehtola S, Jokelainen J, Väänänen-Sainio R, Lotvonen S, Koistinen P. Pragmatic exercise-oriented prevention of falls among the elderly: a population-based, randomized, controlled trial. Prev Med. 2007;44(3):265–71. 10.1016/j.ypmed.2006.09.011 17174387

[84] Martin-Borras C, Giné-Garriga M, Puig-Ribera A, Martin C, Sola M, Cuesta-Vargas AI; Ppaf Group. A new model of exercise referral scheme in primary care: is the effect on adherence to physical activity sustainable in the long term? A 15-month randomised controlled trial. BMJ Open. 2018;8(3):e017211. 10.1136/bmjopen-2017-017211 PMC5855315 29502081

[85] Mulrow CD, Gerety MB, Kanten D, Cornell JE, DeNino LA, Chiodo L, et al. A randomized trial of physical rehabilitation for very frail nursing home residents. Jama. 1994;271(7):519–24. 8301766

[86] Pahor M, Guralnik JM, Ambrosius WT, Blair S, Bonds DE, Church TS, et al. Life study investigators, Effect of structured physical activity on prevention of major mobility disability in older adults: the LIFE study randomized clinical trial. JAMA. 2014;311(23):2387–96. 10.1001/jama.2014.5616 PMC4266388 24866862

[87] Robertson MC, Devlin N, Gardner MM, Campbell AJ. Effectiveness and economic evaluation of a nurse delivered home exercise program to prevent falls. 1: Randomised controlled trial. BMJ. 2001;322(7288):697–701. 10.1136/bmj.322.7288.697 PMC30094 11264206

[88] Rosendahl E, Gustafson Y, Nordin E, Lundin-Olsson L, Nyberg L. A randomized controlled trial of fall prevention by a high-intensity functional exercise program for older people living in residential care facilities. Aging Clin Exp Res. 2008;20(1):67–75. 10.1007/BF03324750 18283231

[89] Rosendahl E, Lindelöf N, Littbrand H, Yifter-Lindgren E, Lundin-Olsson L, Håglin L, et al. High-intensity functional exercise program and protein-enriched energy supplement for older persons dependent in activities of daily living: a randomised controlled trial. Aust J Physiother. 2006;52(2):105–13. 10.1016/s0004-9514(06)70045-9 16764547

[90] Sherrington C, Lord SR, Vogler CM, Close JC, Howard K, Dean CM, et al. A post-hospital home exercise program improved mobility but increased falls in older people: a randomised controlled trial. PLoS One. 2014;9(9):e104412. 10.1371/journal.pone.0104412 PMC4152130 25180702

[91] Sherrington C, Pamphlett PI, Jacka JA, Olivetti LM, Nugent JA, Hall JM, et al. Group exercise can improve participants' mobility in an outpatient rehabilitation setting: a randomized controlled trial. Clin Rehabil. 2008;22(6):493–502. 10.1177/0269215508087994 18511529

[92] Shimada H, Makizako H, Doi T, Park H, Tsutsumimoto K, Verghese J, et al. Effects of Combined Physical and Cognitive Exercises on Cognition and Mobility in Patients With Mild Cognitive Impairment: A Randomized Clinical Trial. J Am Med Dir Assoc. 2018;19(7):584–91. 10.1016/j.jamda.2017.09.019 29153754

[93] Shumway-Cook A, Silver IF, LeMier M, York S, Cummings P, Koepsell TD. Effectiveness of a community-based multifactorial intervention on falls and fall risk factors in community-living older adults: a randomized, controlled trial. J Gerontol A Biol Sci Med Sci. 2007;62(12):1420–7. 10.1093/gerona/62.12.1420 18166695

[94] Sink KM, Espeland MA, Castro CM, Church T, Cohen R, Dodson JA, et al. Effect of a 24-Month Physical Activity Intervention vs Health Education on Cognitive Outcomes in Sedentary Older Adults: The LIFE Randomized Trial. JAMA. 2015;314(8):781–90. 10.1001/jama.2015.9617 PMC4698980 26305648

[95] Suzuki T, Shimada H, Makizako H, Doi T, Yoshida D, Ito K, et al. A randomized controlled trial of multicomponent exercise in older adults with mild cognitive impairment. PLoS One. 2013;8(4):e61483. 10.1371/journal.pone.0061483 PMC3621765 23585901

[96] Trombetti A, Hars M, Hsu FC, Reid KF, Church TS, Gill TM, et al. Effect of Physical Activity on Frailty: Secondary Analysis of a Randomized Controlled Trial. Ann Intern Med. 2018;168(5):309–16. 10.7326/M16-2011 PMC5898617 29310138

[97] Uemura K, Doi T, Shimada H, Makizako H, Yoshida D, Tsutsumimoto K, et al. Effects of exercise intervention on vascular risk factors in older adults with mild cognitive impairment: a randomized controlled trial. Dement Geriatr Cogn Dis Extra. 2012;2(1):445–55. 10.1159/000343486 PMC3507268 23189083

[98] Williamson JD, Espeland M, Kritchevsky SB, Newman AB, King AC, Pahor M, et al. Changes in cognitive function in a randomized trial of physical activity: results of the lifestyle interventions and independence for elders pilot study. J Gerontol A Biol Sci Med Sci. 2009;64(6):688–94. 10.1093/gerona/glp014 PMC2679423 19244157

[99] Yang XJ, Hill K, Moore K, Williams S, Dowson L, Borschmann K, et al. Effectiveness of a targeted exercise intervention in reversing older people's mild balance dysfunction: a randomized controlled trial. Phys Ther. 2012;92(1):24–37. 10.2522/ptj.20100289 21979272

[100] Day L, Hill KD, Stathakis VZ, Flicker L, Segal L, Cicuttini F, et al. Impact of Tai-Chi on Falls Among Preclinically Disabled Older People. A Randomized Controlled Trial. J Am Med Dir Assoc. 2015;16(5):420–6. 10.1016/j.jamda.2015.01.089 25769960

[101] Day L, Hill KD, Jolley D, Cicuttini F, Flicker L, Segal L (2012). Impact of tai chi on impairment, functional limitation, and disability among preclinically disabled older people: a randomized controlled trial. Arch Phys Med Rehabil..

[102] Fan B, Song W, Zhang J, Er Y, Xie B, Zhang H, Liao Y, Wang C, Hu X, Mcintyre R (2020). The efficacy of mind-body (Baduanjin) exercise on self-reported sleep quality and quality of life in elderly subjects with sleep disturbances: a randomized controlled trial. Sleep Breath..

[103] Greenspan AI, Wolf SL, Kelley ME, O'Grady M (2007). Tai chi and perceived health status in older adults who are transitionally frail: a randomized controlled trial. Phys Ther..

[104] Lam L, Chan WM, Kwok TC, Chiu HF (2014). Effectiveness of Tai Chi in maintenance of cognitive and functional abilities in mild cognitive impairment: a randomised controlled trial. Hong Kong Med J..

[105] Lipsitz LA, Macklin EA, Travison TG, Manor B, Gagnon P, Tsai T, et al. A Cluster Randomized Trial of Tai Chi vs Health Education in Subsidized Housing: The MI‐WiSH Study. J Am Geriatr Soc. 2019;67(9):1812–9. 10.1111/jgs.15986 PMC6732029 31116883

[106] Logghe IH, Zeeuwe PE, Verhagen AP, Wijnen-Sponselee RM, Willemsen SP, Bierma-Zeinstra SM, et al. Lack of effect of Tai Chi Chuan in preventing falls in elderly people living at home: a randomized clinical trial. J Am Geriatr Soc. 2009;57(1):70–5. 10.1111/j.1532-5415.2008.02064.x 19054193

[107] Sattin RW, Easley KA, Wolf SL, Chen Y, Kutner MH. Reduction in fear of falling through intense tai chi exercise training in older, transitionally frail adults. J Am Geriatr Soc. 2005;53(7):1168–78. 10.1111/j.1532-5415.2005.53375.x 16108935

[108] Sun J, Kanagawa K, Sasaki J, Ooki S, Xu H, Wang L. Tai chi improves cognitive and physical function in the elderly: a randomized controlled trial. J Phys Ther Sci. 2015;27(5):1467–71. 10.1589/jpts.27.1467 PMC4483420 26157242

[109] Tajik A, Rejeh N, Heravi-Karimooi M, Samady Kia P, Tadrisi SD, Watts TE, et al. The effect of Tai Chi on quality of life in male older people: A randomized controlled clinical trial. Complement Ther Clin Pract. 2018;33:191–6. 10.1016/j.ctcp.2018.10.009 30396620

[110] Taylor D, Hale L, Schluter P, Waters DL, Binns EE, McCracken H, et al. Effectiveness of tai chi as a community-based falls prevention intervention: a randomized controlled trial. J Am Geriatr Soc. 2012;60(5):841–8. 10.1111/j.1532-5415.2012.03928.x 22587850

[111] Tsang HW, Lee JL, Au DW, Wong KK, Lai KW. Developing and testing the effectiveness of a novel health qigong for frail elders in Hong Kong: a preliminary study. Evid Based Complement Alternat Med. 2013;2013:827392. 10.1155/2013/827392 PMC3784263 24109493

[112] Voukelatos A, Cumming RG, Lord SR, Rissel C. A Randomized, Controlled Trial of tai chi for the Prevention of Falls: The Central Sydney tai chi Trial. J Am Geriatr Soc. 2007;55(8):1185–91. 10.1111/j.1532-5415.2007.01244.x 17661956

[113] Wolf SL, Barnhart HX, Kutner NG, McNeely E, Coogler C, Xu T. Reducing frailty and falls in older persons: an investigation of Tai Chi and computerized balance training. Atlanta FICSIT Group. Frailty and Injuries: Cooperative Studies of Intervention Techniques. J Am Geriatr Soc. 1996;44(5):489–97. 10.1111/j.1532-5415.1996.tb01432.x 8617895

[114] Wolf SL, O'Grady M, Easley KA, Guo Y, Kressig RW, Kutner M. The influence of intense Tai Chi training on physical performance and hemodynamic outcomes in transitionally frail, older adults. J Gerontol A Biol Sci Med Sci. 2006;61(2):184–9. 10.1093/gerona/61.2.184 16510864

[115] Wolf SL, Sattin RW, Kutner M, O'Grady M, Greenspan AI, Gregor RJ. Intense tai chi exercise training and fall occurrences in older, transitionally frail adults: a randomized, controlled trial. J Am Geriatr Soc. 2003;51(12):1693–701. 10.1046/j.1532-5415.2003.51552.x 14687346

[116] Aibar-Almazán A, Martínez-Amat A, Cruz-Díaz D, De la Torre-Cruz MJ, Jiménez-García JD, Zagalaz-Anula N, et al. Effects of Pilates on fall risk factors in community-dwelling elderly women: A randomized, controlled trial. Eur J Sport Sci. 2019;19(10):1386–94. 10.1080/17461391.2019.1595739 30990762

[117] Greendale GA, Huang MH, Karlamangla AS, Seeger L, Crawford S. Yoga decreases kyphosis in senior women and men with adult-onset hyperkyphosis: results of a randomized controlled trial. J Am Geriatr Soc. 2009;57(9):1569–79. 10.1111/j.1532-5415.2009.02391.x PMC3700806 19682114

[118] Pandya SP (2020). Yoga Education Program for Improving Memory in Older Adults: A Multicity 5-Year Follow-Up Study. J Appl Gerontol.

[119] Doi T, Verghese J, Makizako H, Tsutsumimoto K, Hotta R, Nakakubo S, et al. Effects of Cognitive Leisure Activity on Cognition in Mild Cognitive Impairment: Results of a Randomized Controlled Trial. J Am Med Dir Assoc. 2017;18(8):686–91. 10.1016/j.jamda.2017.02.013 28396179

[120] Hars M, Herrmann FR, Gold G, Rizzoli R, Trombetti A. Effect of music-based multitask training on cognition and mood in older adults. Age Ageing. 2014;43(2):196–200. 10.1093/ageing/aft163 24212920

[121] Lazarou I, Parastatidis T, Tsolaki A, Gkioka M, Karakostas A, Douka S, et al. International Ballroom Dancing Against Neurodegeneration: A Randomized Controlled Trial in Greek Community-Dwelling Elders With Mild Cognitive impairment. Am J Alzheimers Dis Other Demen. 2017;32(8):489–99. 10.1177/1533317517725813 PMC10852896 28840742

[122] Machacova K, Vankova H, Volicer L, Veleta P, Holmerova I. Dance as Prevention of Late Life Functional Decline Among Nursing Home Residents. J Appl Gerontol. 2017;36(12):1453–70. 10.1177/0733464815602111 26320145

[123] Merom D, Mathieu E, Cerin E, Morton RL, Simpson JM, Rissel C, et al. Social Dancing and Incidence of Falls in Older Adults: A Cluster Randomised Controlled Trial. PLoS Med. 2016;13(8):e1002112. 10.1371/journal.pmed.1002112 PMC5004860 27575534

[124] Trombetti A, Hars M, Herrmann FR, Kressig RW, Ferrari S, Rizzoli R (2011). Effect of music-based multitask training on gait, balance, and fall risk in elderly people: a randomized controlled trial. Arch Intern Med..

[125] Shimada H, Lee S, Akishita M, Kozaki K, Iijima K, Nagai K, Ishii S, Tanaka M, Koshiba H, Tanaka T, Toba K (2018). Effects of golf training on cognition in older adults: a randomised controlled trial. J Epidemiol Community Health..

[126] World Health Organization. World report on ageing and health. World Health Organization; 2015.

[127] Gray SM (2021). Physical activity is good for older adults-but is program implementation being overlooked? A systematic review of intervention studies that reported frameworks or measures of implementation. Br J Sports Med.

[128] Sherrington C, et al. Exercise for preventing falls in older people living in the community. Cochrane Database Syst Rev. 2019(1). 10.1002/14651858.CD012424.pub2 PMC6360922 30703272

