# Supplementary material for: Impact of physical activity programs and services for older adults: a rapid review
Source: Int J Behav Nutr Phys Act. 2022 Jul 14;19:87. doi: 10.1186/s12966-022-01318-9 (PMC9284866; doi:10.1186/s12966-022-01318-9)
Supplement: Supplementary file 2 — Additional file 2: Appendix 1. Eligibility criteriaand search strategies used to identify systematic reviews. Appendix 2. Flow chart ofselection of reviews from the updated search. Appendix 3. Systematic reviewsidentified in the updated search. Appendix 4. Search strategies andflow chart of the selection of primary studies investigating sports for older adults. Appendix 5. Eligibility criteriafor selection of primary studies. Appendix 6. Overview of relevant concepts incategorising outcomes of physical activity interventions for older adults. Appendix 7. Overview of primary studies ofphysical activity programs and services for older adults included in Objective2 (k=87 studies). Appendix 8. Description of studiesincluded in Objective 2 according to intervention location. Appendix 9. Dataused to create Figures 2-5 presenting the evidence of physical activityservices and programs for older adults. Appendix 10. Impact of physical activity typeson different outcome domains in adults with physical impairments. Appendix 11. Impact of physicalactivity types on outcome domains in adults with mild cognitive impairment orlow mood. Appendix 12. Impact of physicalactivity on different outcome domains by location. Appendix 13. Impact of structuredexercise in different locations on different outcome domains. Appendix 14. Impact ofrecreation/sport in different locations on different outcome domains. [file 12966_2022_1318_MOESM2_ESM.docx]

Appendix Table of Contents

[APPENDIX 1. Eligibility criteria and search strategies used to identify systematic reviews 3](#_Toc103086575)

[Eligibility criteria for systematic reviews 3](#_Toc103086576)

[Search strategies 4](#_Toc103086577)

[APPENDIX 2. Flow chart of selection of reviews from the updated search 9](#_Toc103086578)

[APPENDIX 3. Systematic reviews identified in the updated search 11](#_Toc103086579)

[APPENDIX 4. Search strategies and flow chart of the selection of primary studies investigating sports for older adults 14](#_Toc103086580)

[Search strategy for primary studies on sports interventions for older adults 14](#_Toc103086581)

[Selection of studies identified in the sports search 20](#_Toc103086582)

[APPENDIX 5. Eligibility criteria for selection of primary studies 20](#_Toc103086583)

[APPENDIX 6. Overview of relevant concepts in categorising outcomes of physical activity interventions for older adults 23](#_Toc103086584)

[International Classification of Functioning, Disability and Health (ICF) 26](#_Toc103086585)

[APPENDIX 7. Overview of primary studies of physical activity programs and services for older adults included in Objective 2 (k=87 studies) 28](#_Toc103086586)

[APPENDIX 8. Description of studies included in Objective 2 according to intervention location 32](#_Toc103086587)

[Table A.8.1. Description of studies of physical activity interventions for older adults conducted in a community facility (k=38 studies) 32](#_Toc103086588)

[Table A.8.2. Description of studies of physical activity interventions for older adults conducted fully at home (k=22 studies) 52](#_Toc103086589)

[Table A.8.3. Description of studies of physical activity interventions for older adults conducted in an outpatient health facility (k=3 studies) 64](#_Toc103086590)

[Table A.8.4. Description of studies of physical activity interventions for older adults conducted in a residential aged care facility (k=8 studies) 67](#_Toc103086591)

[Table A.8.5. Description of studies of physical activity interventions for older adults conducted in a retirement village (k=4 studies) 72](#_Toc103086592)

[Table A.8.6. Description of study of physical activity interventions for older adults conducted in no set location (k=14 studies) 75](#_Toc103086593)

[APPENDIX 9. Data used to create Figures 2-5 presenting the evidence of physical activity services and programs for older adults 83](#_Toc103086594)

[Table A.9.1. Physical activity in all populations and locations by type of activity: impact on different outcome domains (data for Figure 3) 83](#_Toc103086595)

[Table A.9.2. Physical activity in all locations by type of structured exercise: impact on different outcome domains (data for Figure 4) 84](#_Toc103086596)

[Table A.9.3. Physical activity in all populations and locations by type of multicomponent exercise: impact on different outcome domains (data for Figure 5) 85](#_Toc103086597)

[Table A.9.4. Physical activity in all locations by type of recreation/sport: impact on different outcome domains (data for Figure 6) 86](#_Toc103086598)

[APPENDIX 10. Impact of physical activity types on different outcome domains in adults with physical impairments 87](#_Toc103086599)

[Figure A.10. Physical activity in adults with physical impairments/limitations by type of activity: impact on different outcome domains 87](#_Toc103086600)

[Table A.10. Physical activity in people with physical impairments/limitations in all locations by type of activity: impact on different outcome domains (data for Figure A.10) 88](#_Toc103086601)

[APPENDIX 11. Impact of physical activity types on outcome domains in adults with mild cognitive impairment or low mood 89](#_Toc103086602)

[Figure A.11. Physical activity in adults with mild cognitive impairment or low mood by type of activity: impact on different outcome domains 89](#_Toc103086603)

[Table A.11. Physical activity in people with mild cognitive impairments or mood symptoms in all locations by type of activity: impact on different outcome domains (data for Figure A.11) 90](#_Toc103086604)

[APPENDIX 12. Impact of physical activity on different outcome domains by location 91](#_Toc103086605)

[Figure A.12. Physical activity by location: impact of all types of physical activity on different outcome domains 91](#_Toc103086606)

[Table A.12. Physical activity in all populations by location: impact of all types of physical activity on different outcome domains (data for Figure A.12) 92](#_Toc103086607)

[APPENDIX 13. Impact of structured exercise in different locations on different outcome domains 93](#_Toc103086608)

[Figure A.13.1. Physical activity in community facilities by type of structured exercise: impact on different outcome domains 93](#_Toc103086609)

[Table A.13.1. Physical activity in community facilities by type of structured exercise: impact on different outcome domains (data for Figure A.13.1) 94](#_Toc103086610)

[Figure A.13.2. Physical activity in the home by type of structured exercise: impact on different outcome domains 95](#_Toc103086611)

[Table A.13.2. Physical activity in the home by type of structured exercise: impact on different outcome domains (data for Figure A.13.2) 96](#_Toc103086612)

[Figure A.13.3. Physical activity in outpatient health facilities by type of structured exercise: impact on different outcome domains 98](#_Toc103086613)

[Table A.13.3. Physical activity in outpatient health facilities by type of structured exercise: impact on different outcome domains (data for Figure A.13.3) 99](#_Toc103086614)

[Figure A.13.4. Physical activity in residential aged care facilities by type of structured exercise: impact on different outcome domains 100](#_Toc103086615)

[Table A.13.4. Physical activity in residential aged care facilities by type of structured exercise: impact on outcome domains (data for Figure A.13.4) 101](#_Toc103086616)

[Figure A.13.5. Physical activity in retirement villages by type of structured exercise: impact on different outcome domains 102](#_Toc103086617)

[Table A.13.5. Physical activity in retirement villages by type of structured exercise: impact on different outcome domains (data for Figure A.13.5) 103](#_Toc103086618)

[APPENDIX 14. Impact of recreation/sport in different locations on different outcome domains 104](#_Toc103086619)

[Figure A.14.1. Physical activity in community facilities by type of recreation/sport: impact on different outcome domains 104](#_Toc103086620)

[Table A.14.1. Physical activity in community facilities by type of recreation/sport: impact on different outcome domains (data for Figure A.14.1) 105](#_Toc103086621)

[Figure A.14.2. Physical activity in residential aged care facilities by type of recreation/sport: impact on different outcome domains 106](#_Toc103086622)

[Table A.14.2. Physical activity in residential aged care facilities by type of recreation/sport: impact on different outcome domains (data for Figure A.14.2) 107](#_Toc103086623)

[Figure A.14.3. Physical activity in retirement villages by type of recreation/sport: impact on different outcome domains 108](#_Toc103086624)

[Table A.14.3. Physical activity in retirement villages by type of recreation/sport: impact on different outcome (data for Figure A.14.3) 109](#_Toc103086625)

# APPENDIX 1. Eligibility criteria and search strategies used to identify systematic reviews

## Eligibility criteria for systematic reviews

| **Category** | **Inclusion** | **Exclusion** |
| --- | --- | --- |
| Publication language | Full text in English | Abstract-only, other languages |
| Publication date | Published after 1 January 2010 | Published before 1 January 2010 |
| Publication status | Published in peer-reviewed journals | Unpublished reviews |
| Study design | - Systematic review - Meta-analysis | - Narrative/literature review - Commentaries - Editorials - Protocols - Controlled, pre-post trials - Cohort/cross-sectional studies - Reviews of reviews (meta-reviews) - Scoping reviews |
| Characteristics of individual studies | | |
| Population | - Adults 60+ years: healthy; frail; or with a disability. - Reviews including younger participants where average age >=60 years - Bone mineral density conditions - Mild cognitive impairment - Sarcopenia - Obesity - Mixed chronic conditions | - Samples selected for pre-existing medical conditions (e.g. stroke, dementia, Parkinson’s) - Reviews including younger participants where the average age is <60 years |
| Intervention | Question 1 (All PA interventions):   - Physical activity - Complex and/or multifaceted interventions - Health professional education - Community-wide campaigns - Age-friendly environments - Policy   Question 2 (PA programs):   - Supervised/unsupervised - All physical activity types - All PA providers - All program delivery modes | Question 1:  No exclusions where the primary focus is increasing physical activity. Question 2:   - Programs without participant-based PA outcomes. - Perturbation/platform training - Combined interventions (e.g. supplement/nutrition and PA) - Occupational therapy/ physiotherapy without PA |
| Comparators | All included | NA |
| Outcomes | Question 1: Physical activity  Question 2: Longitudinal physical activity, disability, falls, mood, health-related quality of life.  Cognitive function  Strength and fitness  Balance  Laboratory measures of cognitive function  Any outcomes that measure physical activity, participation/social, physical function, cognitive and emotional, global measures (e.g. quality of life, frailty, functioning, wellbeing) | - Disease- or disorder-specific symptoms or laboratory measures. e.g. blood glucose; cholesterol. - Programs without longitudinal outcomes - Adherence-only - Barriers & facilitators only |
| Settings | Recruitment in all settings, but programs delivered in:   - Healthcare settings - Residential care - Community/home settings | Programs delivered in inpatient settings |

## Search strategies

MEDLINE search strategy

| **Description** | **Search terms** |
| --- | --- |
| Limit: language and exclude animal only | (English[lang]) NOT ("Animals"[Mesh] NOT ("Animals"[Mesh] AND "Humans"[Mesh])) |
| Population | AND "old"[tiab] OR "older"[tiab] OR "aged"[tiab] OR "aging"[tiab] OR "ageing"[tiab] OR "elderly” [tiab] |
| Limit: age groups | NOT (("infant"[Mesh] OR "child"[mesh] OR "adolescent"[mh])  NOT (("infant"[Mesh] OR "child"[mesh] OR "adolescent"[mh]) AND "adult"[Mesh])) |
| Limit: date | AND ("2010/01/01"[PDAT]: "3000/12/31"[PDAT]) |
| Publication type | AND (systematic[sb] OR meta-analysis[pt] OR “systematic review” [tiab] OR “systematic literature review” [tiab] OR metaanalysis[tiab] OR "meta analysis"[tiab] OR metanalyses[tiab] OR "meta analyses"[tiab] OR "pooled analysis"[tiab] OR “pooled analyses” [tiab] OR "pooled data"[tiab]) |
| Limit: publication type | NOT (“comment” [Publication Type] OR “editorial” [Publication Type]) |
| Physical activity: | AND (("Exercise"[mh] OR "Exercise"[tiab] OR "Physical activity"[tiab] OR "Lifestyle activities"[tiab] OR "Lifestyle activity"[tiab] OR "Recreational activities"[tiab] OR "Recreational activity"[tiab] OR "Tai ji"[mh] OR "Yoga"[mh] OR "Activities of daily living"[tiab] OR "Activity of daily living"[tiab] OR "Free living activities"[tiab] OR "Free living activity"[tiab] OR "Balance training"[tiab] OR "Qigong"[mh] OR "Functional training"[tiab]) OR (("Aerobic activities"[tiab] OR "Aerobic activity"[tiab] OR "Cardiovascular activities"[tiab] OR "Cardiovascular activity"[tiab] OR "Endurance activities"[tiab] OR "Endurance activity"[tiab] OR "Physical activities"[tiab] OR "Physical conditioning"[tiab] OR "Resistance training"[tiab] OR "strength training"[tiab] OR "Tai chi"[tiab] OR "Tai ji"[tiab] OR "Yoga"[tiab] OR "Walk"[tiab] OR "Walking"[tiab] OR "Chi kung"[tiab] OR "Qigong"[tiab] OR "stretching"[tiab]) NOT medline[sb])) |

CINAHL search strategy

| **Description** | **Search Terms** |
| --- | --- |
| Physical activity | 1. (MH "Exercise+") 2. (TI exercise OR AB exercise) 3. (TI "physical activity" OR AB "physical activity") 4. TI "Lifestyle activities" OR AB "Lifestyle activities" OR TI "Lifestyle activity" OR AB "Lifestyle activity" 5. TI "Recreational activities" OR AB "Recreational activities" OR TI "Recreational activity" OR AB "Recreational activity" 6. (MH "Tai Chi") 7. (MH "Yoga+") 8. (MH "Activities of Daily Living+") 9. TI "Activities of daily living" OR AB "Activities of daily living" 10. TI "Free living activity" OR AB "Free living activity" 11. TI "Balance training" OR AB "Balance training" 12. (MH "Qigong") 13. TI "Functional training" OR AB "Functional training" 14. TI "Aerobic activities" OR AB "Aerobic activities" OR TI "Aerobic activity" OR AB "Aerobic activity" 15. TI "Cardiovascular activities" OR AB "Cardiovascular activities" OR TI "Cardiovascular activity" OR AB "Cardiovascular activity" 16. TI "Endurance activities" OR AB "Endurance activities" OR TI "Endurance activity" OR AB "Endurance activity" 17. TI "Physical activities" OR AB "Physical activities" 18. TI "Physical conditioning" OR AB "Physical conditioning" 19. TI "Resistance training" OR AB "Resistance training" 20. TI "strength training" OR AB "strength training" 21. TI "Tai chi" OR AB "Tai chi" OR TI "Tai ji" OR AB "Tai ji" 22. TI Yoga OR AB Yoga 23. TI Walk OR AB Walk OR TI walking OR AB walking 24. TI "Chi kung" OR AB "Chi kung" OR TI "Qigong" OR AB "Qigong" 25. TI stretching OR AB stretching |
| Combine physical activity | 1. 1-25 OR |
| Limit publication type | 1. (MH "Systematic Review") 2. TI systematic review OR AB systematic review 3. TI systematic literature review OR AB systematic literature review 4. TI metaanalysis OR AB metaanalysis OR TI meta analysis OR AB meta analysis 5. TI metaanalyses OR AB metaanalyses OR TI meta analyses OR AB meta analyses 6. TI meta-analysis OR AB meta-analysis OR TI meta-analyses OR AB meta-analyses 7. TI "pooled analysis" OR AB "pooled analysis" OR TI "pooled analyses" OR AB "pooled analyses" 8. TI "pooled data" OR AB "pooled data" |
| Combine publication type | 1. 27-34 OR |
| Limit age group | 1. TI "old" OR TI "older" OR TI "aged” OR TI "aging" OR TI "ageing" OR TI "elderly” OR AB "old" OR AB "older" OR AB "aged” OR AB "aging" OR AB "ageing" OR AB "elderly” |
| Combine all | 1. 26 AND 35 AND 36 |
| Limits | 1. Limit to English AND Date>01/01/2010 AND exclude MEDLINE |

PEDro search strategy

| **Abstract & Title:** | Physical activity | Exercise |  |  |  |  |
| --- | --- | --- | --- | --- | --- | --- |
| **Therapy:** |  |  | Fitness training | Health promotion | Skill training | Strength training |
| **Subdiscipline:** | Gerontology | Gerontology | Gerontology | Gerontology | Gerontology | Gerontology |
| **Method:** | Systematic review | Systematic review | Systematic review | Systematic review | Systematic review | Systematic review |
| **Published since:** | 2010 | 2010 | 2010 | 2010 | 2010 | 2010 |

Cochrane search strategy

| **Description** | **Search terms** |
| --- | --- |
| Physical activity | 1. MeSH descriptor: [Exercise] explode all trees 2. (exercise): ti, ab, kw 3. (physical activity): ti, ab, kw 4. (Lifestyle activities): ti, ab, kw OR (Lifestyle activity): ti, ab, kw 5. (Recreational activities): ti, ab, kw OR (Recreational activity): ti, ab, kw 6. MeSH descriptor: [Tai Ji] explode all trees 7. MeSH descriptor: [Yoga] explode all trees 8. (Activities of Daily Living): ti, ab, kw 9. MeSH descriptor: [Activities of Daily Living] explode all trees 10. (Free living activity): ti, ab, kw 11. (Balance training): ti, ab, kw 12. MeSH descriptor: [Qigong] explode all trees 13. (Functional training): ti, ab, kw 14. (Aerobic activities): ti, ab, kw OR (Aerobic activity): ti, ab, kw 15. (Cardiovascular activities): ti, ab, kw OR (Cardiovascular activity): ti, ab, kw 16. (Endurance activities): ti, ab, kw OR (Endurance activity): ti, ab, kw 17. (Physical activities): ti, ab, kw 18. (Physical conditioning): ti, ab, kw 19. (Resistance training): ti, ab, kw 20. (strength training): ti, ab, kw 21. (Tai chi): ti, ab, kw OR (Tai Ji): ti, ab, kw 22. (Yoga): ti, ab, kw 23. (Walk): ti, ab, kw OR (Walking): ti, ab, kw 24. (Chi kung): ti, ab, kw OR (Qigong): ti, ab, kw 25. (stretching): ti, ab, kw |
| Combine physical activity | 1. #1 OR #2 OR #3 OR #4 OR #5 OR #6 OR #7 OR #8 OR #9 OR #10 OR #11 OR #12 OR #13 OR #14 OR #15 OR #16 OR #17 OR #18 OR #19 OR #20 OR #21 OR #22 OR #23 OR #24 OR #25 |
| Limit publication type | 1. MeSH descriptor: [Systematic Review] explode all trees 2. (Systematic Review): ti, ab, kw 3. (systematic literature review): ti, ab, kw 4. (metaanalysis): ti, ab, kw OR (meta analysis): ti, ab, kw 5. (metaanalyses): ti, ab, kw OR (meta analyses): ti, ab, kw 6. (meta-analysis): ti, ab, kw OR (meta-analyses): ti, ab, kw 7. (pooled analysis): ti, ab, kw OR (pooled analyses): ti, ab, kw 8. (pooled data): ti, ab, kw |
| Combine publication type | 1. #27 OR #28 OR #29 OR #30 OR #31 OR #32 OR #33 OR #34 |
| Limit age group | 1. (old): ti, ab, kw OR (older): ti, ab, kw OR (aged): ti, ab, kw OR (ageing): ti, ab, kw AND (aging): ti, ab, kw 2. (elderly): ti, ab, kw |
| Combine age group | 1. #26 AND #35 AND (#36 OR #37) |
| Limit date | 1. Limits: with Cochrane Library publication date from Jan 2010 to Nov 2020, in Cochrane Reviews. |

# APPENDIX 2. Flow chart of selection of reviews from the updated search

Full-text studies assessed for eligibility
(n=74)

Eligible reviews identified: (n=33)

Full-text reviews excluded: (n=41)

Ineligible population: (n=16)

Ineligible outcome: (n=4)

Not physical activity intervention: (n=8)

Ineligible study design: (n=10)

Not in English: (n=3)

Excluded by screening of titles and abstract: (n=342)

- PEDro: (n=112)
- PubMed: (n=270)
- CINAHL: (n=148)
- Cochrane Database of Systematic Reviews: (n=58)
- Total after removing duplicates: (n=416)

Duplicate reviews excluded (reviews already included in the scoping review): (n=8)

Additional reviews included: (n=25)

*Reviews included review question 1: (n=3)*

*Reviews included review question 2: (n=23)*

# APPENDIX 3. Systematic reviews identified in the updated search

Table A.3 Systematic reviews of physical activity programs for older adults identified in the updated search classified according to the framework

| **PICO/ TIDieR item** | **PICO aspect of interest, adaption of TIDieR** | **Framework classification  level 1** | **Number of reviews with ALL studies in category^*^**  **k/23 (%)** | **Number of reviews with ANY (1+) studies in category^#^**  **k/23 (%)** |
| --- | --- | --- | --- | --- |
| Population (Older adults) | Country | High income | 2 (9%) | 16 (70%) |
|  |  | Upper middle income | 0 | 11 (48%) |
|  |  | Lower-middle income | 0 | 4 (17%) |
|  |  | Low income | 0 | 0 |
|  |  | Mixed | 21 (91%) | 21 (91%) |
|  | Remoteness | Rural/regional | 0 | 0 |
|  | Age | “Older” old age (85+) | 0 | 0 |
|  | Gender | Male | 0 | 23 (100%) |
|  |  | Female | 0 | 22 (96%) |
|  |  | Any | 23 (100%) | 22 (96%) |
|  | Socio-economic status | Disadvantaged | 0 | 0 |
|  |  | Not specified | 23 (100%) | 23 (100%) |
|  |  | Mixed | 0 | 0 |
|  |  | Cultural or indigenous background | 1 (4%) | 1 (4%) |
|  | Intrinsic capacity | No impairment | 7 (30%) | 14 (61%) |
|  |  | Not specified | 5 (22%) | 5 (22%) |
|  |  | Impaired | 4 (17%) | 11 (48%) |
|  |  | Mixed | 7 (30%) | 7 (30%) |
|  | Particular groups | Care facility residents | 0 | 7 (30%) |
|  |  | Own home/community | 7 (30%) | 15 (65%) |
|  |  | Not specified | 8 (35%) | 8 (35%) |
|  |  | Mixed | 8 (35%) | 8 (35%) |
|  | Health conditions | No chronic health condition | 10 (43%) | 15 (65%) |
|  |  | Chronic health condition | 3 (13%) | 8 (35%) |
|  |  | Mixed | 6 (26%) | 7 (30%) |
|  |  | Not specified | 4 (17%) | 4 (17%) |
| Intervention | Type of program | Physical activity delivered/mixed | 23 (100%) | 23 (100%) |
|  |  | Physical activity promoted | 1 (4%) | 1 (4%) |
|  | Type of physical activity | Overall activity | 0 | 0 |
|  |  | Structured exercise | 10 (43%) | 18 (78%) |
|  |  | Recreation | 5 (22%) | 11 (48%) |
|  |  | Sport | 0 | 1 (4%) |
|  |  | Mixed | 8 (35%) | 7 (30%) |
|  | Provider | Professional | 0 | 5 |
|  |  | Volunteer | 0 | 0 |
|  |  | Carer | 0 | 0 |
|  |  | None | 2 (9%) | 6 (26%) |
|  |  | Mixed | 5 (22%) | 4 (17%) |
|  |  | Not specified | 16 (69%) | 16 (69%) |
|  | Who with | Individual | 2 (9%) | 19 (83%) |
|  |  | Group | 3 (13%) | 21 (91%) |
|  |  | Mixed | 18 (78%) | 16 (69%) |
|  | Delivery mode | Synchronous/ live | 15 (65%) | 21 (91%) |
|  |  | Asynchronous/ pre-recorded | 0 | 9 (39%) |
|  |  | Mixed | 8 (35%) | 8 (35%) |
|  | Location | Health service | 1 (4%) | 4 (17%) |
|  |  | Workplace | 0 | 0 |
|  |  | Community facility | 5 (22%) | 19 (83%) |
|  |  | Residential care | 00 | 3 (13%) |
|  |  | Assisted living | 0 | 4 (17%) |
|  |  | Own home | 2 (9%) | 10 (43%) |
|  |  | Faith-based | 0 | 1 (4%) |
|  |  | Park/ sports field | 0 | 1 (4%) |
|  |  | Mixed | 15 (65%) | 14 (61%) |
| Comparison | No intervention | No intervention | 3 (13%) | 22 (96%) |
|  | Physical activity | Higher dose of same activity | 1 (4%) | 2 (9%) |
|  |  | Different physical activity | 0 | 13 (56%) |
|  | Other intervention  Mixed | Education | 0 | 8 (35%) |
|  |  | Other | 0 | 15 (65%) |
|  |  | Mixed | 19 (83%) | 19 (83% |
| Outcome | Physical activity | Self-report | 0 | 1 (4%) |
|  |  | Observation | 0 | 1 (4%) |
|  |  | Device-based | 0 | 1 (4%) |
|  |  | Mixed | 1 (4%) | 1 (4%) |
|  |  | None | 22 (96%) | 22 (96%) |
|  | Social functioning (participation) | Self-report | 0 | 0 |
|  |  | Observation | 0 | 0 |
|  |  | Device-based | 0 | 0 |
|  |  | Mixed | 0 | 0 |
|  |  | None | 23 (100%) | 23 (100%) |
|  | Physical functioning | Self-report | 0 | 1 (4%) |
|  |  | Observation | 4 (17%) | 9 (39%) |
|  |  | Device-based | 4 (17%) | 9 (39%) |
|  |  | Mixed | 6 (26%) | 6 (26%) |
|  |  | None | 9 (39%) | 9 (39%) |
|  | Cognitive and emotional functioning | Self-report | 0 | 1 (4%) |
|  |  | Observation | 10 (43%) | 12 (52%) |
|  |  | Device-based | 0 | 2 (9%) |
|  |  | Mixed | 2 (9%) | 2 (9%) |
|  |  | None | 11 (48%) | 11 (48%) |
|  | Well-being, quality of life, composite measures of functioning | Self-report | 0 | 2 (9%) |
|  |  | Observation | 1 (4%) | 1 (4%) |
|  |  | Mixed | 1 (4%) | 1 (4%) |
|  |  | None | 21 (91%) | 21 (91%) |

*This column indicates reviews with ALL included studies in this category. That is, the subcategories are mutually exclusive and the total of categories equals the 23 included reviews.

#This column shows reviews that included ANY studies meeting this category definitions. That is, the subcategories are not mutually exclusive and one review may be reported in multiple subcategories.

# APPENDIX 4. Search strategies and flow chart of the selection of primary studies investigating sports for older adults

## Search strategy for primary studies on sports interventions for older adults

**Medline (Ovid)**

1. Baseball/ or Basketball/ or Bicycling/ or Boxing/ or Football/ or Golf/ or Gymnastics/ or Hockey/ or exp Martial Arts/ or exp Racquet Sports/ or cricket sport/ or team sports/
2. Return to Sport/ or exp Running/ or Skating/ or exp Snow Sports/ or Soccer/ or exp Swimming/
3. Volleyball/ or exp Water Sports/ or Weight Lifting/ or Wrestling/
4. "Track and Field"/
5. (mountain bik* or sports or AFL or alpine ski* or archery or athletics or badminton or basketball or biathlon or biking or Boxing or canoe* or cricket or cross country ski* or curling).tw.
6. (cycling or diving or duathlon or equestrian or fencing or football or golf or gymnastics or Handball or hippotherapy or Hockey or horseback riding or horse riding or judo or kayak or kickboxing or lawn bowls or bowling).tw.
7. (marathon or netball or badminton or snowboard or triathlon or Polo or powerlifting or runn* or rowing or sailing or shooting or Skating or skiing or snowboard or soccer or sport*).tw.
8. (surfing or swimming or table tennis or taekwondo or Tae Kwon Do or tenpin bowling or Tennis or Trampolin* or triathlon or volleyball or volley).tw.
9. (australian football or baseball or fencing or racing or rugby or sport* or tennis or union or league).tw.
10. 1 or 2 or 3 or 4 or 5 or 6 or 7 or 8 or 9
11. *Aged/ or *"Aged, 80 and over"/
12. exp Aged/ not Adolescent.mp.
13. (elderly or seniors or geriatric).tw.
14. (older adj (adult or people or person$1)).tw.
15. 11 or 12 or 13 or 14
16. Randomized Controlled Trials as Topic/
17. Random Allocation/
18. Controlled Clinical Trials as Topic/
19. Control Groups/
20. Double-Blind Method/
21. Single-Blind Method/
22. Placebos/
23. randomized controlled trial.pt.
24. controlled clinical trial.pt.
25. (random$ or RCT or RCTs).tw.
26. (controlled adj5 (trial$ or stud$)).tw.
27. (clinical$ adj5 trial$).tw.
28. (randomi?ed adj5 trial).mp.
29. 16 or 17 or 18 or 19 or 20 or 21 or 22 or 23 or 24 or 25 or 26 or 27 or 28
30. exp Animals/ not Humans/
31. 29 not 30
32. 10 and 15 and 31

**Results: 3391 on 19 April 2021**

**CINAHL (Ebsco)**

S1 (MH "Baseball" OR "Basketball" OR "Cycling" OR "Boxing" OR "Football" OR "Golf" OR "Gymnastics" OR "Hockey" OR "Martial Arts" OR "Racquet Sports+" OR "Sports Re-Entry" OR "Running+" OR "Skating+" OR "Winter Sports+" OR "Soccer" OR "Sports" OR "Swimming" OR "Track and Field" OR "Volleyball" OR "Aquatic Sports+" OR "Weight Lifting" OR "Wrestling" OR "Sports Participation" OR "Bowling" OR "Fencing" OR "Handball" OR "Race Walking" OR "Skiing+" OR "Team Sports+" OR "Triathlon")

S2 (TI "mountain bik*" OR "sports" OR AFL OR "alpine ski*" OR archery OR athletics OR badminton OR basketball OR biathlon OR biking OR Boxing OR canoe* OR cricket OR "cross#country ski*" OR curling OR cycling OR diving OR duathlon OR equestrian OR fencing OR football)

S3 (AB "mountain bik*" OR "sports" OR AFL OR "alpine ski*" OR archery OR athletics OR badminton OR basketball OR biathlon OR biking OR Boxing OR canoe* OR cricket OR "cross#country ski*" OR curling OR cycling OR diving OR duathlon OR equestrian OR fencing OR football)

S4 (TI golf OR gymnastics OR Handball OR hippotherapy OR Hockey OR "horseback riding" OR "horse riding" OR judo OR kayak OR kickboxing OR "lawn bowls" OR bowling OR marathon OR netball OR badminton OR snowboard OR Triathlon OR Polo OR powerlifting OR runn* OR rowing OR sailing)

S5 (AB golf OR gymnastics OR Handball OR hippotherapy OR Hockey OR "horseback riding" OR "horse riding" OR judo OR kayak OR kickboxing OR "lawn bowls" OR bowling OR marathon OR netball OR badminton OR snowboard OR Triathlon OR Polo OR powerlifting OR runn* OR rowing OR sailing)

S6 (TI shooting OR Skating OR skiing OR snowboard OR soccer OR sport* OR surfing OR swimming OR "table tennis" OR taekwondo OR "Tae Kwon Do" OR "tenpin bowling" OR Tennis OR Trampolin* OR triathlon OR volleyball OR volley)

S7 (AB shooting OR Skating OR skiing OR snowboard OR soccer OR sport* OR surfing OR swimming OR "table tennis" OR taekwondo OR "Tae Kwon Do" OR "tenpin bowling" OR Tennis OR Trampolin* OR triathlon OR volleyball OR volley)

S8 (TI "australian football" OR "baseball" OR " curling" OR "fencing" OR " racing" OR "rugby" OR " sport*" OR "winter sports" OR Wrestling)

S9 (AB "australian football" OR "baseball" OR " curling" OR "fencing" OR " racing" OR "rugby" OR " sport*" OR "winter sports" OR Wrestling)

S10 S1 OR S2 OR S3 OR S4 OR S5 OR S6 OR S7 OR S8 OR S9

S11 (MH "Randomized Controlled Trials") OR (MH "Clinical Trials+") OR "Randomized Controlled Trial"

S12 (MH "Aged+") OR (MH "Aged, 80 and Over")

S13 S10 AND S11 AN S12

**Results: 1012 on 19 April 2021**

**SPORTDiscus (ebsco)**

S1 DE "BASEBALL" OR DE "BASKETBALL" OR DE "CYCLING" OR DE "BOXING" OR DE "FOOTBALL" OR DE "GOLF" OR DE "GYMNASTICS" OR DE "HOCKEY" OR DE "MARTIAL Arts" OR DE "RACQUETBALL" OR DE "SPORT for all" OR DE "RUNNING" OR DE "SKATING" OR DE "WINTER sports" OR DE "SOCCER"

S2 DE " sports" OR DE "MOTORSOCCER" OR DE "RUGBALL" OR DE " bowling" OR DE " dance sport" OR DE " fencing" OR DE " hockey" OR DE "road racing" OR DE "rugby" OR DE " soccer" OR DE " sports competitions" OR DE " tennis" OR DE " track & field" OR DE " workouts"

S3 DE "SWIMMING" OR DE "VOLLEYBALL" OR DE "AQUATIC sports" OR DE "AQUATIC sports competitions" OR DE "CANOE polo" OR DE "CANOES & canoeing" OR DE "DIVING" OR DE "DRAGON boat racing" OR DE "FISHING" OR DE "KNEEBOARDING" OR DE "RAFTING (Sports)" OR DE "REGATTAS" OR DE "ROWING" OR DE "SAILBOAT racing" OR DE "SAILING" OR DE "SURFING" OR DE "WATER polo" OR DE "WATER skiing" OR DE "WHITEWATER kayaking" OR DE "WHITEWATER rafting"

S4 DE "WEIGHT lifting" OR DE "WEIGHT lifting competitions" OR DE "WRESTLING" OR DE "SPORTS participation" OR DE "BOWLING" OR DE "FENCING" OR DE "HANDBALL" OR DE "WALKING (Sports)" OR DE "SKIS & skiing" OR DE "TEAM sports" OR DE "TRIATHLON"

S5 TI (mountain bik* OR sports OR AFL OR alpine ski* OR archery OR athletics OR badminton OR basketball OR biathlon OR biking OR Boxing OR canoe* OR cricket OR cross#country ski* OR curling OR cycling OR diving OR duathlon OR equestrian OR fencing OR football)

S6 TI (golf OR gymnastics OR Handball OR hippotherapy OR Hockey OR horseback riding OR horse riding OR judo OR kayak OR kickboxing OR lawn bowls OR bowling OR marathon OR netball OR snowboard OR triathlon OR Polo OR powerlifting OR runn* OR rowing OR sailing)

S7 TI (shooting OR Skating OR skiing OR snowboard OR soccer OR sport* OR surfing OR swimming OR table tennis OR taekwondo OR Tae Kwon Do OR tenpin bowling OR Tennis OR Trampolin* OR triathlon OR volleyball OR volley)

S8 TI (aussie rules OR australian football OR baseball OR fencing OR racing OR rugby OR winter sports OR Wrestling)

S9 AB (mountain bik* OR sports OR AFL OR alpine ski* OR archery OR athletics OR badminton OR basketball OR biathlon OR biking OR Boxing OR canoe* OR cricket OR cross#country ski* OR curling OR cycling OR diving OR duathlon OR equestrian OR fencing OR football)

S10 AB (golf OR gymnastics OR Handball OR hippotherapy OR Hockey OR horseback riding OR horse riding OR judo OR kayak OR kickboxing OR lawn bowls OR bowling OR marathon OR netball OR snowboard OR triathlon OR Polo OR powerlifting OR runn* OR rowing OR sailing)

S11 AB (shooting OR Skating OR skiing OR snowboard OR soccer OR sport* OR surfing OR swimming OR table tennis OR taekwondo OR Tae Kwon Do OR tenpin bowling OR Tennis OR Trampolin* OR triathlon OR volleyball OR volley)

S12 AB (aussie rules OR australian football OR baseball OR fencing OR racing OR rugby OR winter sports OR Wrestling)

S13 S1 OR S2 OR S3 OR S4 OR S5 OR S6 OR S7 OR S8 OR S9 OR S10 OR S11 OR S12

S14 controlled trial OR clinical trial

S15 random*

S16 random sampling

S17 clinic* W5 trial*

S18 random allocation

S19 randomized OR randomised

S20 randomized controlled trials

S21 S14 OR S15 OR S16 OR S17 OR S18 OR S19 OR S20

S22 Older person OR older people OR older adult*

S23 "elderly" or "senior"

S24 DE "OLDER people" OR DE "AGING" OR DE "GERIATRICS" OR DE "RETIREMENT"

S25 S22 OR S23 OR S24

S26 S13 AND S21 AND S25

**Results: 365 on 19 April 2021**

**PEDro**

Sport (AB TI) AND gerontology (Subdiscipline) AND Clinical trial (method)

**Results: 24**

Skiing (AB TI) AND gerontology (Subdiscipline) AND Clinical trial (method)

**Results: 23**

archery (AB TI) AND gerontology (Subdiscipline) AND Clinical trial (method)

**Results: 0**

athletics (AB TI) AND gerontology (Subdiscipline) AND Clinical trial (method)

**Results: 0**

badminton (AB TI) AND gerontology (Subdiscipline) AND Clinical trial (method)

**Results: 0**

basketball (AB TI) AND gerontology (Subdiscipline) AND Clinical trial (method)

**Results: 1**

biathlon* (AB TI) AND gerontology (Subdiscipline) AND Clinical trial (method)

**Results: 0**

biking (AB TI) AND gerontology (Subdiscipline) AND Clinical trial (method)

**Results: 0**

cycling (AB TI) AND gerontology (Subdiscipline) AND Clinical trial (method)

**Results: 54**

boxing (AB TI) AND gerontology (Subdiscipline) AND Clinical trial (method)

**Results: 7**

canoe* (AB TI) AND gerontology (Subdiscipline) AND Clinical trial (method)

**Results: 1**

cricket (AB TI) AND gerontology (Subdiscipline) AND Clinical trial (method)

**Results: 0**

curling (AB TI) AND gerontology (Subdiscipline) AND Clinical trial (method)

**Results: 1**

Diving (AB TI) AND gerontology (Subdiscipline) AND Clinical trial (method)

**Results: 0**

duathlon* (AB TI) AND gerontology (Subdiscipline) AND Clinical trial (method)

**Results: 0**

equestrian (AB TI) AND gerontology (Subdiscipline) AND Clinical trial (method)

**Results: 0**

fencing (AB TI) AND gerontology (Subdiscipline) AND Clinical trial (method)

**Results: 0**

football (AB TI) AND gerontology (Subdiscipline) AND Clinical trial (method)

**Results: 3**

golf (AB TI) AND gerontology (Subdiscipline) AND Clinical trial (method)

**Results: 3**

gymnastics (AB TI) AND gerontology (Subdiscipline) AND Clinical trial (method)

**Results: 16**

handball (AB TI) AND gerontology (Subdiscipline) AND Clinical trial (method)

**Results: 1**

hockey (AB TI) AND gerontology (Subdiscipline) AND Clinical trial (method)

**Results: 0**

horseback riding (AB TI) AND gerontology (Subdiscipline) AND Clinical trial (method)

**Results: 1**

horse riding (AB TI) AND gerontology (Subdiscipline) AND Clinical trial (method)

**Results: 3**

judo (AB TI) AND gerontology (Subdiscipline) AND Clinical trial (method)

**Results: 0**

kayak (AB TI) AND gerontology (Subdiscipline) AND Clinical trial (method)

**Results: 3**

kickboxing (AB TI) AND gerontology (Subdiscipline) AND Clinical trial (method)

**Results: 0**

lawn bowls (AB TI) AND gerontology (Subdiscipline) AND Clinical trial (method)

**Results: 0**

marathon (AB TI) AND gerontology (Subdiscipline) AND Clinical trial (method)

**Results:0**

netball (AB TI) AND gerontology (Subdiscipline) AND Clinical trial (method)

**Results:0**

polo (AB TI) AND gerontology (Subdiscipline) AND Clinical trial (method)

**Results: 0**

power lifting (AB TI) AND gerontology (Subdiscipline) AND Clinical trial (method)

**Results: 5**

run* (AB TI) AND gerontology (Subdiscipline) AND Clinical trial (method)

**Results: 39**

rowing (AB TI) AND gerontology (Subdiscipline) AND Clinical trial (method)

**Results: 3**

sailing (AB TI) AND gerontology (Subdiscipline) AND Clinical trial (method)

**Results: 0**

shooting (AB TI) AND gerontology (Subdiscipline) AND Clinical trial (method)

**Results: 0**

skating (AB TI) AND gerontology (Subdiscipline) AND Clinical trial (method)

**Results: 0**

snowboard (AB TI) AND gerontology (Subdiscipline) AND Clinical trial (method)

**Results: 0**

soccer (AB TI) AND gerontology (Subdiscipline) AND Clinical trial (method)

**Results: 3**

surfing (AB TI) AND gerontology (Subdiscipline) AND Clinical trial (method)

**Results: 0**

swimming (AB TI) AND gerontology (Subdiscipline) AND Clinical trial (method)

**Results: 14**

table tennis (AB TI) AND gerontology (Subdiscipline) AND Clinical trial (method)

**Results: 1**

Taekwondo (AB TI) AND gerontology (Subdiscipline) AND Clinical trial (method)

**Results: 0**

Tae Kwon Do (AB TI) AND gerontology (Subdiscipline) AND Clinical trial (method)

**Results: 0**

tenpin bowling (AB TI) AND gerontology (Subdiscipline) AND Clinical trial (method)

**Results: 0**

tennis (AB TI) AND gerontology (Subdiscipline) AND Clinical trial (method)

**Results: 2**

trampoline (AB TI) AND gerontology (Subdiscipline) AND Clinical trial (method)

**Results: 3**

triathlon* (AB TI) AND gerontology (Subdiscipline) AND Clinical trial (method)

**Results: 0**

volleyball (AB TI) AND gerontology (Subdiscipline) AND Clinical trial (method)

## Selection of studies identified in the sports search

Total number of primary study records: **(n=4,959)**

- CINAHL: 1012
- Medline: 3391
- PEDro: 191
- SPORTSDiscuss: 365
- Total: 4,959

Total after removing duplicates: 4,267

- Studies identified for full text review (n=22)

Eligible primary studies included in *Component 1* **(n=14)**

Full-text studies excluded: (n=8)

Reasons for exclusion:

- Ineligible population (disease groups): (n=7)
- Ineligible intervention: (n=1)

Studies excluded (n=13)

- Study design: non-RCT: (n=1)
- Sample size <100 (n=13)
- Comparison group received some active intervention (n=3)

Eligible records included in *Component 2* (n=1) *

Studies excluded by title screening (n=4,246)

*This study was also identified via our screening of systematic reviews included in the scoping review.

# APPENDIX 5. Eligibility criteria for selection of primary studies

| **Category** | **Inclusion** | **Exclusion** |
| --- | --- | --- |
| Publication language | Full text in English | Abstract-only, other languages |
| Publication date | No limits | N/A |
| Publication status | Published in peer-reviewed journals | Unpublished studies |
| Study design | Evaluative studies (randomised-controlled trials, quasi-randomised trials, before and after studies) | - Cross-sectional studies - Case studies - Longitudinal exploratory studies (i.e. not investigating the effect of an intervention, but instead the association between variables) |
| **Characteristics of individual studies** | | |
| Population | - Adults 60+ years not selected on the basis of pre-existing conditions. Included studies may investigate samples of older adults who are: healthy; frail; with a disability, bone mineral density conditions, mild cognitive impairment, sarcopenia, obesity, mixed chronic conditions (e.g. diabetes, hypertension), or others. - Studies including younger participants where average age >=60 years | - Samples selected for pre-existing medical conditions (e.g. stroke, dementia, Parkinson’s, osteoporosis, hip fracture, depression, visual impairment osteoarthritis) - Studies including younger participants where the average age is <60 years |
| Intervention | Physical activity programs or services:   - “Whole body” Physical activity promoted or delivered - Supervised/unsupervised - All physical activity types - All physical activity providers - All program delivery modes | - Whole-body vibration /platform training - Combined interventions (e.g. supplement/nutrition and physical activity) - Occupational therapy/ physiotherapy without a clear physical activity component - Passive interventions, such as stretching. |
| Comparators | All included | NA |
| Outcomes | Any outcomes that measure physical activity, falls, physical function, participation/social, cognitive/emotional (mood) function, global measures (e.g. quality of life, frailty, functioning, wellbeing).  Physical function includes strength, fitness, balance, BMI, BMD, Cholesterol level, glucose level, | - Laboratory measures with no direct clinical use (inflammatory markers, neurotrophic biomarkers, lumbar spine kinematics, proprioception) or that are not routinely collected in clinical practice (e.g. Laboratory measures of cognitive function such as brain volume) - Joint-specific measures (e.g. Proprioception, ROM) - Adherence-only - Barriers & facilitators only - Fear of falling |
| Settings | Recruitment in all settings, but programs delivered in:   - Healthcare settings - Residential care - Community/home settings - Retirement villages | Programs delivered in inpatient settings |

BMI: body mass index, BMD: bone mineral density, ROM: range of motio

# APPENDIX 6. Overview of relevant concepts in categorising outcomes of physical activity interventions for older adults

There are different ways of categorising outcomes relating to functioning and disability among for older adults. Our framework (Appendix 6) is primarily informed by two other WHO initiatives: the *World Report on Ageing and Health^[[1]](#footnote-2)^* and the *International Classification of Functioning, Disability and Health (ICF)*.^[[2]](#footnote-3)^ Key concepts from these initiatives are overviewed here.

***The World Report on Ageing and Health*** provides the following useful definitions of terms relevant to healthy ageing and physical activity. Physical activity across the life course could theoretically optimise intrinsic capacity and increase resilience, thus extending periods of functional ability and well-being.

- ***Healthy ageing*** *is the process of developing and maintaining the functional ability that enables well-being in older age.*
- ***Functional ability*** *comprises the health-related attributes that enable people to be and to do what they have reason to value. It is made up of the intrinsic capacity of the individual, relevant environmental characteristics and the interactions between the individual and these characteristics.*
- ***Intrinsic capacity*** *is the composite of all the physical and mental capacities of an individual.*
- ***Environments*** *comprise all the factors in the extrinsic world that form the context of an individual’s life. These include – from the micro- level to the macro-level – home, communities and the broader society. Within these environments are a range of factors, including the built environment, people and their relationships, attitudes and values, health and social policies, the systems that support them, and the services that they implement.*
- ***Well-being*** *is considered in the broadest sense and includes domains such as happiness, satisfaction and fulfilment.*
- ***Resilience*** *is the ability to maintain or improve a level of functional ability in the face of adversity. Reserves of functional ability contribute to resilience.*

According to *World Report on Ageing and Health^[[3]](#footnote-4)^* healthy ageing starts at birth with genetic inheritance. The expression of these genes can be influenced by experiences in the womb, and by subsequent environmental exposures and behaviours. But each of us is also born into a social milieu. Personal characteristics include those that are usually fixed, such as our sex and ethnicity, as well as those that have some mobility or reflect social norms, such as our occupation, educational attainment, gender or wealth. As people age, they experience a gradual accumulation of molecular and cellular damage that results in a general decrease in physiological reserves. These broad physiological and homeostatic changes are largely inevitable, although their extent will vary significantly among individuals at any particular chronological age. On top of these underlying changes, exposures to a range of positive and negative environmental influences across the life course can influence the development of other health characteristics, such as physiological risk factors (for example, high blood pressure), diseases, injuries and broader geriatric syndromes. The interaction among these health characteristics will ultimately determine the intrinsic capacity of the individual. This means that impacts of age-related changes on intrinsic capacity are not linear. They are highly individualised and associated with lifetime health behaviours, resulting in diverse capacity within older age groups. As the prevalence of chronic conditions and disabilities is greater in older than younger adults it is important to consider functional ability when catering for older adults but equally important not to assume that all older adults will have impaired intrinsic capacity or functional ability.

Several figures from *World Report on Ageing and Health* highlight the interaction between these terms and the potential role of physical activity in healthy ageing. We have reproduced these Figures below from the *World Report on Ageing and Health* overviews the lifelong contributors to healthy ageing. Physical activity is a key health-related behaviour that contributes importantly to an individual’s health characteristics which will then improve their intrinsic capacity, as shown in Figure A.6.1 below. Figure A.6.2 shows the potential for interventions such as physical activity to alter trajectories of physical capacity with ageing. Physical activity over the lifespan has the potential to enable physical capacity trajectories to be closer to path A (intrinsic capacity remains high throughout life) than path C (gradual decline in intrinsic capacity with ageing). The dotted lines show the potential for physical activity-based rehabilitation interventions to alter trajectories. Such interventions have the potential to alter the gradual decline seen in path C as well as helping an individual to recover after an acute event as shown in path B.

*Figure A.6.1 Contributors to healthy ageing*


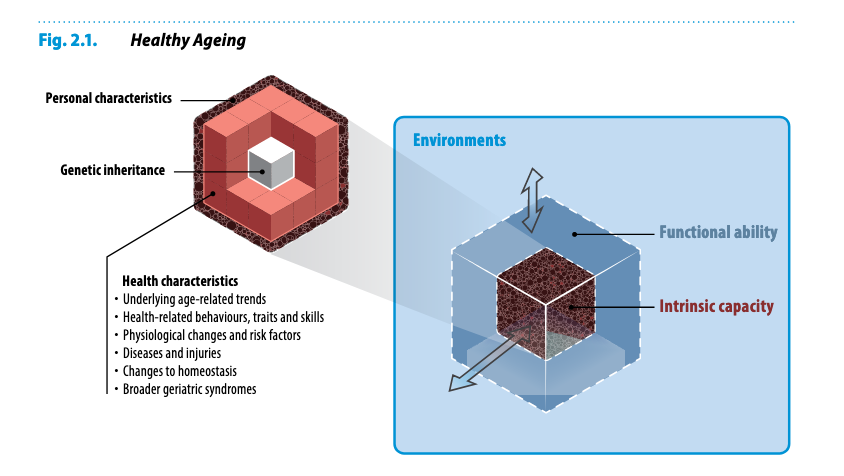


*Figure A**.6.2. Three hypothetical trajectories of physical capacity*


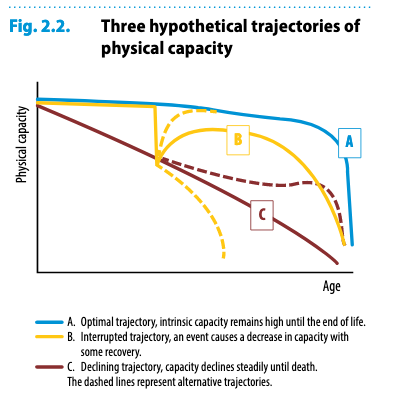


*Figures from the World Report on Ageing and Health^[[4]](#footnote-5)^, see text above for explanation of terms and the role of physical activity.*

## International Classification of Functioning, Disability and Health (ICF)

The ICF^[[5]](#footnote-6)^ provides a useful framework for categorising the impacts of physical activity interventions. The ICF uses a *standard language and framework for the description of health and health-related states. It is a classification of health and health-related domains - domains that help us to describe changes in body function and structure, what a person with a health condition can do in a standard environment (their level of capacity), as well as what they actually do in their usual environment (their level of performance). These domains are classified from body, individual and societal perspectives by means of two lists: a list of body functions and structure, and a list of domains of activity and participation. In ICF, the term functioning refers to all body functions, activities and participation, while disability is similarly an umbrella term for impairments, activity limitations and participation restrictions. ICF also lists environmental and personal factors that interact with all these components.*

The ICF acknowledges that every human being can experience a decrement in health and thereby experience some disability. Physical activity has the potential to impact on body structure and function, activity, participation as described by the ICF.

The ICF provides a method to categorise outcomes of physical activity interventions and has been used in the classification described in the following chapter.

# APPENDIX 7. Overview of primary studies of physical activity programs and services for older adults included in Objective 2 (k=87 studies)

| **PICO/ TIDieR item** | **PICO aspect of interest, adaption of TIDieR** | **Framework classification** | | | **Number of studies in each category**  **k/87 (%)** | |
| --- | --- | --- | --- | --- | --- | --- |
| Population (Older adults) | Country | High-income | | | 81 (93%) | |
|  |  | Upper-middle income | | | 5 (6%) | |
|  |  | Lower-middle income | | | 0 | |
|  |  | Low income | | | 0 | |
|  |  | Mixed | | | 1 (1%) | |
|  | Remoteness | Rural/remote | | | 0 | |
|  |  | Urban | | | 13 (15%) | |
|  |  | Both | | | 1 (1%) | |
|  |  | Not-specified | | | 73 (84%) | |
|  | Age | Middle age (≥50 years) | | | 7 (8%) | |
|  |  | ≥60 years | | | 24 (28%) | |
|  |  | ≥65 years | | | 32 (37%) | |
|  |  | ≥70 | | | 17 (20%) | |
|  |  | ≥75 years | | | 3 (3%) | |
|  |  | ≥80 years | | | 3 (3%) | |
|  |  | ≥85 years | | | 1 (1%) | |
|  |  | ≥90 years | | | 0 | |
|  | Gender | Any | | | 76 (87%) | |
|  |  | Female | | | 8 (10%) | |
|  |  | Male | | | 3 (3%) | |
|  | Cultural /socio-economic background | Cultural | | | 0 | |
|  |  | Linguistic | | | 0 | |
|  |  | Migrant | | | 0 | |
|  |  | Indigenous | | | 0 | |
|  |  | Socio-economic | | | 0 | |
|  |  | No | | | 87 (100%) | |
|  | Living arrangements | Own home | | | 32 (37%) | |
|  |  | Public housing complex | | | 1 (1%) | |
|  |  | Retirement village | | | 4 (5%) | |
|  |  | Residential aged care facility | | | 9 (10 %) | |
|  |  | Not an eligibility criterion | | | 41 (47%) | |
|  | Physical activity level | Yes | | | 26 (30%) | |
|  |  | No | | | 61 (60%) | |
|  | Physical impairment/limitation | Mobility | | | 12 (14%) | |
|  |  | Frailty | | | 6 (7%) | |
|  |  | Osteopenia | | | 0 | |
|  |  | Sarcopenia | | | 0 | |
|  |  | Fall risk | | | 10 (11%) | |
|  |  | Nutrition | | | 0 | |
|  |  | Vision | | | 0 | |
|  |  | Hearing | | | 0 | |
|  |  | Pain | | | 0 | |
|  |  | Not an eligibility criterion | | | 59 (68%) | |
|  | Cognitive impairment or low mood | Cognitive impairment | Mild cognitive impairment | | 9 (10%) | |
|  |  | Low mood | Symptoms of depression | | 1 (1%) | |
|  |  |  | Symptoms of anxiety | | 0 | |
|  |  | Not an eligibility criterion | | | 77 (89%) | |
|  | Health conditions | Mixed chronic conditions | | | 3 (3%) | |
|  |  | Not an eligibility criterion | | | 84 (97%) | |
| Intervention* | Type of program | Physical activity delivered | | | 84 (79%) | |
|  |  | Physical activity promoted | Coaching | | 13 (12%) | |
|  |  |  | Referral | | 0 | |
|  |  |  | Brief/very brief intervention | | 3 (3%) | |
|  |  |  | eHealth/mHealth | | 1 (1%) | |
|  |  |  | Other | | 5 (5%) | |
|  | Type of physical activity | Overall activity | | | 21 (20%) | |
|  |  | Structured exercise | Balance, functional, neuromotor | | 3 (3%) | |
|  |  |  | Strength, resistance, power | | 14 (13%) | |
|  |  |  | Walking/wheeling | | 1 (1%) | |
|  |  |  | Endurance | | 3 (3%) | |
|  |  |  | High-intensity interval training | | 0 | |
|  |  |  | Multicomponent | | 38 (36%) | |
|  |  | Recreation | Tai Chi | | 16 (15%) | |
|  |  |  | Yoga/Pilates | | 3 (3%) | |
|  |  |  | Dance | | 5 (4%) | |
|  |  |  | Non-competitive sport | | 0 | |
|  |  |  | Other | | 0 | |
|  |  | Competitive sport | Standard | | 1 (1%) | |
|  |  |  | Modified | | 0 | |
|  |  | Exercise-based videogame | | | 1 (1%) | |
|  | Prescribed dose | Interventions | Supervised | | 97 (89%) | |
|  |  |  | Unsupervised | | 50 (46%) | |
|  |  | Session duration  *(minutes/session)* | Supervised ^ | | n=97  (91%) | Mean 49.2  SD (18.7) |
|  |  |  | Unsupervised ^ | | n=50  (47%) | Mean 38.7  SD (16.1) |
|  |  | Session frequency  *(time/week)* | Supervised^ | | n=95  (89%) | Mean 1.8  SD (1.2) |
|  |  |  | Unsupervised ^ | | n=50  (47%) | Mean 4.4  SD (2.2) |
|  |  | Program duration  *(weeks)* | Supervised ^ | | n=96  (90%) | Mean 36.6  SD (34.8) |
|  |  |  | Unsupervised^ | | n=50  (47%) | Mean 42.7  SD (34.1) |
|  | Type of supervision | In-person | | | 77 (72%) | |
|  |  | Online | | | 3 (3%) | |
|  |  | Not applicable | | | 22 (21%) | |
|  |  | Both | | | 4 (4%) | |
|  | Additional strategies to support physical activity^@^ | Behaviour change | Activity monitor | | 14 (13%) | |
|  |  |  | Mentoring | | 13 (12%) | |
|  |  |  | Social activity | | 6 (5%) | |
|  |  |  | Incentives | | 8 (7%) | |
|  |  |  | Booklet | | 9 (8%) | |
|  |  |  | Other | | 32 (30%) | |
|  |  | No additional strategies used | | | 58 (54%) | |
|  | Number of additional strategies to support physical activity per study | 1 | | | 21 (20%) | |
|  |  | 2 | | | 21 (20%) | |
|  |  | 3 | | | 7 (7%) | |
|  | Who prescribed | Professional | Health professional | | 61 (57%) | |
|  |  |  | Physical activity leader | | 45 (43%) | |
|  |  | Volunteer | No age restriction volunteer | | 0 | |
|  |  |  | Peer volunteer | | 0 | |
|  |  | Carer/caregiver | Professional carer/caregiver | | 0 | |
|  |  |  | Family/peer-support | | 0 | |
|  | Who delivered (if different to who prescribed) | Delivered by different personnel | | | 43 (40%) | |
|  |  | Delivered by same personnel | | | 40 (38%) | |
|  |  | Not applicable (e.g., home exercise program) | | | 23 (22%) | |
|  | Who with | Individual | | | 43 (41%) | |
|  |  | One other person | | | 2 (2%) | |
|  |  | Group | | | 47 (44%) | |
|  |  | Combination | | | 14 (13%) | |
|  | Delivery mode | Synchronous/ live | | In person | 81 (77%) | |
|  |  |  |  | Video | 0 | |
|  |  |  |  | Phone | 6 (6%) | |
|  |  |  |  | App/Web-based | 0 | |
|  |  | Asynchronous/ pre-recorded | | Video/DVD | 3 (3%) | |
|  |  |  |  | App/Web-based | 3 (3%) | |
|  |  |  |  | Exercise-based videogame | 1 (1%) | |
|  |  | Mixed | | | 11 (10%) | |
|  | Location^&^ | Community facility | | | 45 (42%) | |
|  |  | Own home | | | 24 (23%) | |
|  |  | Health facility | | | 4 (4%) | |
|  |  | Residential aged care facility | | | 11 (10%) | |
|  |  | Retirement village | | | 4 (4%) | |
|  | Equipment | Yes | | | 47 (44%) | |
|  |  | No | | | 59 (56%) | |
| Outcome^#^ | Physical activity | Steps | | | 6 (5%) | |
|  |  | Light physical activity | | | 0 | |
|  |  | Moderate-vigorous physical activity | | | 21 (16%) | |
|  |  | Up-time | | | 0 | |
|  |  | Overall physical activity | | | 28 (26%) | |
|  |  | Domains of physical activity | | | 14 (10%) | |
|  | Falls | Rate of falls | | | 34 (26%) | |
|  |  | Falls related injuries | | | 12 (9%) | |
|  | Intrinsic capacity: Physical domain | Bone mineral density | | | 8 (6%) | |
|  |  | Body composition | | | 14 (11%) | |
|  |  | Cardiometabolic indicators | | | 14 (11%) | |
|  |  | Strength | | | 35 (27%) | |
|  |  | Fitness | | | 13 (10%) | |
|  |  | Pain | | | 5 (4%) | |
|  |  | Other | | | 4 (4%) | |
|  | Functional ability: Physical domain | Mobility and balance | | | 67 (51%) | |
|  |  | Self-care | | | 13 (10%) | |
|  |  | Overall function | | | 24 (18%) | |
|  |  | Other | | | 5 (4%) | |
|  | Functional ability: Social domain | Self-report | | | 11 (8%) | |
|  |  | Observation | | | 0 | |
|  | Cognitive and emotional functioning | Cognitive | | | 25 (19%) | |
|  |  | Emotional | | | 19 (14%) | |
|  |  | Both | | | 7 (5%) | |
|  | Well-being and quality of life | Well-being | | | 0 | |
|  |  | Quality of life | | | 22 (17%) | |

* Percentages reported refer to the number of interventions (n=106) as some studies had more than two groups

^@^ The total percentage is more than 100% as individual interventions may involve more than one component

# Percentages refers to number of comparisons (n=131) not the number of studies (n=87)

^ Median and Standard Deviation (SD) only calculated for studies that reported the data

^&^  A simplified version of the location classification is presented int his table. Each study was classified according to its main location. Ccommunity centre includes public housing complex (n=1), workplace (n=0), faith-based facility (n=3), parks/sports fields (n=5) and research centre (n=15).

# APPENDIX 8. Description of studies included in Objective 2 according to intervention location

## Table A.8.1. Description of studies of physical activity interventions for older adults conducted in a community facility (k=38 studies)

| **Author (Year) or name of study***  **Country (Income level)** | **Study population & PEDro Score** | **Intervention details** | **Effect of reported outcomes** (# of outcomes, % of positive outcomes, % positive & significant outcomes)** |
| --- | --- | --- | --- |
| Aibar-Almazan et al (2019)  Spain (High-income) | **N (randomised):** 110  **Inclusion criteria:**  *- Age:* ≥60 years  *Gender:* Female  *- Physical impairment/limitation:* No  *- Emotional/cognitive status:* No  **PEDro Score:** 7 | **Type of program:** PA delivered  **Type of PA**: Recreation  **PA classification:** Pilates/Yoga  **Additional strategies:** No  **Length of intervention:** 12 weeks  **Supervised dose (in person):**  *-Frequency (times/week):* 2  *-Duration (min):* 60  **Delivered by:** Well-trained instructor  **Performed with:** Group  **Equipment needed:** Yes (elastic bands, magic circles, and fit balls) | **Functional ability:** Cognitive and emotional domain  *- # of outcomes:* 2  *- % of positive:* 100  *- % of positive & significant:* 100 |
| Barnett et al (2003)  Australia (High-income) | **N (randomised):** 163  **Inclusion criteria:**  *- Age:* ≥65 years  *Gender:* Any  *- Physical impairment/limitation:* Yes (fall risk)  *- Emotional/cognitive status:* No  **PEDro Score:** 8 | **Type of program:** PA delivered  **Type of PA**: Structured exercise  **PA classification:** Multicomponent (balance, strength)  **Additional strategies:** No  **Length of intervention:** 37 weeks  **Supervised dose (in person):**  *-Frequency (times/week):* 1  *-Duration (min):* 60  **Prescribed by:** Physiotherapist  **Delivered by:** Accredited exercise instructor trained  **Performed with:** Group  **Equipment needed:** Yes (resistance bands) | **Falls and fall related injuries**  *- # of outcomes:* 6  *- % of positive:* 100  *- % of positive & significant:* 33  **Functional ability:** Physical domain  *- # of outcomes:* 3  *- % of positive:* 100  *- % of positive & significant:* 100 |
| Best et al (2015)  Canada (High-income) | **N (randomised):** 204  **Inclusion criteria:**  *- Age:* ≥65 years  *Gender:* Female  *- Physical impairment/limitation:* No  *- Emotional/cognitive status:* No  **PEDro Score:** 7 | **Arm A**  **Type of program:** PA delivered  **Type of PA**: Structured exercise  **PA classification:** Strength  **Additional strategies:** Behavioural change strategies (mentoring, social activity, semi-monthly newsletters)  **Length of intervention:** 52 weeks  **Supervised dose (in person):**  *-Frequency (times/week):* 2  *-Duration (min):* 60  **Prescribed by:** Fitness instructor  **Delivered by:** Certified fitness instructor  **Performed with:** Self  **Equipment needed:** Yes (Keiser pressurized air system and free weights)  **Arm B**  **Type of program:** PA delivered  **Type of PA**: Structured exercise  **PA classification:** Strength  **Additional strategies:** Behavioural change strategies (mentoring, social activity, semi-monthly newsletters)  **Length of intervention:** 52 weeks  **Supervised dose (in person):**  *-Frequency (times/week):* 1  *-Duration (min):* 60  **Prescribed by:** Fitness instructor  **Delivered by:** Certified fitness instructor  **Performed with:** Self  **Equipment needed:** Yes (Keiser pressurized air system and free weights) | **Arm A**  **Intrinsic capacity:** Physical domain  *- # of outcomes:* 1  *- % of positive:* 100  *- % of positive & significant:* 100  **Functional ability:** Cognitive and emotional domain  *- # of outcomes:* 2  *- % of positive:* 100  *- % of positive & significant:* 100  **Arm B**  **Intrinsic capacity:** Physical domain  *- # of outcomes:* 1  *- % of positive:* 100  *- % of positive & significant:* 0  **Functional ability:** Cognitive and emotional domain  *- # of outcomes:* 2  *- % of positive:* 100  *- % of positive & significant:* 50 |
| Bogaerts et al (2009)  Belgium (High- income) | **N (randomised):** 286  **Inclusion criteria:**  *- Age:* ≥60 years  *Gender:* Any  *- Physical impairment/limitation:* Yes (mobility)  *- Emotional/cognitive status:* No  **PEDro Score:** 6 | **Arm A**  **Type of program:** PA delivered  **Type of PA**: Structured exercise  **PA classification:** Strength  **Additional strategies:** No  **Length of intervention:** 52 weeks  **Supervised dose (in person):**  *-Frequency (times/week):* 3  *-Duration (min):* 75  **Prescribed by:** Study team  **Delivered by:** Qualified health and fitness instructors  **Performed with:** Group  **Equipment needed:** No  **Arm B**  **Type of program:** PA delivered  **Type of PA**: Structured exercise  **PA classification:** Multicomponent (balance and strength)  **Additional strategies:** No  **Length of intervention:** 52 weeks  **Supervised dose (in person):**  *-Frequency (times/week):* 3  *-Duration (min):* 40  **Prescribed by:** Study team  **Delivered by:** Qualified health and fitness instructors  **Performed with:** Group  **Equipment needed:** No | **Arm A**  **Intrinsic capacity:** Physical domain  *- # of outcomes:* 4  *- % of positive:* 100  *- % of positive & significant:* 100  **Arm B**  **Intrinsic capacity:** Physical domain  *- # of outcomes:* 4  *- % of positive:* 100  *- % of positive & significant:* 100 |
| Exercise for Independent living study)  Australia (High-income)  Day (2012)  Day (2015) | **N (randomised):** 503  **Inclusion criteria:**  *- Age:* ≥70 years  *Gender:* Any  *- Physical impairment/limitation:* Yes (mobility)  *- Emotional/cognitive status:* No  **PEDro Score:** 6 | **Type of program:** PA delivered  **Type of PA**: Recreation  **PA classification:** Modified Sun style Tai Chi  **Additional strategies:** No  **Length of intervention:** 24 weeks  **Supervised dose (in person):**  *-Frequency (times/week):* 2  *-Duration (min):* 60  **Prescribed by:** Physiotherapist  **Delivered by:** Qualified and experienced exercise leaders  **Performed with:** Group  **Equipment needed:** No | **Day et al (2012)**  **Intrinsic capacity:** Physical domain  *- # of outcomes:* 3  *- % of positive:* 33  *- % of positive & significant:* 0  **Functional ability:** Physical domain  *- # of outcomes:* 4  *- % of positive:* 75  *- % of positive & significant:* 0  **Functional ability**: Social domain  *- # of outcomes:* 6  *- % of positive:* 50  *- % of positive & significant:* 0  **Day et al (2015)**  **Falls and fall related injuries**  *- # of outcomes:* 4  *- % of positive:* 100  *- % of positive & significant:* 50 |
| Doi et al (2017)  Japan (High-income) | **N (randomised):** 134  **Inclusion criteria:**  *- Age:* ≥65 years  *- Gender:* Any  *- Physical impairment/limitation:* No  *- Emotional/cognitive status:* Yes (mild cognitive impairment)  **PEDro Score:** 8 | **Type of program:** PA delivered  **Type of PA**: Recreation  **PA classification:** Dance  **Additional strategies:** No  **Length of intervention:** 40 weeks  **Supervised dose (in person):**  *-Frequency (times/week):* 1  *-Duration (min):* 60  **Delivered by:** Professional dance instructor  **Performed with:** Group  **Equipment needed:** No | **Functional ability:** Cognitive and emotional domain  *- # of outcomes:* 5  *- % of positive:* 100  *- % of positive & significant:* 40 |
| El-Khoury (2015)  France (high-income) | **N (randomised):** 706  **Inclusion criteria:**  *- Age:* ≥75 years  *Gender:* Female  *- Physical impairment/limitation:* Yes (Fall risk)  *- Emotional/cognitive status:* No  **PEDro Score:** 6 | **Type of program:** PA delivered  **Type of PA**: Structured exercise  **PA classification:** Multicomponent (balance, and strength)  **Additional strategies:** No  **Length of intervention:** 104 weeks  **Supervised dose (in person):**  *-Frequency (times/week):* 0.9  *-Duration (min):* 60  **Unsupervised dose**:  *-Frequency (times/week):* 1  *-Duration (min):* 30  **Prescribed by:** Trained instructor  **Delivered by:** Trained instructor and self  **Performed with:** Group  **Equipment needed:** Yes (balls) | **Physical activity**  *- # of outcomes:* 6  *- % of positive:* 100  *- % of positive & significant:* 0  **Falls and fall related injuries**  *- # of outcomes:* 4  *- % of positive:* 100  *- % of positive & significant:* 75  **Functional ability:** Physical domain  *- # of outcomes:* 5  *- % of positive:* 100  *- % of positive & significant:* 100  **Functional ability:** Cognitive and emotional domain  *- # of outcomes:* 2  *- % of positive:* 100  *- % of positive & significant:* 0 |
| Fan et al (2020)  China (Upper-middle income) | **N (randomised):** 139  **Inclusion criteria:**  *- Age:* ≥60 years  *Gender:* Any  *- Physical impairment/limitation:* No  *- Emotional/cognitive status:* No  **PEDro Score:** 6 | **Type of program:** PA delivered  **Type of PA**: Recreation  **PA classification:** Tai Chi (Baduanjin exercise programs)  **Additional strategies:** Behavioural change strategies (recorded attendance)  **Length of intervention:** 24 weeks  **Supervised dose (in person):**  *-Frequency (times/week):* 5  *-Duration (min):* 45  **Delivered by:** Trained tutor  **Performed with:** Group  **Equipment needed:** No | **Intrinsic capacity:** Physical domain  *- # of outcomes:* 1  *- % of positive:* 100  *- % of positive & significant:* 0  **Functional ability:** Physical domain  *- # of outcomes:* 2  *- % of positive:* 100  *- % of positive & significant:* 0  **Functional ability:** Cognitive and emotional domain  *- # of outcomes:* 3  *- % of positive:* 100  *- % of positive & significant:* 0  **Functional ability:** Social domain  *- # of outcomes:* 1  *- % of positive:* 100  *- % of positive & significant:* 0  **Wellbeing & QoL**  *- # of outcomes:* 1  *- % of positive:* 100  *- % of positive & significant:* 0 |
| Fitzgerald et al (2018)  United States (High-income) | **N (randomised):** 893  **Inclusion criteria:**  *- Age:* ≥70 years  *Gender:* Any  *- Physical impairment/limitation:* Yes (Fall risk)  *- Emotional/cognitive status:* No  **PEDro Score: 7** | **Arm A**  **Type of program:** PA delivered  **Type of PA**: Recreation  **PA classification:** Tai Chi  **Additional strategies:** No  **Length of intervention:** 24 weeks  **Supervised dose (in person):**  *-Frequency (times/week):* 2  *-Duration (min):* 60  **Prescribed by:** Class instructors  **Delivered by:** Trained instructor and self  **Performed with:** Group  **Equipment needed:** No  **Arm B**  **Type of program:** PA delivered  **Type of PA**: Structured exercise  **PA classification:** Multicomponent (balance, strength, and endurance)  **Additional strategies:** No  **Length of intervention:** 24 weeks  **Supervised dose (in person):**  *-Frequency (times/week):* 2  *-Duration (min):* 60  **Prescribed by:** Class instructors  **Delivered by:** Trained instructor and self  **Performed with:** Group  **Equipment needed:** Yes (gym-based equipment (hand and ankle weights, resistance tubing, balance foams) | **Arm A**  **Falls and fall related injuries**  *- # of outcomes:* 6  *- % of positive:* 100  *- % of positive & significant:* 83  **Functional ability:** Physical domain  *- # of outcomes:* 6  *- % of positive:* 100  *- % of positive & significant:* 100  **Functional ability:** Cognitive and emotional domain  *- # of outcomes:* 1  *- % of positive:* 100  *- % of positive & significant:* 100  **Arm B**  **Falls and fall related injuries**  *- # of outcomes:* 6  *- % of positive:* 100  *- % of positive & significant:* 83  **Functional ability:** Physical domain  *- # of outcomes:* 6  *- % of positive:* 100  *- % of positive & significant:* 100  **Functional ability:** Cognitive and emotional domain  *- # of outcomes:* 1  *- % of positive:* 100  *- % of positive & significant:* 100 |
| Freiberger et al (2012)  Germany (High-income) | **N (randomised):** 287  **Inclusion criteria:**  *- Age:* ≥70 years  *Gender:* Any  *- Physical impairment/limitation:* Yes (Fall risk)  *- Emotional/cognitive status:* No  **PEDro Score:** 8 | **Arm A**  **Type of program:** PA delivered  **Type of PA**: Structured exercise  **PA classification:** Multicomponent (balance, and strength)  **Additional strategies:** Yes, other (monthly supervision meeting)  **Length of intervention:** 16 weeks  **Supervised dose (in person):**  *-Frequency (times/week):* 2  *-Duration (min):* 60  **Delivered by:** Trained falls prevention instructor  **Performed with:** Group  **Equipment needed:** Yes (dumbbells, balls, obstacles)  **Arm B**  **Type of program:** PA delivered  **Type of PA**: Structured exercise  **PA classification:** Multicomponent (balance, strength, and endurance)  **Additional strategies:** Yes, other (monthly supervision meeting)  **Length of intervention:** 16 weeks  **Supervised dose (in person):**  *-Frequency (times/week):* 2  *-Duration (min):* 60  **Delivered by:** Trained falls prevention instructor  **Performed with:** Group  **Equipment needed:** Yes (dumbbells, balls, obstacles) | **Arm A**  **Functional ability:** Physical domain  *- # of outcomes:* 6  *- % of positive:* 83  *- % of positive & significant:* 33  **Arm B**  **Functional ability:** Physical domain  *- # of outcomes:* 6  *- % of positive:* 100  *- % of positive & significant:* 67 |
| Greendale et al (2009)  United States (High-income) | **N (randomised):** 118  **Inclusion criteria:**  *- Age:* ≥60 years  *Gender:* Any  *- Physical impairment/limitation:* No  *- Emotional/cognitive status:* No  **PEDro Score:** 8 | **Type of program:** PA delivered  **Type of PA**: Recreation  **PA classification:** Yoga  **Additional strategies:** No  **Length of intervention:** 24 weeks  **Supervised dose (in person):**  *-Frequency (times/week):* 3  *-Duration (min):* 60  **Delivered by:** Yoga instructor  **Performed with:** Group  **Equipment needed:** No | **Functional ability:** Physical domain  *- # of outcomes:* 3  *- % of positive:* 100  *- % of positive & significant:* 0 |
| King et al (2000)  United States (High-income) | **N (randomised):** 103  **Inclusion criteria:**  *- Age:* ≥65 years  *Gender:* Any  *- Physical impairment/limitation:* No  *- Emotional/cognitive status:* No  **PEDro Score:** 6 | **Type of program:** PA delivered  **Type of PA**: Structured exercise  **PA classification:** Multicomponent (strength, walking/wheeling, and endurance)  **Additional strategies:** Behavioural change strategy (mentoring)  **Length of intervention:** 52 weeks  **Supervised dose (in person):**  *-Frequency (times/week):* 2.33  *-Duration (min):* 53.5  **Unsupervised dose:**  *-Frequency (times/week):* 2  *-Duration (min):* 40  **Delivered by:** Instructors  **Performed with: S**elf and others  **Equipment needed:** Yes (resistive bands, step tech, homemade hand weights) | **Physical activity**  *- # of outcomes:* 1  *- % of positive:* 100  *- % of positive & significant:* 100  **Intrinsic capacity:** Physical domain  *- # of outcomes:* 3  *- % of positive:* 100  *- % of positive & significant:* 67  **Functional ability:** Physical domain  *- # of outcomes:* 1  *- % of positive:* 100  *- % of positive & significant:* 100 |
| Klusmann et al (2010)  Germany (High-income) | **N (randomised):** 167  **Inclusion criteria:**  *- Age:* ≥70 years  *Gender:* Female  *- Physical impairment/limitation:* No  *- Emotional/cognitive status:* No  **PEDro Score:** 8 | **Type of program:** PA delivered  **Type of PA**: Structured exercise  **PA classification:** Multicomponent (balance, strength, and endurance)  **Additional strategies:** No  **Length of intervention:** 24 weeks  **Supervised dose (in person):**  *-Frequency (times/week):* 3.1  *-Duration (min):* 90  **Prescribed by:** Sports physician  **Delivered by:** Trained course instructors  **Performed with:** Group  **Equipment needed:** Yes (bicycle ergometers, treadmills, pulse monitors) | **Intrinsic capacity:** Physical domain  *- # of outcomes:* 1  *- % of positive:* 100  *- % of positive & significant:* 100  **Functional ability:** Cognitive and emotional domain  *- # of outcomes:* 4  *- % of positive:* 100  *- % of positive & significant:* 100 |
| Komulainen (2010)  Finland (High-income) | **N (randomised):** 468  **Inclusion criteria:**  *- Age:* ≥50 years  *Gender:* Any  *- Physical impairment/limitation:* No  *- Emotional/cognitive status:* No  **PEDro Score:** currently being rated | **Type of program:** PA delivered  **Type of PA**: Structured exercise  **PA classification:** Strength, resistance, power  **Additional strategies:** Behaviour change strategy (face-to-face counselling)  **Length of intervention:** 104 weeks  **Supervised dose (in person):**  *-Frequency (times/week):* 3  *-Duration (min):* not clear  **Delivered by:** Exercise physiologist  **Performed with:** Self  **Equipment needed:** No | **Physical activity**  *- # of outcomes:* 3  *- % of positive:* 33  *- % of positive & significant:* 33  **Functional ability:** Cognitive and emotional domain  *- # of outcomes:* 7  *- % of positive:* 71  *- % of positive & significant:* 0 |
| Lam et al  (2014)  Hong Kong (High-income country) | **N (randomised):** 389  **Inclusion criteria:**  *- Age:* ≥65 years  *Gender:* Any  *- Physical impairment/limitation:* No  *- Emotional/cognitive status:* Yes (Mild cognitive impairment)  **PEDro Score:** 8 | **Type of program:** PA delivered  **Type of PA**: Recreation  **PA classification:** Tai Chi  **Additional strategies:** No  **Length of intervention:**  **Supervised dose (in person):**  *-Frequency (times/week):* 3  *-Duration (min):* 30  *-Length of intervention:* 6 weeks  **Unsupervised dose**:  *-Frequency (times/week):* 3  *-Duration (min):* 30  *-Length of intervention:* 52  **Delivered by:** Certified Tai Chi master, self  **Performed with:** self and others  **Equipment needed:** No | **Functional ability:** Physical domain  *- # of outcomes:* 2  *- % of positive:* 50  *- % of positive & significant:* 0  **Functional ability:** Cognitive and emotional domain  *- # of outcomes:* 1  *- % of positive:* 100  *- % of positive & significant:* 0 |
| Lazarou et al (2017)  Greece (High-income) | **N (randomised):** 129  **Inclusion criteria:**  *- Age:* ≥50 years  *- Gender:* Any  *- Physical impairment/limitation:* No  *- Emotional/cognitive status:* Yes (mild-cognitive impairment)  **PEDro Score:** 6 | **Type of program:** PA delivered  **Type of PA**: Recreation  **PA classification:** Dance (International ball room dancing)  **Additional strategies:** No  **Length of intervention:** 40 weeks  **Supervised dose (in person):**  *-Frequency (times/week):* 2  *-Duration (min):* 60  **Delivered by:** Experience dance instructor  **Performed with:** Group  **Equipment needed:** No | **Functional ability:** Cognitive and emotional domain  *- # of outcomes:* 12  *- % of positive:* 100  *- % of positive & significant:* 92 |
| Lifestyle integrated Functional Exercise (LiFE) Study US  Pilot    United States (High-income)  Wiliamson et al (2009)  Pahor et al (2006) | **N (randomised):**102  **PEDro Score:** 6  **N (randomised):** 424  **PEDro Score:** 7  **Inclusion criteria:**  *- Age*: ≥ 70-85 years  *-Physical impairment/limitation:* Yes (low PA levels, low mobility) | **Type of program:** PA delivered  **Type of PA**: Structured exercise program  **PA classification:** Multicomponent (balance, strength, walking/wheeling)  **Additional strategies:** Behaviour change strategy (mentoring, social activity)  **Length of intervention:** 48 weeks  **Supervised dose (in person):**  *-Frequency (times/week*): 2.4 (average – decreasing over first 2 months)  *-Duration (min):* 44  **Unsupervised dose**:  *-Frequency (times/week):* 3.5(average – increasing in last 10 months)  *-Duration (min):* 30  **Prescribed by:** Physical activity leader  **Delivered by:** Not specified  **Performed with:** Self  **Equipment needed:** No | **Physical activity**  *- # of outcomes:*2  *- % of positive:*100  *- % of positive & significant:*100  **Functional ability:** Physical domain  *- # of outcomes:*2  *- % of positive:*100  *- % of positive & significant:*100  **Functional ability:** Cognitive and emotional domain  *- # of outcomes:*4  *- % of positive:*75  *- % of positive & significant:*25 |
| Lifestyle integrated Functional Exercise (LiFE) Study US  United States (High-income)  Pahor (2014)  Gill (2016)  Sink (2015)  Trombetti (2018) | **N (randomised):** 1635  **Inclusion criteria:**  *- Age:* ≥70 years  *- Gender:* Any  *- Physical impairment/limitation:* Yes (mobility)  *- Emotional/cognitive status:* No  **PEDro Score:** 6 | **Type of program:** PA delivered  **Type of PA**: Structured exercise  **PA classification:** Multicomponent (balance, strength, walking-wheeling)  **Additional strategies:** Behaviour change strategy (social cognitive theory combined with strategies derived from Transtheoretical Model)  **Length of intervention:** 135 weeks  **Supervised dose (in person):**  *-Frequency (times/week):* 2  *-Duration (min):* 60  **Unsupervised dose**:  *-Frequency (times/week):* 3.5  *-Duration (min):* Individualised  **Delivered by:** Health educator  **Performed with:** Self and group  **Equipment needed:** Yes (ankle weights) | **Pahor et al**  **Physical activity**  *- # of outcomes:* 3  *- % of positive:* 100  *- % of positive & significant:* 100  **Functional ability:** Physical domain  *- # of outcomes:* 2  *- % of positive:* 100  *- % of positive & significant:* 100  **Gill et al**  **Falls and fall related injuries**  *- # of outcomes:*3  *- % of positive:* 100  *- % of positive & significant:* 0  **Sink et al**  **Functional ability:** Cognitive and emotional domain  *- # of outcomes:* 13  *- % of positive:* 46  *- % of positive & significant:* 0  **Trombetti et al**  **Functional ability:** Physical domain  *- # of outcomes:* 2  *- % of positive:* 100  *- % of positive & significant:* 50  **Functional ability:** Cognitive and emotional domain  *- # of outcomes:* 7  *- % of positive:* 71  *- % of positive & significant:* 0 |
| Lipsitz et al (2019)  United States (High-income) | **N (randomised):** 180  **Inclusion criteria:**  *- Age:* ≥60 years  *- Gender:* Any  *- Physical impairment/limitation:* No  *- Emotional/cognitive status:* No  **PEDro Score:** 6 | **Type of program:** PA delivered  **Type of PA**: Recreation  **PA classification:** Tai Chi, balance, strength  **Additional strategies:** No  **Length of intervention:** 24 weeks  **Supervised dose (in person):**  *-Frequency (times/week):* 2  *-Duration (min):* 60  **Unsupervised dose**:  *-Frequency (times/week):* 3  *-Duration (min):* 20  **Prescribed by:** Senior instructor  **Delivered by:** Tai Chi instructors  **Performed with:** Group  **Equipment needed:** No | **Physical activity:**  *- # of outcomes:* 1  *- % of positive:* 100  *- % of positive & significant:* 0  **Falls and fall related injuries**  *- # of outcomes:* 1  *- % of positive:* 0  *- % of positive & significant:* 0  **Functional ability**: Physical domain  *- # of outcomes:* 4  *- % of positive:* 25  *- % of positive & significant:* 0  **Functional ability:** Cognitive and emotional domain  *- # of outcomes:* 3  *- % of positive:* 33  *- % of positive & significant:* 0 |
| Logghe et al (2009)  Netherlands (High-income) | **N (randomised):** 269  **Inclusion criteria:**  *- Age: >*70 years  *- Gender:* Any  *- Physical impairment/limitation:* No  *- Emotional/cognitive status:* No  **PEDro Score:** 8 | **Type of program:** PA delivered  **Type of PA**: Recreation  **PA classification:** Tai Chi  **Additional strategies:** No  **Length of intervention:** 13 weeks  **Supervised dose (in person):**  *-Frequency (times/week):* 2  *-Duration (min):* 60  **Unsupervised dose**:  *-Frequency (times/week):* 2  *-Duration (min):* 15  **Prescribed by:** Tai Chi instructors  **Delivered by:** Tai Chi instructors, self at home  **Performed with:** Self and others  **Equipment needed:** No | **Physical activity**  *- # of outcomes:* 1  *- % of positive:* 100%  *- % of positive & significant:* 0  **Falls and fall related injuries**  *- # of outcomes:*1  *- % of positive:* 0  *- % of positive & significant:* 0  **Functional ability:** Physical domain  *- # of outcomes:* 2  *- % of positive:* 50  *- % of positive & significant:* 0  **Functional ability:** Cognitive and emotional domain  *- # of outcomes:* 2  *- % of positive:* 100  *- % of positive & significant:* 0 |
| Maki et al (2012)  Japan (High-income) | **N (randomised):** 150  **Inclusion criteria:**  *- Age:* ≥65 years  *- Gender:* Any  *- Physical impairment/limitation:* No  *- Emotional/cognitive status:* No  **PEDro Score:** 6 | **Type of program:** PA delivered  **Type of PA**: Structured exercise  **PA classification:** Walking-wheeling  **Additional strategies:** Behaviour change strategies (activity monitor, social activity, and incentives)  **Length of intervention:** 12 weeks  **Supervised dose (in person):**  *-Frequency (times/week):* 1  *-Duration (min):* 30  **Unsupervised dose**:  *-Frequency (times/week):* 7  *-Duration (min):* Individualised  **Delivered by:** Physical trainer or nurse  **Performed with:** Group  **Equipment needed:** No | **Intrinsic capacity:** Physical domain  *- # of outcomes:* 1  *- % of positive:* 100  *- % of positive & significant:* 100  **Functional ability:** Physical domain  *- # of outcomes:* 2  *- % of positive:* 100  *- % of positive & significant:* 100  **Functional ability:** Cognitive and emotional domain  *- # of outcomes:* 1  *- % of positive:* 100  *- % of positive & significant:* 67  **Wellbeing & QoL**  *- # of outcomes:* 1  *- % of positive:* 100  *- % of positive & significant:* 100 |
| Multicomponent exercise study  Japan (High-income)  Suzuki (2013)  Uemura (2012) | **N (randomised):** 100  **Inclusion criteria:**  *- Age:* ≥65 years  *- Gender:* Any  *- Physical impairment/limitation:* No  *- Emotional/cognitive status:* Yes (mild cognitive impairment)  **PEDro Score:** 6 | **Type of program:** PA delivered  **Type of PA**: Structured exercise  **PA classification:** Multicomponent (balance, strength, walking/wheeling, and recreation)  **Additional strategies:** Behaviour change strategies (activity monitor, booklet, attendance at each session and transport)  **Length of intervention:** 26 weeks (Suzuki et al), and 52 weeks (Uemura et al)  **Supervised dose (in person):**  *-Frequency (times/week):* 0.5  *-Duration (min):* 45  **Unsupervised dose:** 26 weeks  *-Frequency (times/week):* Individualised  *-Duration (min):* Individualised  **Delivered by:** Physiotherapists/well trained instructor  **Performed with:** Self and others  **Equipment:** No | **Suzuki et al**  **Functional ability:** Cognitive and emotional domain  *- # of outcomes:* 4  *- % of positive:* 100  *- % of positive & significant:* 100  **Uemura et al**  **Intrinsic capacity:** Physical domain  *- # of outcomes:* 6  *- % of positive:* 83  *- % of positive & significant:* 17  **Functional ability:** Physical domain  *- # of outcomes:* 1  *- % of positive:* 100  *- % of positive & significant:* 100 |
| Muscari et al (2010)  Italy (High-income) | **N (randomised):** 120  **Inclusion criteria:**  *- Age:* ≥65 years  *- Gender:* Any  *- Physical impairment/limitation:* No  *- Emotional/cognitive status:* No  **PEDro Score:** 7 | **Type of program:** PA delivered  **Type of PA**: Structured exercise  **PA classification:** Endurance (endurance exercise training)  **Additional strategies:** No  **Length of intervention:** 52 weeks  **Supervised dose (in person):**  *-Frequency (times/week):* 3  *-Duration (min):* 60  **Delivered by:** Researcher of exercise and sports  **Performed with:** Group  **Equipment needed:** Yes (cycle ergometer, treadmill) | **Intrinsic capacity:** Physical domain  *- # of outcomes:* 4  *- % of positive:* 75  *- % of positive & significant:* 75  **Functional ability:** Cognitive and emotional domain  *- # of outcomes:* 1  *- % of positive:* 100  *- % of positive & significant:* 67 |
| Music-based multitask training study  Switzerland (High-income)  Hars (2014)  Trombetti (2011) | **N (randomised):** 134  **Inclusion criteria:**  *- Age:* ≥65 years  *- Gender:* Any  *- Physical impairment/limitation:* Yes (fall risk)  *- Emotional/cognitive status:* No  **PEDro Score:** 6 | **Type of program:** PA delivered  **Type of PA**: Recreation  **PA classification:** Dance (multitask exercises)  **Additional strategies:** No  **Length of intervention:** 25 weeks  **Supervised dose (in person):**  *-Frequency (times/week):* 1  *-Duration (min):* 60  **Delivered by:** Experienced instructor  **Performed with:** Group  **Equipment needed:** No | **Hars et al**  **Functional ability:** Cognitive and emotional domain  *- # of outcomes:* 5  *- % of positive:* 80  *- % of positive & significant:* 20  **Trombetti et al**  **Falls and fall related injuries**  *- # of outcomes:*1  *- % of positive:* 100  *- % of positive & significant:* 100  **Functional ability:** Physical domain  *- # of outcomes:* 29  *- % of positive:* 86  *- % of positive & significant:* 31 |
| Pandya et al (2020)  Singapore (High-income), South Africa (Upper-middle income), Kenya (low-middle income), India (Low-middle income) | **N (randomised):** 981  **Inclusion criteria:**  *- Age:* ≥60 years  *- Gender:* Any  *- Physical impairment/limitation:* No  *- Emotional/cognitive status:* No  **PEDro Score:** 7 | **Type of program:** PA delivered  **Type of PA**: Recreation  **PA classification:** Yoga  **Additional strategies:** Behaviour change strategy (record practice lesion in a notebook maintainer by trainer)  **Length of intervention:** 260 weeks  **Supervised dose (in person):**  *-Frequency (times/week):* 1  *-Duration (min):* 40  **Prescribed by:** Yoga experts  **Delivered by:** Four yoga trainers based in four cities  **Performed with:** Group  **Equipment needed:** Yes (yoga mattress, loose comfortable clothing) | **Functional ability:** Cognitive and emotional domain  *- # of outcomes:* 2  *- % of positive:* 100  *- % of positive & significant:* 100 |
| Petrella et al (2003)  Canada (High-income) | **N (randomised):** 284  **Inclusion criteria:**  *- Age:* >65 years  *- Gender:* Any  *- Physical impairment/limitation:* No  *- Emotional/cognitive status:* No  **PEDro Score:** 6 | **Type of program:** PA promoted  **Type of PA**: Coaching  **PA classification:** Overall activity  **Additional strategies:** No  **Length of intervention:** 52 weeks  **Supervised dose (in person):**  *-Frequency (times/week):* 0.77  *-Duration (min):* 11.7  **Unsupervised dose**:  *-Frequency (times/week):* 3  *-Duration (min):* 45  **Prescribed by:** Physician  **Delivered by:** N/A (home exercise program)  **Performed with:** Self  **Equipment needed:** No | **Intrinsic capacity:** Physical domain  *- # of outcomes:* 3  *- % of positive:* 100  *- % of positive & significant:* 100  **Functional ability:** Physical domain  *- # of outcomes:* 1  *- % of positive:* 100  *- % of positive & significant:* 100 |
| ProAct65+ study  United Kingdom (High-income)  Duckham et al (2015)  (Results from only one geographic location for bone mineral density)  Iliffe et al (2014)  (Results from all sites) | **N (randomised):** 231  **Inclusion criteria:**  *- Age:* ≥65 years  *- Gender:* Any  *- Physical impairment/limitation:* No  *- Emotional/cognitive status:* No  **PEDro Score:** 6  **N (randomised)**=845 | **Arm B *****  **Type of program:** PA Delivered  **Type of PA:** Structured exercise  **PA classification:** Multicomponent (balance, strength, walking/wheeling)  **Additional strategies:** Behaviour change strategy (booklets, telephone follow-up)  **Length of intervention**: 24 weeks  **Supervised dose (in person):**  *-Frequency (times/week):* 1  *-Duration (min):* 60  **Unsupervised dose:**  *-Frequency (times/week):* 4  *-Duration (min):* 30  **Delivered by:** Postural stability instructor  Performed with: Self and others  Equipment needed: Yes (ankle cuffs weights, theraband, mats) | **Duckham et al**  **Intrinsic capacity:** Physical domain  *- # of outcomes:* 12  *- % of positive:* 42  *- % of positive & significant:* 8  **Iliffe et al**  **Physical activity**  *- # of outcomes:* 5  *- % of positive:* 100  *- % of positive & significant:* 60  **Functional ability:** Physical domain  *- # of outcomes:* 5  *- % of positive:* 20  *- % of positive & significant:* 20  **Functional ability:** Social domain  *- # of outcomes:* 2  *- % of positive:* 0  *- % of positive & significant:* 0  **Functional ability:** Cognitive and emotional domain  *- # of outcomes:* 1  *- % of positive:* 0  *- % of positive & significant:* 0  **Wellbeing & QoL**  *- # of outcomes:* 2  *- % of positive:* 0  *- % of positive & significant:* 0 |
| Schluter et al (2012)  New Zealand (High-income) | **N (randomised):** 915  **Inclusion criteria:**  *- Age:* ≥65 years  *- Gender:* Any  *- Physical impairment/limitation:* No  *- Emotional/cognitive status:* No  **PEDro Score:** 6 | **Arm A**  **Type of program:** PA delivered  **Type of PA**: Recreation  **PA classification:** Modified Sun style Tai Chi  **Additional strategies:** No  **Length of intervention:** 20 weeks  **Supervised dose (in person):**  *-Frequency (times/week):* 1  *-Duration (min):* 60  **Delivered by:** Tai Chi teacher  **Performed with:** Group  **Equipment needed:** No  **Arm B**  **Type of program:** PA delivered  **Type of PA**: Recreation  **PA classification:** Modified Sun style Tai Chi  **Additional strategies:** No  **Length of intervention:** 20 weeks  **Supervised dose (in person)**:  *-Frequency (times/week):* 2  *-Duration (min):* 60  **Delivered by:** Tai Chi teacher  **Performed with:** Group  **Equipment needed:** No | **Arm A**  **Falls and fall related injuries**  *- # of outcomes:*1  *- % of positive:* 0  *- % of positive & significant:* 0  **Arm B**  **Falls and fall related injuries**  *- # of outcomes:*1  *- % of positive:* 100  *- % of positive & significant:* 0 |
| Shimada et al (2018)  Japan (High income) | **N (randomised):** 308  **Inclusion criteria:**  *- Age:* >65 years  *- Gender:* Any  *- Physical impairment/limitation:* No  *- Emotional/cognitive status:* Mild cognitive impairment  **PEDro Score:** 7 | **Type of program:** PA delivered  **Type of PA**: Structured exercise  **PA classification:** multicomponent (balance, strength, and walking)  **Additional strategies:** Behaviour change strategies (booklet, activity monitor)  **Length of intervention:** 40 weeks  **Supervised dose (in person):**  *-Frequency (times/week):* 1  *-Duration (min):* 90  **Unsupervised dose**:  *-Frequency (times/week):* 7  *-Duration (min):* 30  **Delivered by:** Physiotherapist, instructors, home program  **Performed with:** Self and others  **Equipment needed:** Steps, balance boards, HR monitor | **Physical activity**  *- # of outcomes:* 2  *- % of positive:* 100  *- % of positive & significant:* 100  **Functional ability:** Cognitive and emotional domain  *- # of outcomes:* 6  *- % of positive:* 100  *- % of positive & significant:* 67 |
| Shimada et al (2018)  Japan (High-income) | **N (randomised):** 106  **Inclusion criteria:**  *- Age:* ≥60 years  *- Gender:* Any  *- Physical impairment/limitation:* No  *- Emotional/cognitive status:* No  **PEDro Score:** 6 | **Type of program:** PA delivered  **Type of PA**: Competitive sport  **PA classification:** Golf  **Additional strategies:** No  **Length of intervention:** 24 weeks  **Supervised dose (in person)**:  *-Frequency (times/week):* 1  *-Duration (min):* 105  **Prescribed by:** Professional golfer  **Delivered by:** Professional golfer and staff member  **Performed with:** Self and others  **Equipment needed:** Yes | **Intrinsic capacity:** Physical domain  *- # of outcomes:* 1  *- % of positive:* 0  *- % of positive & significant:* 0  **Functional ability:** Physical domain  *- # of outcomes:* 1  *- % of positive:* 0  *- % of positive & significant:* 0  **Functional ability:** Cognitive and emotional domain  *- # of outcomes:* 11  *- % of positive:* 91  *- % of positive & significant:* 27 |
| Shumway-Cook (2007)  United States (High-income) | **N (randomised):** 453  **Inclusion criteria:**  *- Age:* ≥65 years  *- Gender:* Any  *- Physical impairment/limitation:* No  *- Emotional/cognitive status:* No  **PEDro Score:** 8 | **Type of program:** PA delivered  **Type of PA**: Structured exercise  **PA classification:** Multicomponent (balance, strength, and recreation)  **Additional strategies:** Behaviour change strategies, other (telephone follow-up)  **Length of intervention:** 52 weeks  **Supervised dose (in person):**  *-Frequency (times/week):* 3  *-Duration (min):* 60  **Prescribed by:** Study team  **Delivered by:** Certified fitness trainer  **Performed with:** Group  **Equipment needed:** Yes (not specified) | **Falls and fall related injuries**  *- # of outcomes:* 1  *- % of positive:* 0  *- % of positive & significant:* 0  **Functional ability:** Physical domain  *- # of outcomes:* 3  *- % of positive:* 100  *- % of positive & significant:*  100 |
| Song et al (2019)  China (Upper-middle income) | **N (randomised):** 120  **Inclusion criteria:**  *- Age:* ≥60 years  *- Gender:* Any  *- Physical impairment/limitation:* No  *- Emotional/cognitive status:* Yes (mild cognitive impairment)  **PEDro Score: 7** | **Type of program:** PA delivered  **Type of PA**: Structured exercise  **PA classification:** Endurance (aerobic exercises)  **Additional strategies:** Behaviour change strategy (motivational strategies including goal setting)  **Length of intervention:** 16 weeks  **Supervised dose (in person)**:  *-Frequency (times/week):* 3  *-Duration (min):* 60  **Prescribed by:** Nurse academician, physiotherapist, exercise physiologists  **Delivered by:** Registered nurses  **Performed with:** Group  **Equipment needed:** No | **Functional ability:** Cognitive and emotional domain  *- # of outcomes:* 2  *- % of positive:* 100  *- % of positive & significant:* 100  **Wellbeing & QoL**  *- # of outcomes:* 1  *- % of positive:* 100  *- % of positive & significant:* 100 |
| Sun et al (2016)  China (Upper-middle income) | **N (randomised):** 150  **Inclusion criteria:**  *- Age:* ≥60 years  *- Gender:* Any  *- Physical impairment/limitation:* No  *- Emotional/cognitive status:* No  **PEDro Score:** 6 | **Type of program:** PA delivered  **Type of PA**: Recreation  **PA classification:** Yang style Tai Chi  **Additional strategies:** No  **Length of intervention:** 24 weeks  **Supervised dose (in person)**:  *-Frequency (times/week):* 2  *-Duration (min):* 60  **Delivered by:** Tai Chi instructors  **Performed with:** Group  **Equipment needed:** No | **Intrinsic capacity:** Physical domain  *- # of outcomes:* 2  *- % of positive:* 100  *- % of positive & significant:* 100  **Functional ability:** Physical domain  *- # of outcomes:* 2  *- % of positive:* 100  *- % of positive & significant:* 100  **Functional ability:** Cognitive and emotional domain  *- # of outcomes:* 1  *- % of positive:* 100  *- % of positive & significant:* 100 |
| Thomas et al  (2005)  Hong Kong (High-income) | **N (randomised):** 285  **Inclusion criteria:**  *- Age:* ≥65 years  *- Gender:* Any  *- Physical impairment/limitation:* No  *- Emotional/cognitive status:* No  **PEDro Score:** 6 | **Arm A**  **Type of program:** PA delivered  **Type of PA**: Structured exercise  **PA classification: S**trength  **Additional strategies:** No  **Length of intervention:** 52 weeks  **Supervised dose (in person)**:  *-Frequency (times/week):* 3  *-Duration (min):* 60  **Delivered by:** Qualified trainer  **Performed with:** Self and others  **Equipment needed:** Yes (theraband)  **Arm B**  **Type of program:** PA delivered  **Type of PA**: Recreation  **PA classification:** Tai Chi  **Additional strategies:** No  **Length of intervention:** 52 weeks  **Supervised dose (in person)**:  *-Frequency (times/week):* 3  *-Duration (min):* 60  **Delivered by:** Tai Chi master  **Performed with:** Group  **Equipment needed:** No | **Arm A**  **Physical activity**  *- # of outcomes:* 2  *- % of positive:* 100  *- % of positive & significant:* 0  **Intrinsic capacity:** Physical domain  *- # of outcomes:* 14  *- % of positive:* 64  *- % of positive & significant:* 7  **Arm B**  **Physical activity**  *- # of outcomes:* 2  *- % of positive:* 100  *- % of positive & significant:* 0  **Intrinsic capacity:** Physical domain  *- # of outcomes:* 14  *- % of positive:* 43  *- % of positive & significant:* 0 |
| Voukelatos et al (2007)  Australia (High-income) | **N (randomised):** 702  **Inclusion criteria:**  *- Age:* ≥60 years  *Gender:* Any  *- Physical impairment/limitation:* No  *- Emotional/cognitive status:* No  **PEDro Score:** 7 | **Type of program:** PA delivered  **Type of PA**: Recreation  **PA classification:** Balance and Tai Chi  **Additional strategies:** No  **Length of intervention:** 16 weeks  **Supervised dose (in person):**  *-Frequency (times/week):* 1  *-Duration (min):* 60  **Delivered by:** Experienced Tai Chi instructors  **Performed with:** Group  **Equipment needed:** No | **Falls and fall related injuries**  *- # of outcomes:* 5  *- % of positive:* 100  *- % of positive & significant:* 20  **Functional ability:** Physical domain  *- # of outcomes:* 6  *- % of positive:* 100  *- % of positive & significant:* 83 |
| Wolf et al (1996)  United States (High-income) | **N (randomised):** 264  **Inclusion criteria:**  *- Age:* ≥70 years  *Gender:* Any  *- Physical impairment/limitation:* No  *- Emotional/cognitive status:* No  **PEDro Score:** 6 | **Type of program:** PA delivered  **Type of PA**: Recreation  **PA classification:** Tai Chi  **Additional strategies:** No  **Length of intervention:** 15 weeks  **Supervised dose (in person):**  *-Frequency (times/week):* 2  *-Duration (min):* 45  **Unsupervised dose**:  *-Frequency (times/week):* 14  *-Duration (min):* 15  **Delivered by:** Tai Chi instructor  **Performed with:** Group  **Equipment needed:** No  **Equipment needed:** No | **Intrinsic capacity:** Physical domain  *- # of outcomes:* 3  *- % of positive:* 67  *- % of positive & significant:* 33 |
| Woo et al (2007)  New Territories Region of Hong Kong, China, (High-income) | **N (randomised):** 240  **Inclusion criteria:**  *- Age:* ≥65 years  *Gender:* Any  *- Physical impairment/limitation:* No  *- Emotional/cognitive status:* No  **PEDro Score:** 6 | **Arm A (men)**  **Type of program:** PA delivered  **Type of PA**: Structured exercise  **PA classification:** Strength, resistance, power  **Additional strategies:** No  **Length of intervention:** 52 weeks  **Supervised dose (in person):**  *-Frequency (times/week):* 3  *-Duration (min):* 15  **Delivered by:** Tai Chi instructor  **Performed with:** Group  **Equipment needed:** Yes (theraband medium strength)  **Arm B - (women)**  As for arm A  **Arm C (men)**  **Type of program:** PA delivered  **Type of PA**: Recreation  **PA classification:** Tai Chi Yang style  **Additional strategies:** No  **Length of intervention:** 52 weeks  **Supervised dose (in person):**  *-Frequency (times/week):* 3  *-Duration (min):* 15  **Delivered by:** Tai Chi instructor  **Performed with:** Group  **Equipment needed:** No  **Arm D (women)**  As for arm C | **Arm A**  **Falls and fall related injuries**:  *- # of outcomes:* 1  *- % of positive:* 100  *- % of positive & significant:* 0  **Intrinsic capacity:** Physical domain  *- # of outcomes:* 2  *- % of positive:* 50  *- % of positive & significant:* 0  **Arm B**  **Falls and fall related injuries**  *- # of outcomes:* 1  *- % of positive:* 100  *- % of positive & significant:* 0  **Intrinsic capacity:** Physical domain  *- # of outcomes:* 2  *- % of positive:* 100  *- % of positive & significant:* 50  **Arm C**  **Falls and fall related injuries**  *- # of outcomes:* 1  *- % of positive:* 100  *- % of positive & significant:* 0  **Intrinsic capacity:** Physical domain  *- # of outcomes:* 2  *- % of positive:* 50  *- % of positive & significant:* 0  **Arm D**  **Falls and fall related injuries**  *- # of outcomes:* 1  *- % of positive:* 100  *- % of positive & significant:* 0  **Intrinsic capacity:** Physical domain  *- # of outcomes:* 2  *- % of positive:* 50  *- % of positive & significant:* 50 |
| Yamada et al  (2011)  Japan (High-income) | **N (randomised):** 186  **Inclusion criteria:**  *- Age:* ≥65 years  *Gender:* Any  *- Physical impairment/limitation:* No  *- Emotional/cognitive status:* No  **PEDro Score:** 7 | **Type of program:** PA delivered  **Type of PA**: Structured exercise  **PA classification:** Strength  **Additional strategies:** No  **Length of intervention:** 24 weeks  **Supervised dose (in person):**  *-Frequency (times/week):* 2  *-Duration (min):* 20  **Delivered by:** Physiotherapist  **Performed with:** Group  **Equipment needed:** No | **Functional ability:** Physical domain  *- # of outcomes:* 2  *- % of positive:* 100  *- % of positive & significant:* 100 |

*Multiple papers from the same study are only included once under a study name

** The list of outcomes presented in this table only includes outcomes investigated in these 38 studies

*** Arm A of this study presented in Table A.7.2 home setting

## Table A.8.2. Description of studies of physical activity interventions for older adults conducted fully at home (k=22 studies)

| **Author (Year) or name of study***  **Country (Income level)** | **Study population & PEDro Score** | **Intervention details** | **Effect of reported outcomes** (# of outcomes, % of positive outcomes, % positive & significant outcomes)** |
| --- | --- | --- | --- |
| Bernocchi et al (2019)  Italy (High -income) | **N (randomised):** 283  **Inclusion criteria:**  *- Age: ≥* 65 years  *- Physical impairment/limitation status:* yes (falls risk)  **PEDro Score:** 8 | **Type of program:** PA delivered  **Type of PA**: Structured exercise- home telehealth  **PA classification:** Multicomponent (balance, strength, walking/wheeling)  **Additional strategies:** Yes, behaviour change – booklet, follow up phone calls  **Length of intervention:** 24 weeks  **Supervised dose (live by phone):**  *-Frequency (times/per week):* 0.25 (initial consultation)  *-Duration (min):* 30  **Unsupervised dose**:  *-Frequency (times/week):* 2  *-Duration (min):* 30  **Prescribed by:** Physiotherapist  **Delivered by:** Physiotherapist online (telehealth)  **Performed with:** Self  **Equipment needed:** Electronic device | **Functional ability:** Physical domain  *- # of outcomes:* 3  *- % of positive:* 100  *- % of positive & significant:* 100  **Wellbeing and Quality of Life**  *- # of outcomes:* 1  *- % of positive:* 100  *- % of positive & significant:* 100 |
| Boongird et al (2017)  Thailand (Upper middle-income) | **N (randomised):** 439  **Inclusion criteria:**  *- Age: ≥* 65 years  *- Physical impairment/limitation status:* yes (low PA levels, use of a walking aid)  **PEDro Score:** 6 | **Type of program:** delivered  **Type of PA**: Structured exercise – modified OEP  **PA classification:** Multicomponent (balance, strength, walking/wheeling)  **Additional strategies:** Behaviour change strategy (follow-up phone calls)  **Length of intervention:** 52  **Supervised dose (Pre-recorded video):**  *-Frequency (times/week):*3  *-Duration (min):*30  **Prescribed by:** Nurse  **Delivered by:** Video/DVD  **Performed with:** Self  **Equipment needed:** No | **Falls and fall related injuries**  *- # of outcomes:*1  *- % of positive:*100  *- % of positive & significant:* 0  **Functional ability:** Physical domain  *- # of outcomes:* 3  *- % of positive:* 66  *- % of positive & significant*: 0  **Functional ability:** Emotional and cognitive domain  *- # of outcomes:*1  *- % of positive:* 100  *- % of positive & significant:* 100  **Wellbeing and Quality of Life**  *- # of outcomes:*1  *- % of positive:* 100  *- % of positive & significant:* 0 |
| Campbell et al (1997)  New Zealand (High-income) | **N (randomised):** 233  **Inclusion criteria:**  *- Age:* ≥80 years  *- Gender:* women  **PEDro Score:** 8 | **Type of program:** PA delivered  **Type of PA**: Structured exercise  **PA classification:** Multicomponent (balance, strength, walking/wheeling)  **Additional strategies:** No  **Length of intervention:** 26 weeks  **Unsupervised dose:**  *-Frequency (times/week*): 6  *-Duration (min):*30  **Prescribed by:** Physiotherapist  **Delivered by:** Physiotherapist, then self  **Performed with:** Self  **Equipment needed:** Yes **(**Ankle cuff weights) | **Physical activity**  *- # of outcomes:*1  *- % of positive:*100  *- % of positive & significant:*100  **Falls and fall related injuries**  *- # of outcomes:*2  *- % of positive:*100  *- % of positive & significant:*100  **Intrinsic capacity:** Physical domain  *- # of outcomes:*1  *- % of positive:*100  *- % of positive & significant:*100  **Functional ability:** Physical domain  *- # of outcomes:*1  *- % of positive:*100  *- % of positive & significant:*100 |
| Chandler et al (1998)  United States (High-income) | **N (randomised):** 100  **Inclusion criteria:**  *- Age:* ≥ 64 years  *- Physical impairment/limitation:* yes (frailty)  **PEDro Score**: 6 | **Type of program:** PA delivered  **Type of PA**: Structured exercise  **PA classification:** Strength/resistance/power  **Additional strategies:** No  **Length of intervention:** 10 weeks  **Supervised dose (in person):**  *-Frequency (times/week):* 3  *-duration*: Not-specified  **Prescribed by:** Physical therapist  **Delivered by:** Physical therapist  **Performed with**: Self  **Equipment needed:** Theraband | **Intrinsic capacity:** Physical domain  *- # of outcomes:* 4  *- % of positive:*100  *- % of positive & significant:*75 |
| Day el al (2002)  Australia (High-income) | **N (randomised):** 272  **Inclusion criteria:**  *- Age: ≥70 years*  **PEDro Score:** 6 | **Type of program:** PA delivered  **Type of PA**: Structured  **PA classification:** Multicomponent (balance/functional/neuromotor, strength/resistance/power)  **Additional strategies:** Behaviour change strategy (mentoring)  **Length of intervention:** 52 weeks  **Supervised dose (in person):**  *-Frequency (times/week):* 1  *-Duration (min):* 60  *Delivered in group setting for 15 weeks*  **Unsupervised dose:**  *-Frequency (times/week):* 7  *-Duration (min):unspecified*  **Prescribed by:** Health professional, physiotherapist  **Delivered by:** Didn’t specify, home program  **Performed with:** Mixed (group and daily home exercise)  **Equipment needed:** No | **Falls and fall related injuries**  *- # of outcomes:*1  *- % of positive:*100  *- % of positive & significant:*100 |
| Dubbert et al (2008)  United States (High-income) | **N (randomised):** 224  **Inclusion criteria:**  *- Age: 60-85years*  *- Veterans*  *- gender: male*  *Physical impairment/limitation: low PA levels, difficulty walking/slow walking*  **PEDro Score**: 6 | **Type of program:** PA promoted – brief/very brief intervention  **Type of PA**: Overall activity  **PA classification:** N/A  **Additional strategies:** Behaviour change strategies **(**phone calls and automated motivation messages)  **Length of intervention:** 40 weeks  **Supervised dose (in person):**  *-Frequency (times/week):* 0.075 (3 visits over 10 months)  *-Duration (min):* 30  **Unsupervised dose**:  *-Frequency (times/week):* 6  *-Duration (min):* 45  **Prescribed by:** Nurse  **Delivered by:** Self  Performed with: Self  **Equipment needed:** Dumb bells, ankle weights | **Physical activity**  *- # of outcomes:8*  *- % of positive:100*  *- % of positive & significant:75*  **Functional ability:** Physical domain  *- # of outcomes:4*  *- % of positive:75*  *- % of positive & significant:50* |
| ProAct65+study  United Kingdon (High-income)  Duckham et al (2015)  (Results from only one geographic location for bone mineral density)  Iliffe et al (2014)  (results from all sites) | **N (randomised):** 231  **Inclusion criteria:**  *- Age:* ≥65 years  *- Gender:* Any  *- Physical impairment/limitation:* No  *- Emotional/cognitive status:* No  **PEDro Score:** 6  **N (randomised)**=845 | **Arm A *****  **Type of program:** PA delivered  **Type of PA**: Structured - OEP  **PA classification:** Multicomponent (balance, strength, walking/wheeling)  **Additional strategies:** Yes, behaviour change - mentoring  **Length of intervention:** 24 weeks  **Unsupervised dose**:  *-Frequency (times/week):* 5  *-Duration (min):* 30  **Prescribed by:** Trained research staff  **Delivered by:** Self  **Performed with:** Self  **Equipment needed:** Ankle cuff weights | **Duckham et al (2015)**  **Intrinsic capacity:** Physical domain  *- # of outcomes:*12  *- % of positive:*50  *- % of positive & significant:*0  **Iliffe et al (2014)**  **Physical activity**  *- # of outcomes:* 5  *- % of positive:* 100  *- % of positive & significant*: 60  **Falls and fall related injuries**  *- # of outcomes:*1  *- % of positive:* 0  *- % of positive & significant:* 0  **Functional ability:** Physical domain  *- # of outcomes:* 5  *- % of positive:* 40  *- % of positive & significant:* 20  **Functional ability:** Social domain  *- # of outcomes:*2  *- % of positive:*0  *- % of positive & significant:*0  **Functional ability:** Emotional and cognitive domain  *- # of outcomes:*1  *- % of positive:*0  *- % of positive & significant:*0  **Wellbeing and Quality of Life**  *- # of outcomes:*2  *- % of positive:*50  *- % of positive & significant:*0 |
| Gschwind et al (2015)  Germany, Spain, Australia (High-income)  iSTOPPFalls system | **N (randomised):**153  **Inclusion criteria:**  *- Age: ≥65 years*  **PEDro Score:** 6 | **Type of program:** PA delievered  **Type of PA:** Exercise based video games  **PA classification:** Balance, strength  **Additional strategies:** Behaviour change strategy **(**booklet)  **Length of intervention:** 16 weeks  **Unsupervised dose**:  *-Frequency (times/week):* 3  *-Duration (min):* 60  **Prescribed by**: Health professional, not specified  **Delivered by:** Online  **Performed with:** Self  **Equipment needed:** No | **Functional ability:** Physical domain  *- # of outcomes:*1  *- % of positive:*100  *- % of positive & significant:*100 |
| Hsieh et al (2019)  Taiwan (High-income) | **N (randomised):** 159  **Inclusion criteria:**  *- Age:* ≥ 65 years  *- Physical impairment/limitation:* yes (frail/pre-frail)  **PEDro Score:** 6 | **Type of program:** PA delivered  **Type of PA**: Structured exercise, personally prescribed  **PA classification:** Multicomponent (balance, strength, walking/wheeling)  **Additional strategies:** Behaviour change strategies (exercise diary, inspirational cards, follow ups)  **Length of intervention:** 24 weeks  **Unsupervised dose**:  *-Frequency (times/week):* 5  *-Duration (min):* 32.5  **Prescribed by:** Physiotherapist  **Delivered by:** Self  **Performed with:** Self  **Equipment needed:** Resistance band, grip ball | **Intrinsic capacity:** Physical domain  *- # of outcomes:*2  *- % of positive:*100  *- % of positive & significant:*100  **Functional ability:** Physical domain  *- # of outcomes:*4  *- % of positive:*100  *- % of positive & significant:*100  **Functional ability:** Emotional and cognitive domain  *- # of outcomes:*6  *- % of positive:*67  *- % of positive & significant:*0 |
| Jette et al (1996)  United States (High-income) | **N (randomised):** 102  **Inclusion criteria:**  *- Age:* ≥ 65years  **PEDro Score:** 6 | **Type of program:** PA delivered  **Type of PA**: Structured exercise- progressive strengthening program  **PA classification:** Strength  **Additional strategies:** Behaviour change strategy **(**phone calls)  **Length of intervention:** 13.5 weeks  **Supervised dose (live by phone):**  *-Frequency (times/week):* 0.5 (6 phone calls plus 1 training session over 13.5 weeks)  *-Duration (min):* 15  **Unsupervised dose**:  *-Frequency (times/week):* 3  *-Duration (min):* 30  **Prescribed by:** Physical therapist  **Delivered by:** Trained instructor via DVD  **Performed with:** Self  **Equipment needed:** Theraband | **Intrinsic capacity**: Physical domain  *- # of outcomes:*4  *- % of positive:*100  *- % of positive & significant:*0 |
| Jette et al (1999)  Unites States (High-income) | **N (randomised):**215  **Inclusion criteria:**  *- Age:* ≥60 years  *- Physical impairment/limitation:* Yes (at least one functional limitation from short health survey)  **PEDro Score:** 7 | **Type of program:** PA delivered  **Type of PA**: Structured exercise - progressive resistance program  **PA classification:** Strength  **Additional strategies:** Behaviour change strategies (incentives; cognitive behavioural strategies, telephone contact, individualised goal setting)  **Length of intervention:** 26 weeks  **Supervised dose (in person):**  *-Frequency (times/week):* 0.076 - 2 visits in 26 weeks  *-Duration (min):* 30  **Unsupervised dose**:  *-Frequency (times/week):* 3  *-Duration (min):* 35  **Prescribed by:** Physical therapist  **Delivered by:** Unspecified, 2 home visits plus DVD of class  **Performed with:** Self  **Equipment needed:** Theraband | **Intrinsic capacity:** Physical domain  *- # of outcomes:*6  *- % of positive:*75  *- % of positive & significant:*50  **Functional ability:** Physical domain  *- # of outcomes:*6  *- % of positive:*83  *- % of positive & significant:*50  **Functional ability:** Emotional and cognitive domain  *- # of outcomes:*7  *- % of positive:*43  *- % of positive & significant:*0 |
| Finnish study on weight bearing exercise in older women  Finland (High-income)  Korpelainen et al (2006) and  Korpelainen et al (2006) | **N (randomised):** 160  **Inclusion criteria:**  *- Age:* ≥70-73years  *- gender:* women  *- Physical impairment/limitation:* Yes (low bone mineral density)  **PEDro Score:** 8  **PEDro Score:** 6 | **Type of program:** PA delivered  **Type of PA**: Structured exercise – impact, balancing and strengthening exercises  **PA classification:** Multicomponent (balance, strength, walking/wheeling)  **Additional strategies:** No  **Length of intervention:** 130 weeks  **Supervised dose (in person):**  *-Frequency (times/week):* 0.6 – once a week for 6 months each year they received supervised exercises i.e. 78 supervised sessions in 130 weeks  *-Duration (min):* 60  **Unsupervised dose**:  *-Frequency (times/week):* 7  *-Duration (min):* 20  **Prescribed by:** Physical therapist  **Delivered by:** Physical therapist, self  **Performed with:** Self (occasionally in group, unspecified)  **Equipment needed:** No | **Physical activity**  *- # of outcomes:*2  *- % of positive:*100  *- % of positive & significant:*0  **Falls and fall related injuries**  *- # of outcomes:*1  *- % of positive:*100  *- % of positive & significant:*100  **Intrinsic capacity:** Physical domain  *- # of outcomes:*10  *- % of positive:*70  *- % of positive & significant:*20  **Functional ability:** Physical domain  *- # of outcomes:*3  *- % of positive:*67  *- % of positive & significant:*67  **Functional ability:** Emotional and cognitive domain  *- # of outcomes:*2  *- % of positive:*0  *- % of positive & significant:*0 |
| Krebs et al (1998)  Unites States (High-income)  Strong For Life program | **N (randomised):**132  **Inclusion criteria:**  *- Age:* ≥60years  *- Physical impairment/limitation:* Yes (low mobility)  **PEDro Score:** 6 | **Type of program:** PA delivered  **Type of PA**: Structured exercise  **PA classification:** Strength  **Additional strategies:** Behaviour change strategies (mentoring, incentives)  **Length of intervention:** 24 weeks  **Supervised dose (in person and live by phone):**  *-Frequency (times/week):* 0.4 (2 visits and 7.5 phone calls in 24 weeks)  *-Duration (min):* 12  **Unsupervised dose**:  *-Frequency (times/week):* 3  *-Duration (min):* 35  **Prescribed by:** Physical therapist  **Delivered by:** Self – video/DVD  **Performed with:** Self  **Equipment needed:** Elastic bands | **Intrinsic capacity:** Physical domain  *- # of outcomes:*1  *- % of positive:*100  *- % of positive & significant:*100  **Functional ability:** Physical domain  *- # of outcomes:*2  *- % of positive:*100  *- % of positive & significant:*50 |
| Lautenschlager et al (2008)  Australia (High-income) | **N (randomised):**170  **Inclusion criteria:**  *- Age:* ≥50 years  *- Emotional/cognitive status:* Yes (mild congnitive impairment)  **PEDro Score:** 8 | **Type of program:** PA promoted  **Type of PA**: Overall activity - modified behavioural intervention package based on social cognitive theory  **PA classification:** N/A  **Additional strategies:** Behaviour change strategies (booklet, workshop and structured phone interview)  **Length of intervention:** 24 weeks  **Supervised dose (in person and live by phone):**  *-Frequency (times/week):* 0.125 (1 interview, 2 calls)  *-Duration (min):* 27  **Unsupervised dose**:  *-Frequency (times/week):* 3  *-Duration (min):* 50  **Prescribed by:** Trained physical activity leader  **Delivered by:** Self  **Performed with:** Self  **Equipment needed:** No | **Functional ability:** Emotional and cognitive domain  *- # of outcomes:*8  *- % of positive:*88  *- % of positive & significant:*38 |
| Lifestyle integrated Functional Exercise (LiFE) Study  Australia (High-income)  Clemson et al (2012) | **Study arm A**  **N (randomised):** 212  **Inclusion criteria:**  *- Age:* ≥70years  *-* Veterans, their spouses and war widows  *- Physical impairment/limitation:* yes (falls risk)  **PEDro Score:** 7  **Study arm B**  **N (randomised):**210 | **Arm A (Lifestyle integrated Functional Exercise (LiFE)**  **Type of program:** PA delivered  **Type of PA**: Structured exercise – Lifestyle integrated functional exercise  **PA classification:** Multicomponent (balance, strength, walking/wheeling)  **Additional strategies**: Behaviour change strategies (booklet, self-report, interviews)  **Length of intervention:** 52  **Supervised dose (in person):**  *-Frequency):* 7 visits in 6 months  *-Duration (min):* 60  **Unsupervised dose**:  *-Frequency (times/week): 7*  *-Duration (min):* Integrated into day  **Prescribed by:** Health professional, physiotherapist, occupational therapist  **Delivered by:** Health professional, physiotherapist, occupational therapist (for visits), self  **Performed with:** Self  **Equipment needed:** No  **Arm B (balance and strength training)**  **Type of program:** PA delivered  **Type of PA**: Structured – modified OEP  **PA classification:** Multicomponent (balance, strength)  **Additional strategies:** No  **Length of intervention:** 52 weeks  **Supervised dose (in person):**  *-Frequency):* 7 visits in 6 months  *-Duration (min):* 60  **Unsupervised dose**:  *-Frequency (times/week):* 3  *-Duration (min):* 60  **Prescribed by:** Physiotherapist, occupational therapist  **Delivered by:** Physiotherapist, occupational therapist (for visits), self  **Performed with:** Self  **Equipment needed:** Ankle cuff weights | **Arm A**  **Physical activity**  *- # of outcomes:*2  *- % of positive:*100  *- % of positive & significant:*100  **Falls and fall related injuries**  *- # of outcomes:*1  *- % of positive:*100  *- % of positive & significant:*100  **Intrinsic capacity:** Physical domain  *- # of outcomes:*3  *- % of positive:*100  *- % of positive & significant:*66  **Functional ability:** Physical domain  *- # of outcomes:*6  *- % of positive:*100  *- % of positive & significant:*100  **Arm B**  **Physical activity**  *- # of outcomes:*2  *- % of positive:*50  *- % of positive & significant:0*  **Falls and fall related injuries**  *- # of outcomes:*1  *- % of positive:*100  *- % of positive & significant:*0  **Intrinsic capacity**: Physical domain  *- # of outcomes:*3  *- % of positive:*100  *- % of positive & significant:*0  **Functional ability:** Physical domain  *- # of outcomes:*6  *- % of positive:*100  *- % of positive & significant:*50 |
| Liu-Ambrose et al (2019)  Canada: (High-income) | **N (randomised):**345  **Inclusion criteria:**  *- Age:* ≥70 years  *- Physical impairment/limitation:* Yes (falls risk)  **PEDro Score:** 8 | **Type of program:** PA delivered  **Type of PA**: Structured exercise- strength and balance program  **PA classification:** Multicomponent (balance, strength)  **Additional strategies:** No  **Length of intervention:** 52 weeks  **Supervised dose (in person):**  *-Frequency (times/week):* 0.076 (4 visits in 12 months)  *-Duration (min):* 60  **Unsupervised dose**:  *-Frequency (times/week):* 5  *-Duration (min):* 48  **Prescribed by:** Physical therapist  **Delivered by:** Physical therapist**,** self  **Performed with:** Self  **Equipment needed:** Cuff weights | **Falls and fall related injuries**  *- # of outcomes:*2  *- % of positive:*50  *- % of positive & significant:*50  **Functional ability:** Physical domain  *- # of outcomes:*3  *- % of positive:*33  *- % of positive & significant:*0  **Functional ability:** Emotional and cognitive domain  *- # of outcomes:*5  *- % of positive:*40  *- % of positive & significant:*0 |
| Finnish Study  Finland (High- income)  Luukinen et al (2006) and  Luukinen et al (2007) | **N (randomised):** 486  **Inclusion criteria:**  *- Age:* ≥ 85 years  *- Physical impairment/limitation:*  Yes **(**at least 1 risk factor out of 9 for disability on health survey)  *-Emotion or cognition status:* Yes (symptoms of depression)  **PEDro Score**: 6 | **Type of program:** PA delivered  **Type of PA**: Structured exercise  **PA classification:** Multicomponent (balance, strength, walking/wheeling)  **Additional strategies:** No  **Length of intervention:** 69 weeks  **Unsupervised dose**:  *-Frequency (times/week):* 7  *-Duration (min):* 20  **Prescribed by:** Physiotherapist  **Delivered by:** Physiotherapist  **Performed with:** Self  **Equipment needed:** No | **Physical activity**  *- # of outcomes:*3  *- % of positive:*100  *- % of positive & significant:*0  **Falls and fall related injuries**  *- # of outcomes:*1  *- % of positive:*100  *- % of positive & significant:*0  **Functional ability:** Physical domain  *- # of outcomes:*4  *- % of positive:*75  *- % of positive & significant:*50 |
| Robertson et al (2001)  New Zealand (High-income) | **N (randomised):**240  **Inclusion criteria:**  *- Age: ≥ 75years*  **PEDro Score:** 8 | **Type of program:** PA delivered  **Type of PA**: Structured home-based program  **PA classification:** Multicomponent  (strength, walking/wheeling)  **Additional strategies:** Behaviour change strategies (mentoring, telephone calls)  **Length of intervention:** 24 weeks  **Supervised dose (in person):** (12 weeks)  *-Frequency (times/week):* 0.25 (6 visits in 6 months)  *-Duration (min):* 60  **Unsupervised dose**: (12 weeks)  *-Frequency (times/week):* 5  *-Duration (min):* 30  **Prescribed by:** Physiotherapist  **Delivered by:** Trained nurse and self  **Performed with:** Self  **Equipment needed:** Ankle cuffs | **Falls and fall related injuries**  *- # of outcomes:*6  *- % of positive:*83  *- % of positive & significant:*33 |
| Sherrington et al (2014)  Australia (High-income) | **N (randomised):** 340  **Inclusion criteria:**  *- Age:* ≥ 60 years  *- Physical impairment/limitation* (health condition: mixed chronic conditions, recently discharged from orthopaedic wards)  **PEDro Score:** 8 | **Type of program:** PA delivered  **Type of PA**: Structured exercise  **PA classification:** Multicomponent (balance, strength)  **Additional strategies:** Behaviour change strategies (booklet, photos of exercises)  **Length of intervention:** 52 weeks  **Supervised dose (in person):**  *-Frequency (times/week):* 0.2 (10 visits over 12 months)  *-Duration (min):* 25  **Unsupervised dose**:  *-Frequency (times/week):* 3  *-Duration (min):* 25  **Prescribed by:** Physiotherapist  **Delivered by:** Physiotherapist (visits), self  **Performed with:** Self  **Equipment needed:** No | **Physical activity**  *- # of outcomes:2*  *- % of positive:*100  *- % of positive & significant:*50  **Falls and fall related injuries**  *- # of outcomes:*2  *- % of positive:*0  *- % of positive & significant:*0  **Intrinsic capacity:** Physical domain  *- # of outcomes:*2  *- % of positive:*100  *- % of positive & significant:*0  **Functional ability:** Physical domain  *- # of outcomes:*15  *- % of positive:*100  *- % of positive & significant:*27  **Functional ability:** Social domain  *- # of outcomes:*1  *- % of positive:*100  *- % of positive & significant:*100  **Wellbeing and Quality of Life**  *- # of outcomes:*5  *- % of positive:*100  *- % of positive & significant:*20 |
| Sparrow et al (2011)  United States (High-income) | **N (randomised):**103  **Inclusion criteria:**  *- Age:* 50-94 years  **PEDro Score:** 7 | **Type of program:** PA delivered  **Type of PA**: Structured exercise- web based resistance training program  **PA classification:** Strength  **Additional strategies:** Behaviour change strategies (the telecommunications system itself provides monitoring and guidance)  **Length of intervention:** 52 weeks  **Supervised dose (in person):**  *-Frequency (times/week*): 0.1 (1 hands on instruction, 3 visits, 3 phone calls)  *-Duration (min):* 25  **Unsupervised dose**:  *-Frequency (times/week):* 3  *-Duration (min):* 60  **Prescribed by:** Trained staff member  **Delivered by:** Self  **Performed with:** Self  **Equipment needed:** Dumbbells and cuff weights, Telephone Linked Computer based Long-term Interactive Fitness Trainer (TLC-LIFT) provided by staff | **Intrinsic capacity:** Physical domain  *- # of outcomes:*1  *- % of positive:*100  *- % of positive & significant:*100  **Functional ability:** Physical domain  *- # of outcomes:*1  *- % of positive:*100  *- % of positive & significant:*100  **Functional ability:** Emotional and cognitive domain  *- # of outcomes:*1  *- % of positive:*100  *- % of positive & significant:*100 |
| Vogler et al (2009)  Australia (High -income) | **N (randomised):**120  **Inclusion criteria:**  *- Age:* ≥ 65 years  **PEDro Score:** 8 | **Arm A**  **Type of program:** PA delivered  **Type of PA**: Structured exercise  **PA classification:** Strength  **Additional strategies:** No  **Length of intervention:**12 weeks  **Supervised dose (in person):**  *-Frequency (times/week):* 0.67 (8 visits over 12 weeks)  *-Duration (min):* 60  **Unsupervised dose**:  *-Frequency (times/week):* 3  *-Duration (min):* 60  **Prescribed by:** Physiotherapist  **Delivered by:** Physiotherapist, self  **Performed with:** Self  **Equipment needed:** Ankle cuff weights, exercise bands  **Arm B**  **Type of program:** PA delivered  **Type of PA**: Structured exercise  **PA classification:** Multicomponent (Balance, strength)  **Length of intervention:**12 weeks  **Supervised dose (in person):**  *-Frequency (times/week):* 0.67 (8 visits over 12 weeks)  *-Duration (min):* 60  **Unsupervised dose**:  *-Frequency (times/week):* 3  *-Duration (min):* 60  **Prescribed by:** Physiotherapist  **Delivered by:** Physiotherapist, self  **Performed with:** Self  **Equipment needed:** Steps, weight loaded waist belts | **Arm A**  **Functional ability:** Physical domain  *- # of outcomes:*3  *- % of positive:*100  *- % of positive & significant:*0  **Arm B**  **Functional ability:** Physical domain  *- # of outcomes:*3  *- % of positive:*100  *- % of positive & significant:*100 |
| Yang et al (2012)  Australia (High-income) | **N (randomised):**165  **Inclusion criteria:**  *- Age:* ≥ 65 years  *- Physical impairment/limitation:* Yes (falls risk)  **PEDro Score:** 7 | **Type of program:** PA delivered  **Type of PA**: Structured exercise- balance and strength program  **PA classification:** Multicomponent (balance, strength)  **Additional strategies:** Behaviour change strategy (exercise manual)  **Length of intervention:** 26 weeks  **Supervised dose (in person):**  *-Frequency (times/week):* 0.11 (1 visit a week for 3 weeks*)*  *-Duration (min):* 50  **Unsupervised dose**:  *-Frequency (times/week):* 5  *-Duration (min):* 50  **Prescribed by:** Physiotherapist  **Delivered by:** Physiotherapist, self  **Performed with:** Self  **Equipment needed:** Vestibular exercise kit | **Physical activity**  *- # of outcomes:*1  *- % of positive:*100  *- % of positive & significant:*100  **Falls and fall related injuries**  *- # of outcomes:*1  *- % of positive:*100  *- % of positive & significant:*0  **Intrinsic capacity:** Physical domain  *- # of outcomes:*3  *- % of positive:*100  *- % of positive & significant:*33  **Functional ability:** Physical domain  *- # of outcomes:*15  *- % of positive:*80  *- % of positive & significant:*27  **Wellbeing and Quality of Life**  *- # of outcomes:*1  *- % of positive:*100  *- % of positive & significant:*0 |

*Multiple papers from the same study are only included once under a study name

** The list of outcomes presented in this table only includes outcomes investigated in these 22 studies

*** Arm B of this study presented in Table A.7.1 community setting

## Table A.8.3. Description of studies of physical activity interventions for older adults conducted in an outpatient health facility (k=3 studies)

| **Author (Year) or name of study***  **Country (Income level)** | **Study population & PEDro Score** | **Intervention details** | **Effect of reported outcomes** (# of outcomes, % of positive outcomes, % positive & significant outcomes)** |
| --- | --- | --- | --- |
| Barban et al (2017)  Italy, Greece, Spain and Serbia (High-income) | **N (randomised):** 496  **Inclusion criteria:**  *- Age:* ≥65 yrs  *- Physical impairment/limitation:* Yes (Falls risk)  *- Emotional/cognitive status:* Yes (mild cognitive impairment)  **PEDro Score:** 7 | **Arm A**  **Type of program:** PA delivered  **Type of PA**: Structured exercise – motor training  **PA classification:** Multicomponent (balance, strength, wheeling/walking)  **Additional strategies:** No  **Length of intervention:** 12 weeks  **Supervised dose (in person):**  *-Frequency (times/week):* 2  *-Duration (min):* 60  **Prescribed by:** Therapist  **Delivered by:** Therapist  **Performed with:** Mixed  **Equipment needed**: i-walker (assistive technology device developed to support users with mobility disturbances by compensating for unbalanced muscle force and lack of muscle)  **Arm B**  **Type of program:** PA delivered  **Type of PA**: Structured exercise – motor and cognitive training combined  **PA classification:** Multicomponent (balance, strength, wheeling/walking)  **Additional strategies:** No  **Length of intervention:** 12 weeks  **Supervised dose (in person):**  *-Frequency (times/week):* 2  *-Duration (min):* 30 (30min motor 30min cognitive)  **Prescribed by:** Therapist and cognitive therapist  **Delivered by:** Therapist and cognitive therapist  **Performed with:** Mixed  **Equipment needed:** i-walker (as above) | **Arm A**  **Functional ability:** Emotional & cognitive domain  *- # of outcomes:*1  *- % of positive:*100  *- % of positive & significant:*100  **Arm B**  **Functional ability:** Emotional & cognitive domain  *- # of outcomes:*1  *- % of positive:*100  *- % of positive & significant:*100 |
| Martin-Borras et al (2018)  Spain (High-income) | **N (randomised):** 422  **Inclusion criteria:**  *- Age:* 18-85  *- Physical impairment/limitation:* Yes (low PA levels, at least one chronic condition)  **PEDro Score:** 7 | **Type of program:** PA delivered  **Type of PA**: Structured exercise  **PA classification:** Multicomponent (strength, endurance)  **Additional strategies:** Behaviour change strategies (discussion around health belief/attitudes and behaviour change)  **Length of intervention:** 12  **Supervised dose (in person):**  *-Frequency (times/week):*2  *-Duration (min):* 60  **Unsupervised dose**:  *-Frequency (times/week):* 1  *-Duration (min):* 30  **Prescribed by:** PA specialist  **Delivered by:** PA specialist  **Performed with:** Group  **Equipment needed:** Elastic bands | **Physical activity**  *- # of outcomes:*4  *- % of positive:*75  *- % of positive & significant:*75 |
| Sherrington et al (2008)  Australia (High-income) | **N (randomised):** 173  **Inclusion criteria:**  *- Age:* ≥65 yrs  *- Physical impairment/limitation:* Yes (low mobility)  **PEDro Score:** 7 | **Type of program:** PA delivered  **Type of PA**: Structured exercise  **PA classification:** Multicomponent (balance, walking/wheeling)  **Additional strategies:** No  **Length of intervention:** 5 weeks  **Supervised dose (in person):**  *-Frequency (times/week):*2  *-Duration (min):*60  **Unsupervised dose**:  *-Frequency (times/week):*5  *-Duration (min):*60  **Prescribed by:** Physiotherapist  **Delivered by:** Physiotherapists  **Performed with:** Group  **Equipment needed:** No | **Intrinsic capacity:** Physical domain  *- # of outcomes:*4  *- % of positive:*25  *- % of positive & significant:*0  **Functional ability**: Physical domain  *- # of outcomes:*9  *- % of positive:*100  *- % of positive & significant:* 33 |

*Multiple papers from the same study are only included once under a study name

** The list of outcomes presented in this table only includes outcomes investigated in these three studies

## Table A.8.4. Description of studies of physical activity interventions for older adults conducted in a residential aged care facility (k=8 studies)

| **Author (Year) or name of study* Country (Income level)** | **Study population & PEDro Score** | **Intervention details** | **Effect of reported outcomes** (# of outcomes, % of positive outcomes, % positive & significant outcomes)** |
| --- | --- | --- | --- |
| FOPANU study  Sweden (High-income)  Carlsson et al (2011)  Conradsson et al (2010)  Rosendahl et al (2006)  Rosendahl et al (2008) | **N (randomised):** 191  **Inclusion criteria:**  *- Age* ≥65 years  *- Physical impairment/limitation:* Yes (difficulty walking/slow) walking/limited distance  *- Emotional/cognitive status:* Yes (mild cognitive impairment)  **PEDro Score:**  7  8  8  7 | **Type of program:** PA delivered  **Type of PA**: Structured exercise – high intensity functional exercise program  **PA classification:** Multicomponent (Balance/functional/neuromotor, strength/resistance/power)  **Additional strategies:** No  **Length of intervention:** 13 weeks  **Supervised dose (in person):**  *-Frequency (times/week):* 2.5 (5 times every 2 weeks)  *-Duration (min):* 45 min  **Prescribed by:** Physiotherapist  **Delivered by:** Physiotherapist  **Performed with:** Group  **Equipment needed:** Yes (weighted belt with up to 12 kg) | **Falls and fall related injuries**  *- # of outcomes:*2  *- % of positive:*100  *- % of positive & significant:*0  **Intrinsic capacity:** Physical domain  *- # of outcomes:*3  *- % of positive:*100  *- % of positive & significant:*33  **Functional ability:** Physical domain  *- # of outcomes:*5  *- % of positive:*80  *- % of positive & significant:*20  **Functional ability:** Emotional & cognitive domain  *- # of outcomes*:3  *- % of positive:*67  *- % of positive & significant:*0 |
| Dutch randomised controlled exercise trial in long-term care facilities (no name)  Netherlands (High-income)  Chin A Paw et al (2004),  Chin A Paw et al (2006) | **N (randomised):**  108 (arm A)  111 (arm B)  107 (arm C)  **Inclusion criteria:**  *- Age:* $\geq$ 65  *- Physical impairment/limitation:* no  *- Emotional/cognitive status:* no  **PEDro Score:** 7  **PEDro Score:** 6 | **Arm A**  **Type of program:** PA delivered  **Type of PA**: Structured exercise – resistance training  **PA classification:** Strength  **Additional strategies:** No  **Length of intervention:** 26 weeks  **Supervised dose (in person):**  *-Frequency (times/week):* 2  *-Duration (min):* 53  **Prescribed by:** primary researcher  **Delivered by:** Physical therapist and assistant  **Performed with:** Group  **Equipment needed:** Yes (Technogym equipment, dumb bells, ankle, wrist weights)  **Arm B**  **Type of program:** PA delivered  **Type of PA**: Structured exercise – all round functional skills training (including game-like activities such as musical chairs and team pursuit races)  **PA classification:** Balance  **Additional strategies:** No  **Length of intervention:** 26 weeks  **Supervised dose (in person):**  *-Frequency (times/week*): 2  *-Duration (min):* 48  **Prescribed by:** Primary researcher  **Delivered by:** Physical therapist and assistant  **Performed with:** Group  **Equipment needed:** Yes (balls, wrist and ankle weights)  **Arm C**  **Type of program:** PA delivered  **Type of PA**: Structured exercise - combination of A and B above  **PA classification:** Multicomponent (balance and strength)  **Additional strategies:** No  **Length of intervention:** 26  **Supervised dose (in person):**  *-Frequency (times/week):* 2  *-Duration (min):* 50  **Prescribed by:** Primary researcher  **Delivered by:** Physical therapist and assistant  **Performed with:** Group  **Equipment needed:** Yes (Technogym equipment, dumbells, wrist and ankle weights, balls) | **Arm A**  **Intrinsic capacity:** Physical domain  *- # of outcomes:*3  *- % of positive:*0  *- % of positive & significant:*0  **Functional ability:** Physical domain  *- # of outcomes:*7  *- % of positive:*14  *- % of positive & significant:*0  **Functional ability:** Emotional & cognitive domain  *- # of outcomes:*10  *- % of positive:*10  *- % of positive & significant:*0  **Wellbeing & QoL**  *- # of outcomes:*1  *- % of positive:*0  *- % of positive & significant:*0  **Arm B**  **Intrinsic capacity:** Physical domain  *- # of outcomes:*3  *- % of positive:*33  *- % of positive & significant:*0  **Functional ability:** Physical domain  *- # of outcomes:*7  *- % of positive:*14  *- % of positive & significant:*0  **Functional ability:** Emotional & cognitive domain  *- # of outcomes:*10  *- % of positive:*9  *- % of positive & significant:*0  **Wellbeing & QoL**  *- # of outcomes:* 1  *- % of positive:* 0  *- % of positive & significant:* 0  **Arm C**  **Intrinsic capacity:** Physical domain  *- # of outcomes:* 3  *- % of positive:* 0  *- % of positive & significant:* 0  **Functional ability:** Physical domain  *- # of outcomes:*7  *- % of positive:*14  *- % of positive & significant:* 0  **Functional ability: Emotional & cognitive domain**  *- # of outcomes:*10  *- % of positive:*10  *- % of positive & significant:* 0  **Wellbeing & QoL**  *- # of outcomes:*1  *- % of positive*:0  *- % of positive & significant:0* |
| Faber et al (2006)  The Netherlands (High-income) | **N (randomised):**  184 (arm A)  198 (arm B)  **Inclusion criteria:**  *- Age* $\geq$80  *- Physical impairment/limitation:* no  *- Emotional/cognitive status:* no  **PEDro Score:** 6 | **Arm A**  **Type of program:** PA delivered  **Type of PA**: Structured exercise (functional and balance exercises)  **PA classification:** Balance, function  **Additional strategies:** No  **Length of intervention:** 20 weeks  **Supervised dose (in person):**  *-Frequency (times/week):* 1.8 (initially 1x week, then 2)  *-Duration (min):* 60  **Prescribed by:** Trained instructors  **Delivered by:** Trained instructors  **Performed with:** Group  **Equipment needed:** Yes (balls, weighted objects, obstacles, steps)  **Arm B**  **Type of program:** PA delivered  **Type of PA**: Structured exercise – Tai Chi type balance exercises  **PA classification:** Balance, function  **Additional strategies:** No  **Length of intervention:** 20 weeks  **Supervised dose (in person):**  *-Frequency (times/week):* 1.8 (initially 1x week, then 2)  *-Duration (min):* 60  **Prescribed by:** trained instructors  **Delivered by:** trained instructors  **Performed with:** Group  **Equipment needed:** yes (obstacles) | **Arm A**  **Functional ability:** Physical domain  *- # of outcomes:* 3  *- % of positive:* 100  *- % of positive & significant:* 67  **Arm B**  **Functional ability:** Physical domain  *- # of outcomes:3*  *- % of positive:100*  *- % of positive & significant:0* |
| Hewitt et al (2018)  Australia (High-income) | **N (randomised):** 221  **Inclusion criteria:**  *- Age:* $\geq$ 65  *- Physical impairment/limitation:* yes (low PA levels)  *- Emotional/cognitive status:* no  **PEDro Score:** 8 | **Type of program:** PA delivered  **Type of PA**: Structured exercise- The Sunbeam program - progressive resistance training plus balance exercise  **PA classification:** Multicomponent **(**balance, strength)  **Additional strategies:** None  **Length of intervention:** 25 weeks  **Supervised dose (in person):**  *-Frequency (times/week):* 2  *-Duration (min):* 60  **Prescribed by:** Physiotherapist and activity officer  **Delivered by:** Physiotherapist and activity officer  **Performed with:** Group  **Equipment needed:** Pneumatic resistance equipment | **Falls and fall related injuries**  *- # of outcomes:*7  *- % of positive:*100  *- % of positive & significant:*29  **Functional ability:** Physical domain  *- # of outcomes:3*  *- % of positive:100*  *- % of positive & significant:67* |
| Machacova et al (2017)  Czech Republic (High-income) | **N (randomised):** 189  **Inclusion criteria:**  *- Age:* ≥60 yrs  *- Physical impairment/limitation:* no  *- Emotional/cognitive status*: no  **PEDro Score:** 6 | **Type of program:** PA delivered  **Type of PA**: Recreation - dance  **PA classification:** Dance based program  **Additional strategies:** No  **Length of intervention:** 12  **Supervised dose (in person):**  *-Frequency (times/week):* 1  *-Duration (min):* 60  **Prescribed by:** Dance instructor  **Delivered by:** Dance instructor  **Performed with:** Group  **Equipment needed:** No | **Functional ability:** Physical domain  *- # of outcomes:*2  *- % of positive:*100  *- % of positive & significant:*100 |
| Mulrow et al (1994)  United States (High-income) | **N (randomised):** 194  **Inclusion criteria:**  *- Age**:* ≥60 yrs  *Physical impairment/limitation:* yes (low mobility)  *- Emotional/cognitive status:* no  **PEDro Score:** 6 | **Type of program:** PA delivered  **Type of PA**: Structured exercise  **PA classification:** Multicomponent (balance, strength, and endurance)  **Additional strategies:** No  **Length of intervention:** 17 weeks  **Supervised dose (in person):**  *-Frequency (times/week):* 3  *-Duration (min):* 45  **Prescribed by:** Physiotherapist  **Delivered by:** One of six PTs  **Performed with:** Self  **Equipment needed:** Cuff weights or elastic bands | **Intrinsic capacity:** Physical domain  *- # of outcomes:1*  *- % of positive:*100  *- % of positive & significant:*0  **Functional ability:** Physical domain  *- # of outcomes:*4  *- % of positive:*50  *- % of positive & significant:*25  **Functional ability:** Emotional & cognitive domain  *- # of outcomes:*2  *- % of positive:*0  *- % of positive & significant:*0  **Wellbeing & QoL**  *- # of outcomes:*1  *- % of positive:* 0  *- % of positive & significant:* 0 |
| Peri et al (2008)  New Zealand (High-income) | **N (randomised):**149  **Inclusion criteria:**  *- Age:* ≥65 yrs  *- Physical impairment/limitation:* yes (low mobility)  *- Emotional/cognitive status:* no  **PEDro Score:** 7 | **Type of program:** PA delivered  **Type of PA**: Structured exercise (repetitive ADL activity program)  **PA classification:** Endurance  **Additional strategies:** Compliance recording sheet  **Length of intervention:** 26 weeks  **Supervised dose (in person):**  *-Frequency (times/week):* Individualised  *-Duration (min):* Individualised  **Prescribed by:** Gerontology research nurse  **Delivered by:** Usual caregivers  **Performed with:** Self  **Equipment needed:** No | **Falls and fall related injuries**  *- # of outcomes:*1  *- % of positive:*100  *- % of positive & significant:*0  **Functional ability:** Physical domain  *- # of outcomes:*3  *- % of positive:*67  *- % of positive & significant:*33  **Functional ability:** Emotional & cognitive domain  *- # of outcomes:*1  *- % of positive:*0  *- % of positive & significant:*0  **Wellbeing & QoL**  *- # of outcomes:*1  *- % of positive:* 0  *- % of positive & significant:* 0 |
| Tsang et al (2013)  Hong Kong (High-income) | **N (randomised):**  **Inclusion criteria:**  *- Age:* ≥60 yrs  *- Physical impairment/limitation:* yes (frailty)  *- Emotional/cognitive status:* no  **PEDro Score:** 8 | **Type of program:** PA delivered  **Type of PA**: Recreation  **PA classification:** Tai Chi (Qigong)  **Additional strategies:** No  **Length of intervention:** 12  **Supervised dose (in person):**  *-Frequency (times/week):*2  *-Duration (min):* 60  **Unsupervised dose**:  *-Frequency (times/week):* 5  *-Duration (min):* 60  **Prescribed by:** Qigong instructors  **Delivered by:** Qigong instructors  **Performed with:** Group  **Equipment needed:** No | **Intrinsic capacity**: Physical domain  *- # of outcomes:* 4  *- % of positive:* 50  *- % of positive & significant:*0  **Functional ability:** Physical domain  *- # of outcomes:*1  *- % of positive:* 100  *- % of positive & significant:* 0  **Functional ability:** Emotional & cognitive domain  *- # of outcomes:* 8  *- % of positive:* 63  *- % of positive & significant:* 13 |

*Multiple papers from the same study are only included once under a study name

** The list of outcomes presented in this table only includes outcomes investigated in these eight studies

## Table A.8.5. Description of studies of physical activity interventions for older adults conducted in a retirement village (k=4 studies)

| **Author (Year) or name of study***  **Country (Income level)** | **Study population & PEDro Score** | **Intervention details** | **Effect of reported outcomes**(# of outcomes, % of positive outcomes, % positive & significant outcomes)** |
| --- | --- | --- | --- |
| Intense Tai Chi, Atlanta study  United States (High-income)  Greenspan (2007)  Wolf (2003)  Sattin (2005)  Wolf (2006) | **N (randomised):** 291 (Greenspan et al), 311 (Wolf 2003, Sattin, and Wolf 2006)  **Inclusion criteria:**  *- Age:* ≥70 years  *-Gender*: Any  *- Physical impairment/limitation:* Yes (frailty)  *- Emotional/cognitive status*: No  **PEDro Score:** 6 | **Type of program:** PA delivered  **Type of PA**: Recreation  **PA classification:** Tai Chi  **Additional strategies:** No  **Length of intervention:** 48 weeks  **Supervised dose (in person):**  *-Frequency (times/week):* 2  *-Duration (min):* 75  **Unsupervised dose:**  *-Frequency (times/week):* 4.5  *-Duration (min):* 20  **Delivered by:** Tai Chi instructor  **Performed with:** Group  **Equipment needed:** No | **Falls and fall related injuries**  *- # of outcomes:* 1  *- % of positive:* 100  *- % of positive & significant:* 0  **Intrinsic capacity**: Physical domain  *- # of outcomes:* 4  *- % of positive:* 100  *- % of positive & significant:* 100  **Functional ability**: Physical domain  *- # of outcomes:* 5  *- % of positive:* 100  *- % of positive & significant:* 60 |
| Lord et al (2003)  Australia (High-income) | **N (randomised):** 551  **Inclusion criteria:**  *- Age:* ≥60 years  *-Gender*: Any  *- Physical impairment/limitation:* Yes (frailty)  *- Emotional/cognitive status*: No  **PEDro Score:** 6 | **Type of program:** PA delivered  **Type of PA**: Structured exercise  **PA classification:** Multicomponent (balance, strength, walking/wheeling  **Additional strategies:** No  **Length of intervention:** 52 weeks  **Supervised dose (in person):**  *-Frequency (times/week):*  *-Duration (min):* 60  **Prescribed by:** Health educator  **Delivered by:** Instructors  **Performed with:** Group  **Equipment needed:** No | **Falls and fall related injuries**  *- # of outcomes:* 1  *- % of positive:* 100  *- % of positive & significant:* 100  **Intrinsic capacity**: Physical domain  *- # of outcomes:* 1  *- % of positive:* 0  *- % of positive & significant:* 0  **Functional ability:** Physical domain  *- # of outcomes:* 8  *- % of positive:* 50  *- % of positive & significant:* 2 |
| Merom et al (2016)  Australia (High-income) | **N (randomised):** 530  **Inclusion criteria:**  *- Age:* ≥80 years  *-Gender*: Female  *- Physical impairment/limitation:* No  *- Emotional/cognitive status*: No  **PEDro Score:** 8 | **Type of program:** PA delivered  **Type of PA**: Recreation  **PA classification:** Dance  **Additional strategies:** No  **Length of intervention:** 12 weeks  **Supervised dose (in person):**  *-Frequency (times/week):* 2  *-Duration (min):* 60  **Delivered by:** Dance teacher  **Performed with:** Group  **Equipment needed:** No | **Physical activity**  *- # of outcomes:* 3  *- % of positive:* 67  *- % of positive & significant:* 67  **Falls and fall related injuries**  *- # of outcomes:* 1  *- % of positive:* 0  *- % of positive & significant:* 0  **Intrinsic capacity**: Physical domain  *- # of outcomes:* 1  *- % of positive:* 0  *- % of positive & significant:* 0  **Functional ability**: Physical domain  *- # of outcomes:* 6  *- % of positive:* 33  *- % of positive & significant:* 0  **Functional ability:** Emotional & Cognitive domain  *- # of outcomes:* 3  *- % of positive:* 100  *- % of positive & significant:* 0  **Wellbeing & QoL**  *- # of outcomes:* 2  *- % of positive:* 0  *- % of positive & significant:* 0 |
| Tajik et al (2018)  Iran (Upper-middle income) | **N (randomised):** 132  **Inclusion criteria:**  *- Age:* ≥60 years  *-Gender*: Any  *- Physical impairment/limitation:* No  *- Emotional/cognitive status*: No  **PEDro Score:** 8 | **Type of program:** PA delivered  **Type of PA**: Recreation  **PA classification:** Tai Chi  **Additional strategies:** No  **Length of intervention:** 8 weeks  **Supervised dose (in person):**  *-Frequency (times/week):* 3  *-Duration (min):* 35  **Delivered by:** Researcher trained in Tai Chi  **Performed with:** Group  **Equipment needed:** No | **Physical domain:** Functional ability  *- # of outcomes:* 2  *- % of positive:* 100  *- % of positive & significant:* 100  **Functional ability:** Social domain  *- # of outcomes:* 1  *- % of positive:* 100  *- % of positive & significant:* 100  **Functional ability**: Emotional & Cognitive domain  *- # of outcomes:* 2  *- % of positive:* 100  *- % of positive & significant:* 100  **Wellbeing & QoL**  *- # of outcomes:* 1  *- % of positive:* 0  *- % of positive & significant:* 0 |

* Multiple papers from the same study are only included once under a study name

**The list of outcomes presented in this table only includes outcomes investigated in these four studies

## Table A.8.6. Description of study of physical activity interventions for older adults conducted in no set location (k=14 studies)

| **Author (Year) or name of study***  **Country (Income level)** | **Study population & PEDro Score** | **Intervention details** | **Effect of reported outcomes**(# of outcomes, % of positive outcomes, % positive & significant outcomes)** |
| --- | --- | --- | --- |
| DirectLife program study  Netherlands (High-income)  (2 papers)  Broekhuizen et al (2016)  Wijsman et al (2013) | **N (randomised):** 235  **Inclusion criteria:**  *-Age:* ≥ 60years  *- Gender*: Any  *- Physical impairment/limitation:* No  *- Emotional/cognitive status:* No  **PEDro Score:** 8 | **Type of program:** PA promoted  **Type of PA**: Overall activity  **PA classification:** Coaching  **Additional strategies:** Behaviour change strategy (activity monitor)  **Length of intervention:** 12 weeks  **Unsupervised dose:**  *-Frequency (times/week:* 7  *-Duration (min):* 30  **Delivered by:** e-coach  **Performed with:** Self  **Equipment needed:** Yes (activity monitor, electronic device) | **Physical activity**  *- # of outcomes:* 3  *- % of positive:*100  *- % of positive & significant:*67  **Intrinsic capacity**: Physical domain  *- # of outcomes: 22*  *- % of positive:* 95  *- % of positive & significant:* 27  **Functional ability:** Physical domain  *- # of outcomes:* 2  *- % of positive:* 50  *- % of positive & significant:* 0  **Functional ability:** Social domain  *- # of outcomes:* 1  *- % of positive:* 100  *- % of positive & significant:* 0  **Functional ability:** Emotional & cognitive domain  *- # of outcomes:* 2  *- % of positive:* 100  *- % of positive & significant:* 50  **Wellbeing & QoL**  *- # of outcomes:* 2  *- % of positive:* 50  *- % of positive & significant:* 50 |
| Herghelegiu et al (2007)  Romania (High-income) | **N (randomised):** 200  **Inclusion criteria:**  *-Age:* ≥65 years  *- Gender*: Any  *- Physical impairment/limitation:* Yes (frailty)  *- Emotional/cognitive status:* No  **PEDro Score:** 6 | **Type of program:** PA promoted  **Type of PA**: Overall activity  **PA classification:** Other  **Additional strategies:** Behaviour change strategies (mentoring, counselling session)  **Length of intervention:** 26 weeks  **Supervised dose (in person):**  *-Frequency (times/week:* 0.23  *-Duration (min):* 7  **Unsupervised dose:** 26 weeks  *-Frequency (times/week:* Individualised  *-Duration (min):* Individualised  **Delivered by:** Geriatrician/health counsellor  **Performed with:** Self  **Equipment needed:** No | **Physical activity**  *- # of outcomes:* 6  *- % of positive:* 100  *- % of positive & significant:* 83 |
| Kerse et al (2005)  New Zealand (High-income) | **N (randomised):** 878  **Inclusion criteria:**  *-Age:* ≥65years  *- Gender*: Any  *- Physical impairment/limitation:* No  *- Emotional/cognitive status:* No  **PEDro Score:** 6 | **Type of program:** PA promoted  **Type of PA**: Overall activity  **PA classification:** Other (participants received individualized advice from their primary care doctor, who then referred them to exercise specialists)  **Additional strategies:** Behaviour change strategy (activity counselling)  **Length of intervention:** 52 weeks  **Supervised dose (in person and by phone):**  *-Frequency (times/week:* 0.1 (1 face to face consultation with primary care doctor and 3 follow up phone calls over a 12-month period)  *-Duration (min):* 30  **Unsupervised dose:** 52 weeks  *-Frequency (times/week:* Individualised  *-Duration (min):* Individualised  **Delivered by:** Primary care doctor, exercise specialists  **Performed with:** Self  **Equipment needed:** No | **Physical activity**  *- # of outcomes:* 3  *- % of positive:* 100  *- % of positive & significant:* 67  **Intrinsic capacity:** Physical domain  *- # of outcomes:* 3  *- % of positive:* 100  *- % of positive & significant:* 0  **Functional ability:** Physical domain  *- # of outcomes:* 2  *- % of positive:* 100  *- % of positive & significant:* 0  **Functional ability:** Social domain  *- # of outcomes:* 1  *- % of positive:* 100  *- % of positive & significant:* 0  **Functional ability:** Emotional & cognitive domain  *- # of outcomes:* 2  *- % of positive:* 50  *- % of positive & significant:* 0 |
| King et al (2007)  United States (High-income) | **N (randomised):**143  **Inclusion criteria:**  *-Age:* ≥ 50years  *- Gender*: Any  *- Physical impairment/limitation:* No  *- Emotional/cognitive status:* No  **PEDro Score:** 7 | **Arm A**  **Type of program:** PA promoted  **Type of PA**: Overall activity  **PA classification:** Coaching  **Additional strategies:** Behaviour change strategies (activity monitor, mentoring)  **Length of intervention:** 52 weeks  **Supervised dose (live online):**  *-Frequency (times/week:* 5  *-Duration (min):* 30  **Prescribed by:** Study team  **Delivered by:** Trained health educator  **Performed with:** Self  **Equipment needed:** No  **Arm B**  As above apart from:  **Delivered by:** computer controlled interactive telephone system | **Arm A**  **Physical activity**  *- # of outcomes:* 6  *- % of positive:*100  *- % of positive & significant:*100  **Functional ability:** Physical domain  *- # of outcomes:*1  *- % of positive:* 100  *- % of positive & significant:* 100  **Wellbeing & QoL**  *- # of outcomes:* 1  *- % of positive:* 100  *- % of positive & significant:* 100  **Arm B**  **Physical activity**  *- # of outcomes:* 6  *- % of positive:*100  *- % of positive & significant:* 83  **Functional ability:** Physical domain  *- # of outcomes:*1  *- % of positive:* 100  *- % of positive & significant:* 0  **Wellbeing & QoL**  *- # of outcomes:* 1  *- % of positive:* 100  *- % of positive & significant:* 0 |
| Kolt et al (2007)  New Zealand (High-income) | **N (randomised):** 186  **Inclusion criteria:**  *-Age:* ≥65 years  *- Gender*: Any  *- Physical impairment/limitation:* No  *- Emotional/cognitive status:* No  **PEDro Score:** 6 | **Type of program:** PA promoted  **Type of PA**: Overall activity  **PA classification:** Coaching (Telephone based counselling interview)  **Additional strategies:** Behaviour change strategy (telephone- based counselling)  **Length of intervention:** 12 weeks  **Supervised dose (by phone):**  *-Frequency (times/week):* 0.66  *-Duration (min):* 15  **Prescribed by:** Primary care physician  **Delivered by:** Exercise counsellor  **Performed with: S**elf  **Equipment needed:** No | **Physical activity**  *- # of outcomes:*2  *- % of positive:*100  *- % of positive & significant:*100 |
| Komulainen et al (2007)  Finland (High-income) | **N (randomised):** 468  **Inclusion criteria:**  *-Age:* ≥50 years  *- Gender*: Any  *- Physical impairment/limitation:* No  *- Emotional/cognitive status:* No  **PEDro Score:** currently being rated | **Type of program:** PA promoted  **Type of PA**: Overall activity  **PA classification:** Other (training written on a personal smart card)  **Additional strategies:** Behaviour change strategy (face-to-face counselling sessions)  **Length of intervention:** 104 weeks  **Unsupervised dose:**  *-Frequency (times/week):*5  *-Duration (min):* 60  **Prescribed by:** Exercise physiologist  **Delivered by:** Self  **Performed with:** Self  **Equipment needed:** No | **Physical activity**  *- # of outcomes:*3  *- % of positive:*100  *- % of positive & significant:* 67  **Functional ability:** Emotional & cognitive domain  *- # of outcomes:* 7  *- % of positive:* 43  *- % of positive & significant:* 0 |
| McMurdo et al (2011)  Scotland (High-income) | **N (randomised):**136  **Inclusion criteria:**  *-Age:* ≥ 70years  *- Gender*: female  *- Physical impairment/limitation:* Yes (Low PA levels)  *- Emotional/cognitive status:* No  **PEDro Score:** 7 | **Arm A**  **Type of program:** PA promoted – brief/very brief intervention  **Type of PA**: Overall activity  **PA classification:** Walking/wheeling  **Additional strategies:** Behaviour change strategies (incentives, telephone calls)  **Length of intervention:** 26 weeks  **Supervised dose (by phone):**  *-Frequency (times/week):* 0.42 (contacted by phone once a week first month, every second week for 2 months then monthly)  *-Duration (min):* Individualised  **Prescribed by:** Health psychologist  **Delivered by:** Self  **Performed with:** Self  **Equipment needed:** No  **Arm B**  **Type of program:** PA promoted - brief/very brief intervention  **Type of PA**: Overall activity  **PA classification:** walking/wheeling with pedometer  **Additional strategies:** Behaviour change strategies (incentives, activity monitor, telephone calls)  **Length of intervention:** 26 weeks  **Supervised dose (in person):**  *-Frequency (times/week):* 0.42 (contacted by phone once a week first month, every second week for 2 months then monthly)  *-Duration (min):* Individualised  **Prescribed by:** Health psychologist  **Delivered by:** Self  **Performed with:** Self  **Equipment needed:** Pedometer | **Arm A**  **Physical activity**  *- # of outcomes:*2  *- % of positive:*100  *- % of positive & significant:*100  **Functional ability:** Physical domain  *- # of outcomes:*1  *- % of positive:* 0  *- % of positive & significant:*0  **Arm B**  **Physical activity**  *- # of outcomes:*2  *- % of positive:*100  *- % of positive & significant:*100  **Functional ability:** Physical domain  *- # of outcomes:*1  *- % of positive:*100  *- % of positive & significant:*0 |
| Morey et al (2006)  United States (High-income) | **N (randomised):**132  **Inclusion criteria:**  *-Age:* ≥ 70years  *- Gender*: male  *- Physical impairment/limitation:* No  *- Emotional/cognitive status:* No  **PEDro Score:** 6 | **Type of program:** PA promoted  **Type of PA**: Overall activity  **PA classification:** Coaching  **Additional strategies:** Behaviour change strategies (activity monitor, mentoring)  **Length of intervention:** 26 weeks  **Unsupervised dose:**  *-Frequency (times/week):* 5  *-Duration (min):* 30  **Delivered by:** Health counsellor  **Performed with:** Self  **Equipment needed:** No | **Physical activity**  *- # of outcomes:*2  *- % of positive:*100  *- % of positive & significant:*0 |
| Morey et al (2009)  United States (High-income) | **N (randomised):** 398  **Inclusion criteria:**  *-Age:* ≥70years  *- Gender*: Male  *- Physical impairment/limitation:* No  *- Emotional/cognitive status:* No  **PEDro Score:** 7 | **Type of program:** PA promoted  **Type of PA**: Overall activity  **PA classification:** Coaching  **Additional strategies:** Behaviour change strategies (activity monitor, telephone counselling, provider messaging, individually tailored progress report)  **Length of intervention:** 52 weeks  **Unsupervised dose:**  *-Frequency (times/week):* 5  *-Duration (min):* 45  **Prescribed by:** Life-style counsellor, primary care provider **Delivered by:** Self, life-style counsellor, primary care provider  **Performed with:** Self  **Equipment needed:** Yes (gym equipment, elastic bands) | **Physical activity**  *- # of outcomes:* 3  *- % of positive:*100  *- % of positive & significant:* 100  **Physical domain:** Intrinsic capacity  *- # of outcomes:* 2  *- % of positive:* 50  *- % of positive & significant:* 0  **Functional ability:** Physical domain  *- # of outcomes:* 6  *- % of positive:* 83  *- % of positive & significant:* 17  **Functional ability:** Social domain  *- # of outcomes:* 2  *- % of positive:* 100  *- % of positive & significant:* 50 |
| Oliveira et al (2019)  Australia (High-income) | **N (randomised):**131  **Inclusion criteria:**  *-Age:* ≥60years  *- Gender*: Any  *- Physical impairment/limitation:* No  *- Emotional/cognitive status:* No  **PEDro Score:** 8 | **Type of program:** PA promoted  **Type of PA**: Overall activity  **PA classification:** Coaching  **Additional strategies:** Behaviour change strategy (activity monitor)  **Length of intervention:** 26 weeks  **Unsupervised dose:**  *-Frequency (times/week):* Individualised  *-Duration (min):* Individualised  **Delivered by:** Physiotherapist  **Performed with:** Self  **Equipment needed:** No | **Physical activity**  *- # of outcomes:* 2  *- % of positive:*100  *- % of positive & significant:* 0  **Falls and fall related injuries**  *- # of outcomes:* 2  *- % of positive:* 0  *- % of positive & significant:* 0  **Functional ability:** Physical domain  *- # of outcomes:* 2  *- % of positive:* 0  *- % of positive & significant:* 0  **Functional ability:** Social domain  *- # of outcomes:* 2  *- % of positive:* 50  *- % of positive & significant:* 0  **Functional ability:** Emotional & cognitive domain  *- # of outcomes:* 2  *- % of positive:* 50  *- % of positive & significant:* 0  **Wellbeing & QoL**  *- # of outcomes:* 1  *- % of positive:* 100  *- % of positive & significant:* 0 |
| SCAMOB Study  Finland (High-income)  (3 papers)  Manty et al (2009)  Rasinaho et al (2012)  Von Bonsdorff et al (2008) | **N (randomised):** 632  **Inclusion criteria:**  *-Age:* ≥75 years  *- Gender*: Any  *- Physical impairment/limitation:* No  *- Emotional/cognitive status:* No  **PEDro Score:** 7 | **Type of program:** PA promoted  **Type of PA**: Overall activity  **PA classification:** Coaching  **Additional strategies:** Behaviour change strategy (mentoring)  **Length of intervention:** 104 weeks  **Supervised dose (in person):**  *-Frequency (times/week):* 0.01 (one face to face session)  *-Duration (min):* 60  **Unsupervised dose:** 104 weeks  *-Frequency (times/week):* Individualised  *-Duration (min):* Individualised  **Delivered by:** Physiotherapist  **Performed with:** Self  **Equipment needed:** No | **Physical activity**  *- # of outcomes*: 7  *- % of positive:* 100  *- % of positive & significant:*57  **Functional ability:** Physical domain  *- # of outcomes:* 3  *- % of positive:* 100  *- % of positive & significant:* 33 |
| Thomas et al (2012)  Hong Kong (High-income) | **N (randomised):**  Arm A 397  Arm B 410  Arm C 388  **Inclusion criteria:**  *-Age:* ≥60 years  *- Gender*: Any  *- Physical impairment/limitation:* No  *- Emotional/cognitive status:* No  **PEDro Score: 7** | **Arm A –** pedometer and buddy  **Type of program:** PA promoted  **Type of PA**: Overall activity  **PA classification:** Coaching  **Additional strategies:** Behaviour change strategies (activity monitor, telephone calls)  **Length of intervention:** 52 weeks  **Unsupervised dose:**  *-Frequency (times/week):* 4  *-Duration (min):* 45  **Prescribed by:** Study team  **Delivered by:** Self  **Performed with:** Self and one other person  **Equipment needed:** No  **Arm B (**pedometer only)  **Type of program:** PA promoted  **Type of PA**: Overall activity  **PA classification:** Coaching  **Additional strategies:** Behaviour change strategies (activity monitor, telephone calls)  **Length of intervention:** 52 weeks  **Unsupervised dose:**  *-Frequency (times/week):* 4  *-Duration (min):* 45  **Prescribed by:** Study team  **Delivered by:** Self  **Performed with:** Self  **Equipment needed:** No  **Arm C (**buddy only)  **Type of program:** PA promoted  **Type of PA**: Overall activity  **PA classification:** Coaching  **Additional strategies:** Behaviour change strategies  (telephone calls)  **Length of intervention:** 52 weeks  **Unsupervised dose:**  *-Frequency (times/week):* 4  *-Duration (min):* 30  **Prescribed by:** Study team  **Delivered by:** Self  **Performed with:** Self and one other person  **Equipment needed:** No | **Arm A**  **Physical activity**  *- # of outcomes:* 1  *- % of positive:* 100  *- % of positive & significant:* 100  **Intrinsic capacity:** Physical domain  *- # of outcomes:* 2  *- % of positive:* 100  *- % of positive & significant:* 0  **Arm B**  **Physical activity**  *- # of outcomes:* 1  *- % of positive:* 100  *- % of positive & significant:* 100  **Intrinsic capacity:** Physical domain  *- # of outcomes:* 10  *- % of positive:* 90  *- % of positive & significant:* 0  **Functional ability:** Physical domain  *- # of outcomes:* 2  *- % of positive:* 0  *- % of positive & significant:* 0  **Arm C**  **Physical activity**  *- # of outcomes:* 1  *- % of positive:* 100  *- % of positive & significant:* 100  **Intrinsic capacity:** Physical domain  *- # of outcomes:* 10  *- % of positive:* 90  *- % of positive & significant:* 30  **Functional ability:** Physical domain  *- # of outcomes:* 2  *- % of positive:* 100  *- % of positive & significant:* 50 |
| Voukelatos et al (2015)  Australia (High-income) | **N (randomised):** 386  **Inclusion criteria:**  *-Age:* ≥65years  *- Gender*: Any  *- Physical impairment/limitation:* No  *- Emotional/cognitive status:* No  **PEDro Score:** 6 | **Type of program:** PA promoted  **Type of PA**: Overall activity  **PA classification:** Coaching  **Additional strategies:** Behaviour change strategy (mentoring)  **Length of intervention:** 48 weeks  **Unsupervised dose:**  *-Frequency (times/week):* 3  *-Duration (min):* 30  **Delivered by:** Coaches  **Performed with:** Self  **Equipment needed:** No | **Physical activity**  *- # of outcomes:*2  *- % of positive:*100  *- % of positive & significant:* 100  **Falls and fall related injuries**  *- # of outcomes:* 2  *- % of positive:* 100  *- % of positive & significant:* 0  **Wellbeing & QoL**  *- # of outcomes:* 1  *- % of positive:* 100  *- % of positive & significant:* 0 |
| Yates et al (2017)  United Kingdom (High-income) | **N (randomised):** 808  **Inclusion criteria:**  *-Age:* ≥50 years  *- Gender*: Any  *- Physical impairment/limitation:* No  *- Emotional/cognitive status:* No  **PEDro Score:** 7 | **Type of program:** PA promoted  **Type of PA**: Overall activity  **PA classification:** Walking/wheeling  **Additional strategies:** Behaviour change strategies (activity monitor, social activity, incentives)  **Length of intervention:** 156 weeks  **Supervised dose (in person):**  *-Frequency (times/week):* 0.206  *-Duration (min):* 33  **Unsupervised dose:** 156 weeks  *-Frequency (times/week):* 7  *-Duration (min):* 90  **Prescribed by:** Trained educator  **Delivered by:** Class instructors  **Performed with:** Self and others  **Equipment needed:** No | **Physical activity**  *- # of outcomes:* 5  *- % of positive:*100  *- % of positive & significant:* 20  **Intrinsic capacity:** Physical domain  *- # of outcomes:* 13  *- % of positive:* 31  *- % of positive & significant:* 0  **Functional ability:** Emotional & cognitive domain  *- # of outcomes:* 2  *- % of positive:* 100  *- % of positive & significant:* 0  **Wellbeing & QoL**  *- # of outcomes:* 2  *- % of positive:* 50%  *- % of positive & significant:* 0 |

*Multiple papers from the same study are only included once under a study name

**The list of outcomes presented in this table only includes outcomes investigated in these 15 studies

# APPENDIX 9. Data used to create Figures 2-5 presenting the evidence of physical activity services and programs for older adults

## Table A.9.1. Physical activity in all populations and locations by type of activity: impact on different outcome domains (data for Figure 3)

|  |  | **All outcomes** | **Physical activity** | **Falls** | **Intrinsic capacity: physical domain** | **Physical domain:​Functional ability** | **Functional ability: social domain** | **Functional ability: cognitive & emotional domain** | **Well-being &**  **Quality of life** |  |
| --- | --- | --- | --- | --- | --- | --- | --- | --- | --- | --- |
| **All types of PA** | n of intervention groups | 106 | 44 | 38 | 56 | 74 | 11 | 50 | 22 |  |
|  | n of outcomes | 976 | 125 | 82 | 231 | 295 | 22 | 190 | 31 |  |
|  | n(%) of positive | 742  (76%) | 120  (96%) | 71  (87%) | 158  (68%) | 229  (78%) | 14  (64%) | 131  (69%) | 19  (61%) |  |
|  | n(%) of positive & significant | 360  (37%) | 74  (59%) | 30  (37%) | 60  (26%) | 127  (43%) | 6  (27%) | 56  (29%) | 7  (23%) |  |
| **Overall PA** | n of intervention groups | 21 | 21 | 2 | 9 | 13 | 4 | 6 | 6 |  |
|  | n of outcomes | 204 | 70 | 4 | 66 | 27 | 6 | 23 | 8 |  |
|  | n(%) of positive | 171  (84%) | 70  (100%) | 2  (50%) | 53  (80%) | 19  (70%) | 5  (83%) | 16  (70%) | 6  (75%) |  |
|  | n(%) of positive & significant | 76  (37%) | 50  (71%) | 0  (0%) | 13  (20%) | 6  (22%) | 1  (17%) | 4  (17%) | 2  (25%) |  |
| **Structured exercise / Exergames** | n of intervention groups | 61 | 18 | 24 | 35 | 43 | 4 | 30 | 13 |  |
|  | n of outcomes | 539 | 47 | 54 | 124 | 188 | 8 | 99 | 19 |  |
|  | n(%) of positive | 390  (72%) | 43  (91%) | 49  (91%) | 83  (67%) | 145  (77%) | 4  (50%) | 55  (56%) | 11  (58%) |  |
|  | n(%) of positive & significant | 203  (38%) | 22  (47%) | 21  (39%) | 38  (31%) | 89  (47%) | 4  (50%) | 25  (25%) | 4  (21%) |  |
| **Recreation and sports** | n of intervention groups | 24 | 5 | 12 | 12 | 18 | 3 | 14 | 3 |  |
|  | n of outcomes | 233 | 8 | 24 | 41 | 80 | 8 | 68 | 4 |  |
|  | n(%) of positive | 181  (78%) | 7  (88%) | 20  (83%) | 22  (54%) | 65  (81%) | 5  (63%) | 60  (88%) | 2  (50%) |  |
|  | n(%) of positive & significant | 81  (35%) | 2  (25%) | 9  (38%) | 9  (22%) | 32  (40%) | 1  (13%) | 27  (40%) | 1  (25%) |  |
| PA: physical activity, n: number.  This table presents the effect direction and statistical significance for outcome domains across primary studies. All intervention arms from the same study were included as a unique intervention group. Only randomised clinical trials with sample size ≥ 50 participants per group and PEDro score ≥6 were included. | | | | | | | | | | |

## Table A.9.2. Physical activity in all locations by type of structured exercise: impact on different outcome domains (data for Figure 4)

|  |  | **All outcomes** | **Physical activity** | **Falls** | **Intrinsic capacity: physical domain** | **Physical domain:**  **​Functional ability** | **Functional ability: social domain** | **Functional ability: cognitive & emotional domain** | **Well-being &**  **Quality of life** |
| --- | --- | --- | --- | --- | --- | --- | --- | --- | --- |
| **All types of structured exercise/exergame** | n of intervention groups | 60 | 18 | 24 | 35 | 42 | 4 | 30 | 13 |
|  | n of outcomes | 539 | 47 | 54 | 124 | 188 | 8 | 99 | 19 |
|  | n(%) of positive | 390(72%) | 43  (91%) | 49  (91%) | 83  (67%) | 145  (77%) | 4  (50%) | 55  (56%) | 11  (58%) |
|  | n(%) of positive & significant | 203  (38%) | 22  (47%) | 21  (39%) | 38  (31%) | 89  (47%) | 4  (50%) | 25  (25%) | 4  (21%) |
| **Balance, functional, neuromotor** | n of intervention groups | 4 | 0 | 1 | 1 | 4 | 0 | 2 | 2 |
|  | n of outcomes | 29 | 0 | 1 | 3 | 16 | 0 | 7 | 2 |
|  | n(%) of positive | 12  (41%) | 0  (0%) | 1  (0%) | 1  (33%) | 9  (56%) | 0  (0%) | 1  (14%) | 0  (0%) |
|  | n(%) of positive & significant | 3  (10%) | 0  (0%) | 0  (0%) | 0  (0%) | 3  (19%) | 0  (0%) | 0  (0%) | 0  (0%) |
| **Strength, resistance, power** | n of intervention groups | 14 | 2 | 2 | 12 | 5 | 0 | 7 | 1 |
|  | n of outcomes | 99 | 5 | 2 | 43 | 19 | 0 | 29 | 1 |
|  | n(%) of positive | 63  (64%) | 3  (60%) | 2  (100%) | 30  (70%) | 12  (63%) | 0  (0%) | 16  (55%) | 0  (0%) |
|  | n(%) of positive & significant | 25  (25%) | 1  (20%) | 0  (0%) | 15  (35%) | 5  (26%) | 0  (0%) | 4  (14%) | 0  (0%) |
| **Walking** | n of intervention groups | 1 | 0 | 0 | 1 | 1 | 0 | 1 | 1 |
|  | n of outcomes | 5 | 0 | 0 | 1 | 2 | 0 | 1 | 1 |
|  | n(%) of positive | 5  (100%) | 0  (0%) | 0  (0%) | 1  (100%) | 2  (100%) | 0  (0%) | 1  (100%) | 1  (100%) |
|  | n(%) of positive & significant | 5  (100%) | 0  (0%) | 0  (0%) | 1  (100%) | 2  (100%) | 0  (0%) | 1  (100%) | 1  (100%) |
| **Endurance** | n of intervention groups | 2 | 0 | 0 | 1 | 0 | 0 | 2 | 2 |
|  | n of outcomes | 8 | 0 | 0 | 4 | 0 | 0 | 3 | 1 |
|  | n(%) of positive | 7  (88%) | 0  (0%) | 0  (0%) | 3  (75%) | 0  (0%) | 0  (0%) | 3  (100%) | 1  (100%) |
|  | n(%) of positive & significant | 7  (88%) | 0  (0%) | 0  (0%) | 3  (75%) | 0  (0%) | 0  (0%) | 3  (100%) | 1  (100%) |
| **Multicomponent** | n of intervention groups | 39 | 16 | 21 | 20 | 32 | 4 | 18 | 8 |
|  | n of outcomes | 398 | 42 | 51 | 73 | 151 | 8 | 59 | 14 |
|  | n(%) of positive | 303  (76%) | 40  (95%) | 46  (90%) | 48  (66%) | 122  (81%) | 4  (50%) | 34  (58%) | 9  (64%) |
|  | n(%) of positive & significant | 163  (41%) | 21  (50%) | 21  (41%) | 19  (26%) | 7976  (52%) | 4  (50%) | 17  (29%) | 2  (14%) |
| PA: physical activity, n: number.  This table presents the effect direction and statistical significance for outcome domains across primary studies. All intervention arms from the same study were included as a unique intervention group. Only randomised clinical trials with sample size ≥ 50 participants per group and PEDro score ≥6 were included. | | | | | | | | | |

## Table A.9.3. Physical activity in all populations and locations by type of multicomponent exercise: impact on different outcome domains (data for Figure 5)

|  |  | **All outcomes** | **Physical activity** | **Falls** | **Intrinsic capacity: physical domain** | **Physical domain:**  **​Functional ability** | **Functional ability: social domain** | **Functional ability: cognitive & emotional domain** | **Well-being &**  **Quality of life** |
| --- | --- | --- | --- | --- | --- | --- | --- | --- | --- |
| **All types of  multicomponent exercise** | n of intervention groups | 39 | 16 | 21 | 19 | 32 | 4 | 18 | 8 |
|  | n of outcomes | 417 | 46 | 54 | 73 | 155 | 10 | 64 | 15 |
|  | n(%) of positive | 310  (74%) | 44  (96%) | 46  (85%) | 48  (66%) | 121  (78%) | 5  (50%) | 36  (56%) | 10  (67%) |
|  | n(%) of positive & significant | 161  (39%) | 21  (46%) | 21  (39%) | 19  (26%) | 77  (50%) | 4  (40%) | 17  (27%) | 2  (13%) |
| **Presence of balance** | n of intervention groups | 35 | 14 | 20 | 19 | 30 | 3 | 18 | 8 |
|  | n of outcomes | 387 | 38 | 46 | 70 | 152 | 5 | 62 | 14 |
|  | n(%) of positive | 289  (75%) | 37  (97%) | 41  (89%) | 46  (66%) | 120  (79%) | 1  (20%) | 35  (56%) | 9  (64%) |
|  | n(%) of positive & significant | 149  (39%) | 17  (45%) | 19  (41%) | 17  (24%) | 76  (50%) | 1  (20%) | 17  (27%) | 2  (14%) |
| **Presence of strength, resistance, power** | n of intervention groups | 38 | 16 | 21 | 19 | 31 | 4 | 18 | 8 |
|  | n of outcomes | 392 | 43 | 52 | 69 | 144 | 8 | 62 | 14 |
|  | n(%) of positive | 294  (75%) | 41  (95%) | 46  (88%) | 47  (68%) | 112  (78%) | 4  (50%) | 35  (56%) | 9  (64%) |
|  | n(%) of positive & significant | 155  (40%) | 21  (49%) | 21  (40%) | 19  (28%) | 71  (49%) | 4  (50%) | 17  (27%) | 2  (14%) |
| **Presence of walking** | n of intervention groups | 18 | 10 | 10 | 10 | 15 | 2 | 10 | 5 |
|  | n of outcomes | 204 | 25 | 18 | 52 | 64 | 4 | 34 | 7 |
|  | n(%) of positive | 144  (71%) | 25  (100%) | 16  (89%) | 30  (58%) | 47  (73%) | 0  (0%) | 22  (65%) | 4  (57%) |
|  | n(%) of positive & significant | 70  (34%) | 13  (52%) | 6  (33%) | 9  (17%) | 29  (45%) | 0  (0%) | 12  (35%) | 1  (14%) |
| **Presence of endurance** | n of intervention groups | 8 | 2 | 1 | 4 | 6 | 1 | 4 | 1 |
|  | n of outcomes | 54 | 5 | 6 | 7 | 22 | 3 | 10 | 1 |
|  | n(%) of positive | 45  (83%) | 4  (80%) | 6  (100%) | 6  (86%) | 19  (86%) | 3  (100%) | 7  (70%) | 0  (0%) |
|  | n(%) of positive & significant | 38  (70%) | 4  (80%) | 5  (83%) | 5  (71%) | 16  (73%) | 3  (100%) | 5  (50%) | 0  (0%) |
| PA: physical activity, n: number.  This table presents the effect direction and statistical significance for outcome domains across primary studies. All intervention arms from the same study were included as a unique intervention group. Only randomised clinical trials with sample size ≥ 50 participants per group and PEDro score ≥6 were included. | | | | | | | | | |

## Table A.9.4. Physical activity in all locations by type of recreation/sport: impact on different outcome domains (data for Figure 6)

|  |  | **All outcomes** | **Physical activity** | **Falls** | **Intrinsic capacity: physical domain** | **Physical domain:**  **​Functional ability** | **Functional ability: social domain** | **Functional ability: cognitive & emotional domain** | **Well-being &**  **Quality of life** |
| --- | --- | --- | --- | --- | --- | --- | --- | --- | --- |
| **All types of  recreation & sport** | n of intervention groups | 24 | 5 | 11 | 12 | 18 | 3 | 14 | 3 |
|  | n of outcomes | 233 | 8 | 24 | 41 | 80 | 8 | 68 | 4 |
|  | n(%) of positive | 181  (78%) | 7  (88%) | 20  (83%) | 22  (54%) | 65  (81%) | 5  (63%) | 60  (88%) | 2  (50%) |
|  | n(%) of positive & significant | 81  (35%) | 2  (25%) | 9  (38%) | 9  (22%) | 32  (40%) | 1  (13%) | 27  (40%) | 1  (25%) |
| **Tai Chi** | n of intervention groups | 15 | 3 | 10 | 10 | 13 | 3 | 7 | 2 |
|  | n of outcomes | 142 | 4 | 22 | 39 | 39 | 8 | 28 | 2 |
|  | n(%) of positive | 107  (75%) | 4  (100%) | 19  (86%) | 22  (56%) | 33  (85%) | 5  (63%) | 22  (79%) | 2  (100%) |
|  | n(%) of positive & significant | 46  (32%) | 0  (0%) | 8  (36%) | 9  (23%) | 21  (54%) | 1  (13%) | 6  (21%) | 1  (50%) |
| **Yoga/ Pilates** | n of intervention groups | 3 | 0 | 0 | 0 | 1 | 0 | 2 | 0 |
|  | n of outcomes | 7 | 0 | 0 | 0 | 3 | 0 | 4 | 0 |
|  | n(%) of positive | 7  (100%) | 0  (0%) | 0  (0%) | 0  (0%) | 3  (100%) | 0  (0%) | 4  (100%) | 0  (0%) |
|  | n(%) of positive & significant | 4  (57%) | 0  (0%) | 0  (0%) | 0  (0%) | 0  (0%) | 0  (0%) | 4  (100%) | 0%  (0%) |
| **Dance** | n of intervention groups | 5 | 2 | 2 | 1 | 3 | 0 | 4 | 1 |
|  | n of outcomes | 71 | 4 | 2 | 1 | 37 | 0 | 25 | 2 |
|  | n(%) of positive | 57  (80%) | 3  (75%) | 1  (50%) | 0  (0%) | 29  (78%) | 0  (0%) | 24  (96%) | 0  (0%) |
|  | n(%) of positive & significant | 28  (39%) | 2  (50%) | 1  (50%) | 0  (0%) | 11  (30%) | 0  (0%) | 14  (56%) | 0  (0%) |
| **Non-competitive sport** | n of intervention groups | 0 | 0 | 0 | 0 | 0 | 0 | 0 | 0 |
|  | n of outcomes | 0 | 0 | 0 | 0 | 0 | 0 | 0 | 0 |
|  | n(%) of positive | 0  (0%) | 0  (0%) | 0  (0%) | 0  (0%) | 0  (0%) | 0  (0%) | 0  (0%) | 0  (0%) |
|  | n(%) of positive & significant | 0  (0%) | 0  (0%) | 0  (0%) | 0  (0%) | 0  (0%) | 0  (0%) | 0  (0%) | 0  (0%) |
| **Competitive Sport** | n of intervention groups | 1 | 0 | 0 | 1 | 1 | 0 | 1 | 0 |
|  | n of outcomes | 13 | 0 | 0 | 1 | 1 | 0 | 11 | 0 |
|  | n(%) of positive | 10  (77%) | 0  (0%) | 0  (0%) | 0  (0%) | 0  (0%) | 0  (0%) | 10  (91%) | 0  (0%) |
|  | n(%) of positive & significant | 3  (23%) | 0  (0%) | 0  (0%) | 0  (0%) | 0  (0%) | 0  (0%) | 3  (27%) | 0  (0%) |
| PA: physical activity, n: number.  This table presents the effect direction and statistical significance for outcome domains across primary studies. All intervention arms from the same study were included as a unique intervention group. Only randomised clinical trials with sample size ≥ 50 participants per group and PEDro score ≥6 were included. | | | | | | | | | |

# APPENDIX 10. Impact of physical activity types on different outcome domains in adults with physical impairments


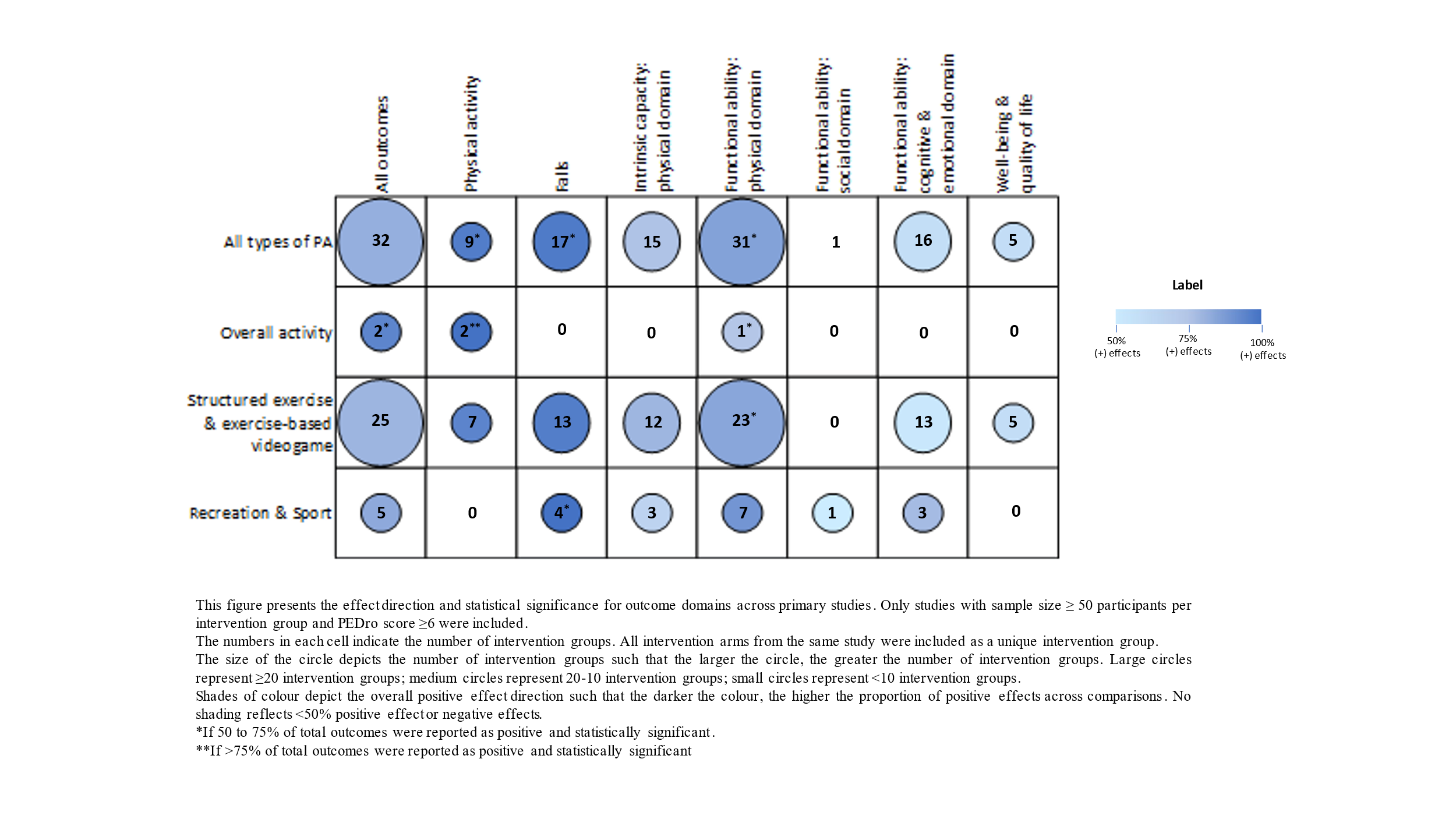


## Figure A.10. Physical activity in adults with physical impairments/limitations by type of activity: impact on different outcome domains

## Table A.10. Physical activity in people with physical impairments/limitations in all locations by type of activity: impact on different outcome domains (data for Figure A.10)

|  |  | **All outcomes** | **Physical activity** | **Falls** | **Intrinsic capacity: physical domain** | **Physical domain:**  **​Functional ability** | **Functional ability: social domain** | **Functional ability: cognitive & emotional domain** | **Well-being &**  **Quality of life** |
| --- | --- | --- | --- | --- | --- | --- | --- | --- | --- |
| **All types of PA** | n of intervention groups | 32 | 9 | 17 | 15 | 31 | 1 | 16 | 5 |
|  | n of outcomes | 349 | 33 | 42 | 42 | 164 | 6 | 57 | 5 |
|  | n(%) of positive | 286  (82%) | 32  (97%) | 41  (98%) | 32  (76%) | 141  (86%) | 3  (50%) | 34  (60%) | 3  (60%) |
|  | n(%) of positive & significant | 154  (44%) | 20  (61%) | 21  (50%) | 19  (45%) | 85  (52%) | 0  (0%) | 8  (14%) | 1  (20%) |
| **Overall PA** | n of intervention groups | 2 | 2 | 0 | 0 | 1 | 0 | 0 | 0 |
|  | n of outcomes | 18 | 14 | 0 | 0 | 4 | 0 | 0 | 0 |
|  | n(%) of positive | 17  (94%) | 14  (100%) | 0 (0%) | 0  (0%) | 3  (75%) | 0  (0%) | 0 (0%) | 0 (0%) |
|  | n(%) of positive & significant | 13  (72%) | 11  (79%) | 0 (0%) | 0  (0%) | 2  (50%) | 0  (0%) | 0 (0%) | 0 (0%) |
| **Structured exercise** | n of intervention groups | 25 | 7 | 13 | 12 | 23 | 0 | 13 | 5 |
|  | n of outcomes | 239 | 19 | 30 | 30 | 112 | 0 | 43 | 5 |
|  | n(%) of positive | 192  (80%) | 18  (95%) | 29  (97%) | 24  80% | 95  (85%) | 0  (0%) | 23  (53%) | 3  (60%) |
|  | n(%) of positive & significant | 104  (44%) | 9  (47%) | 13  (43%) | 14  (47%) | 62  (55%) | 0  (0%) | 5  (12%) | 1  (20%) |
| **Recreation and sports** | n of intervention groups | 5 | 0 | 4 | 3 | 7 | 1 | 3 | 0 |
|  | n of outcomes | 92 | 0 | 12 | 12 | 48 | 6 | 14 | 0 |
|  | n(%) of positive | 77  (84%) | 0  (0%) | 12  (100%) | 8  (67%) | 43  (90%) | 3  (50%) | 11  (79%) | 0  (0%) |
|  | n(%) of positive & significant | 37  (40%) | 0  (0%) | 8  (67%) | 5  (42%) | 21  (44%) | 0  (0%) | 3  (21%) | 0  (0%) |
| PA: physical activity, n: number.  This table presents the effect direction and statistical significance for outcome domains across primary studies. All intervention arms from the same study were included as a unique intervention group. Only randomised clinical trials with sample size ≥ 50 participants per group and PEDro score ≥6 were included. | | | | | | | | | |

# APPENDIX 11. Impact of physical activity types on outcome domains in adults with mild cognitive impairment or low mood


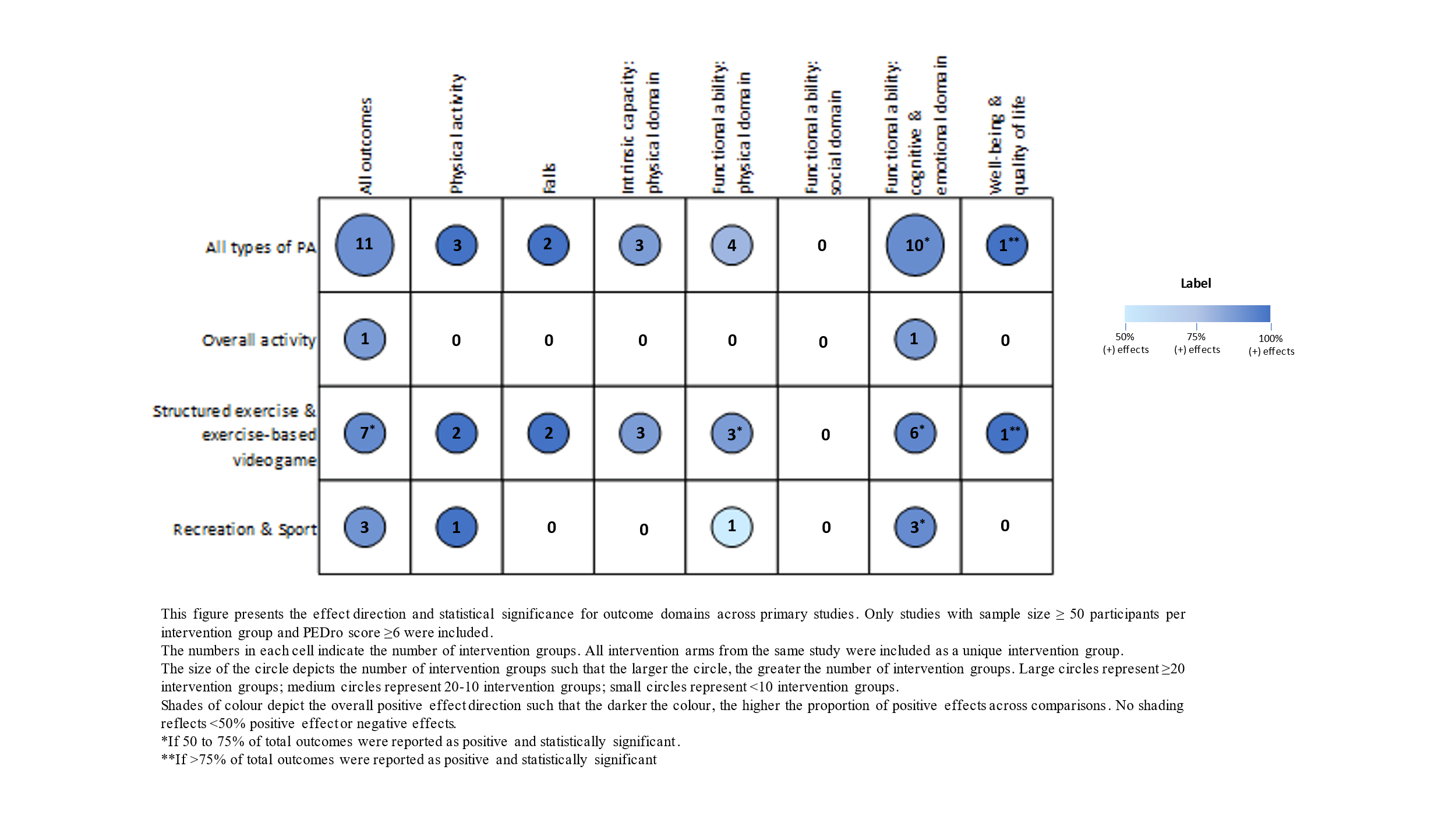


## Figure A.11. Physical activity in adults with mild cognitive impairment or low mood by type of activity: impact on different outcome domains

## Table A.11. Physical activity in people with mild cognitive impairments or mood symptoms in all locations by type of activity: impact on different outcome domains (data for Figure A.11)

|  | | **All outcomes** | **Physical activity** | **Falls** | **Intrinsic capacity: physical domain** | **Physical domain:**  **​Functional ability** | **Functional ability: social domain** | **Functional ability: cognitive & emotional domain** | **Well-being &**  **Quality of life** |
| --- | --- | --- | --- | --- | --- | --- | --- | --- | --- |
| **All types of PA** | n of intervention groups | 11 | 3 | 2 | 3 | 4 | 0 | 10 | 1 |
|  | n of outcomes | 79 | 6 | 3 | 8 | 10 | 0 | 51 | 1 |
|  | n(%) of positive | 72  (91%) | 6  (100%) | 3  (100%) | 7  (88%) | 8  (80%) | 0  (0%) | 47  (92%) | 1  (100%) |
|  | n(%) of positive & significant | 38  (48%) | 2  (33%) | 0  (0%) | 2  (25%) | 4  (40%) | 0  (0%) | 29  (57%) | 1  (100%) |
| **Overall PA** | n of intervention groups | 1 | 0 | 0 | 0 | 0 | 0 | 1 | 0 |
|  | n of outcomes | 8 | 0 | 0 | 0 | 0 | 0 | 8 | 0 |
|  | n(%) of positive | 7  (88%) | 0  (0%) | 0  (0%) | 0  (0%) | 0  (0%) | 0  (0%) | 7  (88%) | 0  (0%) |
|  | n(%) of positive & significant | 3  (38%) | 0  (0%) | 0  (0%) | 0  (0%) | 0  (0%) | 0  (0%) | 3  (38%) | 0  (0%) |
| **Structured exercise** | n of intervention groups | 7 | 2 | 2 | 3 | 3 | 0 | 6 | 1 |
|  | n of outcomes | 41 | 5 | 3 | 8 | 8 | 0 | 16 | 1 |
|  | n(%) of positive | 38  (93%) | 5  (100%) | 3  (100%) | 7  (88%) | 7  (88%) | 0  (0%) | 15  (94%) | 1  (100%) |
|  | n(%) of positive & significant | 21  (51%) | 2  (40%) | 0  (0%) | 2  (25%) | 4  (50%) | 0  (0%) | 12  (75%) | 1  (100%) |
| **Recreation and sports** | n of intervention groups | 3 | 1 | 0 | 0 | 1 | 0 | 3 | 0 |
|  | n of outcomes | 30 | 1 | 0 | 0 | 2 | 0 | 27 | 0 |
|  | n(%) of positive | 27  (90%) | 1  (100%) | 0  (0%) | 0  (0%) | 1  (50%) | 0  (0%) | 25  (93%) | 0  (0%) |
|  | n(%) of positive & significant | 14  (47%) | 0  (0%) | 0  (0%) | 0  (0%) | 0  (0%) | 0  (0%) | 14  (52%) | 0  (0%) |
| PA: physical activity, n: number.  This table presents the effect direction and statistical significance for outcome domains across primary studies. All intervention arms from the same study were included as a unique intervention group. Only randomised clinical trials with sample size ≥ 50 participants per group and PEDro score ≥6 were included. | | | | | | | | | |

# APPENDIX 12. Impact of physical activity on different outcome domains by location


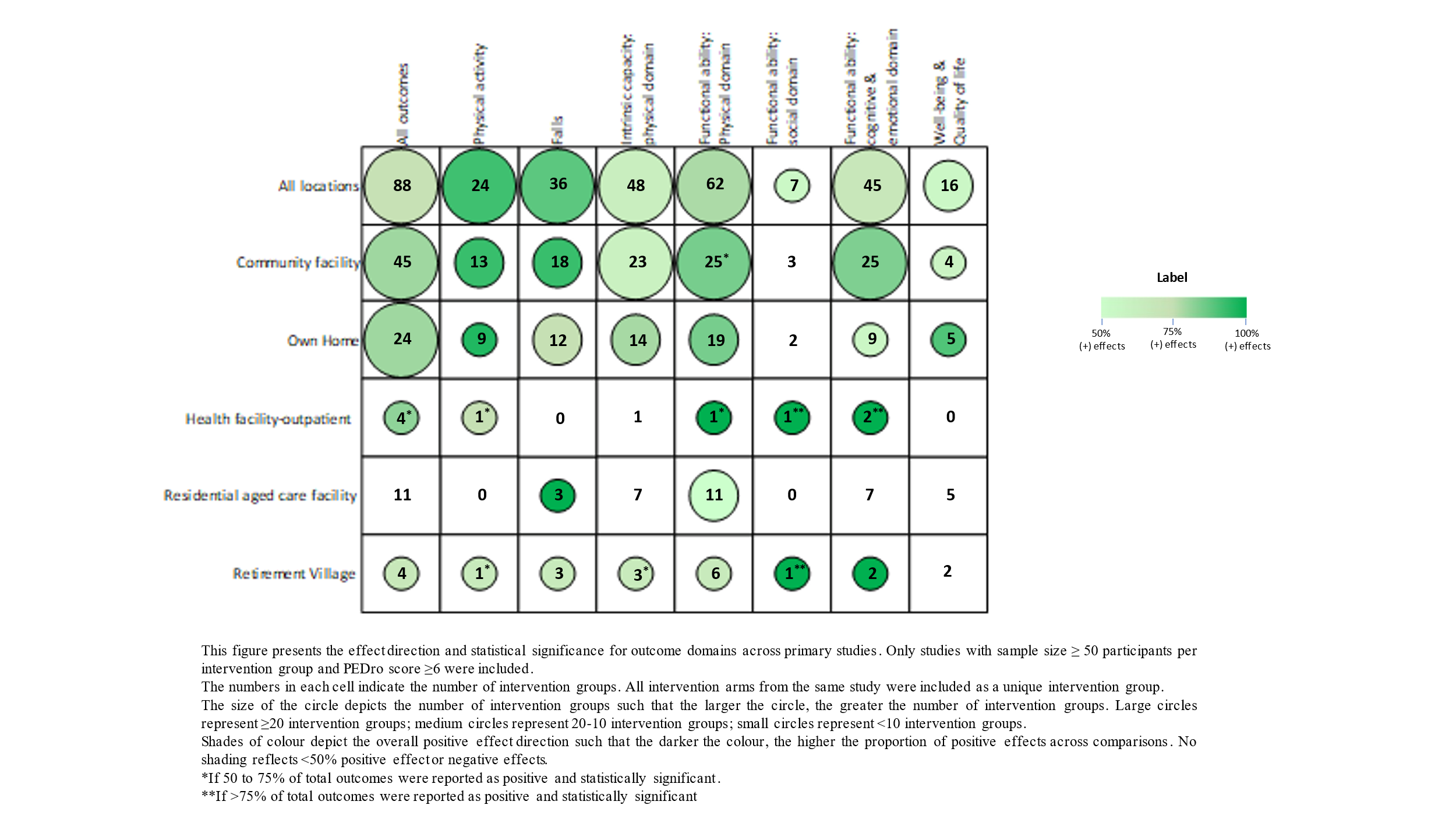


## Figure A.12. Physical activity by location: impact of all types of physical activity on different outcome domains

## Table A.12. Physical activity in all populations by location: impact of all types of physical activity on different outcome domains (data for Figure A.12)

|  |  | **All outcomes** | **Physical activity** | **Falls** | **Intrinsic capacity: physical domain** | **Physical domain:**  **​Functional ability** | **Functional ability: social domain** | **Functional ability: cognitive & emotional domain** | **Well-being &**  **Quality of life** |
| --- | --- | --- | --- | --- | --- | --- | --- | --- | --- |
| **All locations** | n of intervention groups | 88 | 24 | 36 | 48 | 62 | 7 | 45 | 16 |
|  | n of outcomes | 796 | 63 | 78 | 169 | 272 | 16 | 175 | 23 |
|  | n(%) of positive | 593  (74%) | 58  (92%) | 69  (88%) | 109  (64%) | 213  (78%) | 9  (56%) | 122  (70%) | 13  (57%) |
|  | n(%) of positive &  significant | 299  (38%) | 30  (48%) | 30  (38%) | 51  (30%) | 123  (45%) | 5  (31%) | 55  (31%) | 5  (22%) |
| **Community** | n of intervention groups | 45 | 13 | 18 | 23 | 25 | 3 | 25 | 4 |
|  | n of outcomes | 391 | 30 | 45 | 90 | 107 | 9 | 105 | 5 |
|  | n(%) of positive | 312  (80%) | 28  (93%) | 42  (93%) | 56  (62%) | 91  (85%) | 4  (44%) | 88  (84%) | 3  (60%) |
|  | n(%) of positive & significant | 164  (42%) | 13  (43%) | 19  (42%) | 28  (31%) | 57  (53%) | 0  (0%) | 45  (43%) | 2  (40%) |
| **Own home** | n of intervention groups | 24 | 9 | 12 | 14 | 19 | 2 | 9 | 5 |
|  | n of outcomes | 230 | 26 | 20 | 52 | 88 | 3 | 31 | 10 |
|  | n(%) of positive | 184  (80%) | 25  (96%) | 15  (75%) | 41  (79%) | 75  (85%) | 1  (33%) | 18  (58%) | 9  (90%) |
|  | n(%) of positive & significant | 86  (37%) | 12  (46%) | 8  (40%) | 17  (33%) | 41  (47%) | 1  (33%) | 5  (16%) | 2  (20%) |
| **Outpatient health facility** | n of intervention groups | 4 | 1 | 0 | 1 | 1 | 1 | 2 | 0 |
|  | n of outcomes | 22 | 4 | 0 | 4 | 9 | 3 | 2 | 0 |
|  | n(%) of positive | 18  (82%) | 3  (75%) | 0  (0%) | 1  (25%) | 9  (100%) | 3  (100%) | 2  (100%) | 0  (0%) |
|  | n(%) of positive & significant | 14  (64%) | 3  (75%) | 0  (0%) | 0  (0%) | 6  (67%) | 3  (100%) | 2  (100%) | 0  (0%) |
| **Residential aged care facility** | n of intervention groups | 11 | 0 | 3 | 7 | 11 | 0 | 7 | 5 |
|  | n of outcomes | 108 | 0 | 10 | 17 | 44 | 0 | 32 | 5 |
|  | n(%) of positive | 48  (44%) | 0  (0%) | 10  (100% | 7  (41%) | 22  (50%) | 0  (0%) | 9  (28%) | 0  (0%) |
|  | n(%) of positive & significant | 14  (13%) | 0  (0%) | 2  (20%) | 2  (12%) | 9  (20%) | 0  (0%) | 1  (3%) | 0  (0%) |
| **Retirement village** | n of intervention groups | 4 | 1 | 3 | 3 | 6 | 1 | 2 | 2 |
|  | n of outcomes | 45 | 3 | 3 | 6 | 24 | 1 | 5 | 3 |
|  | n(%) of positive | 31  (69%) | 2  (67%) | 2  (67%) | 4  (67%) | 16  (67%) | 1  (100%) | 5  (100%) | 1  (33%) |
|  | n(%) of positive & significant | 21  (47%) | 2  (67%) | 1  (33%) | 4  (67%) | 10  (42%) | 1  (100%) | 2  (40%) | 1  (33%) |
| PA: physical activity, n: number.  This table presents the effect direction and statistical significance for outcome domains across primary studies. All intervention arms from the same study were included as a unique intervention group. Only randomised clinical trials with sample size ≥ 50 participants per group and PEDro score ≥6 were included. | | | | | | | | | |

# APPENDIX 13. Impact of structured exercise in different locations on different outcome domains


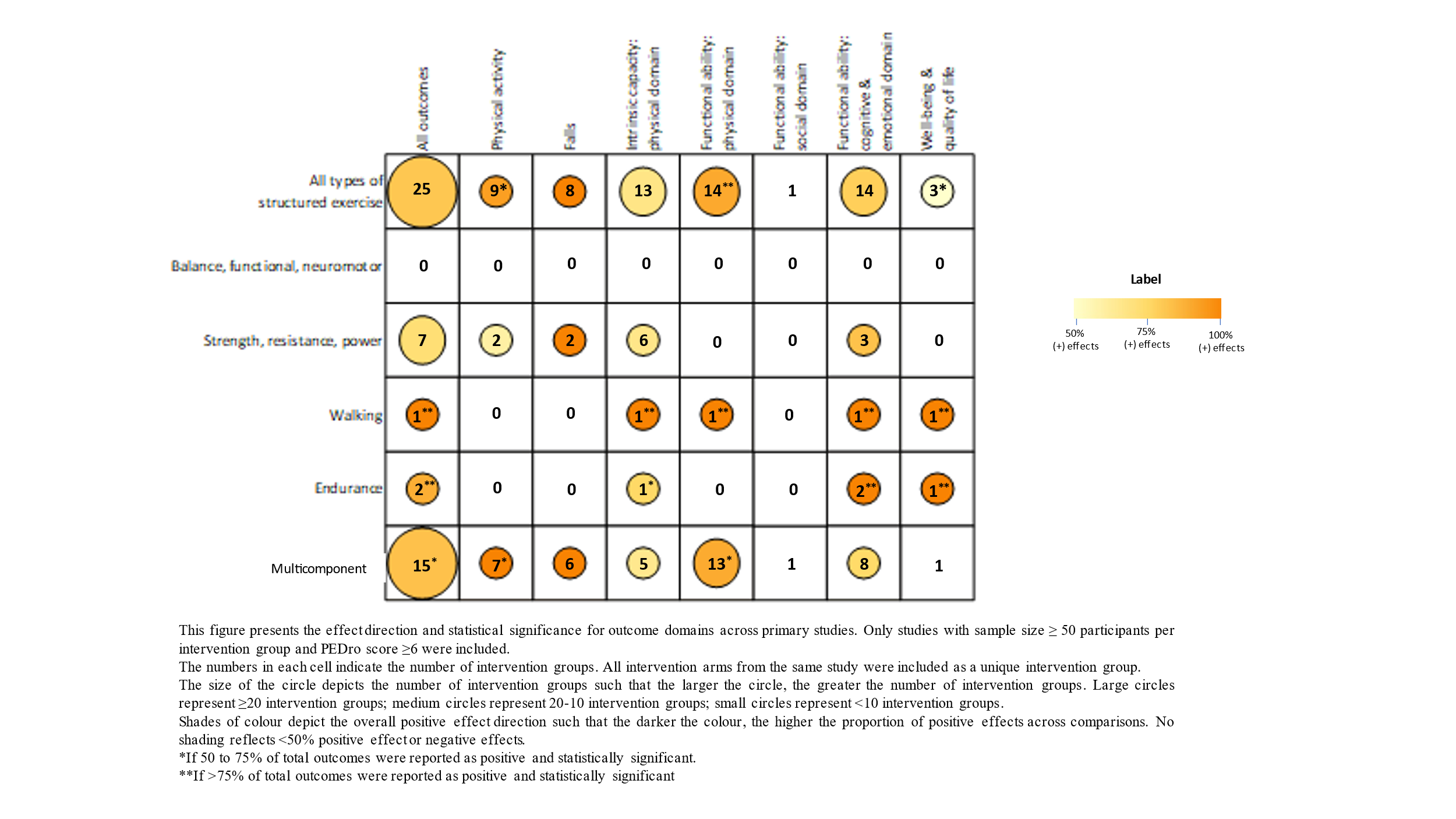


## Figure A.13.1. Physical activity in community facilities by type of structured exercise: impact on different outcome domains

## Table A.13.1. Physical activity in community facilities by type of structured exercise: impact on different outcome domains (data for Figure A.13.1)

|  |  | **All outcomes** | **Physical activity** | **Falls** | **Intrinsic capacity: physical domain** | **Physical domain:**  **​Functional ability** | **Functional ability: social domain** | **Functional ability: cognitive & emotional domain** | **Well-being &**  **Quality of life** |
| --- | --- | --- | --- | --- | --- | --- | --- | --- | --- |
| **All types of**  **structured exercise** | n of intervention groups | 25 | 9 | 8 | 13 | 14 | 1 | 14 | 3 |
|  | n of outcomes | 205 | 25 | 23 | 55 | 46 | 2 | 50 | 4 |
|  | n(%) of positive | 165  (80%) | 23  (92%) | 23  (100%) | 37  (67%) | 41  (89%) | 0  (0%) | 39  (78%) | 2  (50%) |
|  | n(%) of positive & significant | 101  (49%) | 13  (52%) | 10  (43%) | 20  (36%) | 35  (76%) | 0  (0%) | 21  (42%) | 2  (50%) |
| **Balance, functional, neuromotor** | n of intervention groups | 0 | 0 | 0 | 0 | 0 | 0 | 0 | 0 |
|  | n of outcomes | 0 | 0 | 0 | 0 | 0 | 0 | 0 | 0 |
|  | n(%) of positive | 0  (0%) | 0  (0%) | 0  (0%) | 0  (0%) | 0  (0%) | 0  (0%) | 0  (0%) | 0  (0%) |
|  | n(%) of positive & significant | 0  (0%) | 0  (0%) | 0  (0%) | 0  (0%) | 0  (0%) | 0  (0%) | 0  (0%) | 0  (0%) |
| **Strength, resistance, power** | n of intervention groups | 7 | 2 | 2 | 6 | 0 | 0 | 3 | 0 |
|  | n of outcomes | 42 | 5 | 2 | 24 | 0 | 0 | 11 | 0 |
|  | n(%) of positive | 30  (71%) | 3  (60%) | 2  (100%) | 16  (67%) | 0  (0%) | 0  (0%) | 9  (82%) | 0  (0%) |
|  | n(%) of positive & significant | 11  (26%) | 1  (20%) | 0  (0%) | 7  (29%) | 0  (0%) | 0  (0%) | 3  (27%) | 0  (0%) |
| **Walking** | n of intervention groups | 1 | 0 | 0 | 1 | 1 | 0 | 1 | 1 |
|  | n of outcomes | 5 | 0 | 0 | 1 | 2 | 0 | 1 | 1 |
|  | n(%) of positive | 5  (100%) | 0  (0%) | 0  (0%) | 1  (100%) | 2  (100%) | 0  (0%) | 1 (100%) | 1  (100%) |
|  | n(%) of positive & significant | 5  (100%) | 0  (0%) | 0  (0%) | 1  (100%) | 2  (100%) | 0  (0%) | 1  (100%) | 1  (100%) |
| **Endurance** | n of intervention groups | 2 | 0 | 0 | 1 | 0 | 0 | 2 | 1 |
|  | n of outcomes | 8 | 0 | 0 | 4 | 0 | 0 | 3 | 1 |
|  | n(%) of positive | 7  (88%) | 0  (0%) | 0  (0%) | 3  (75%) | 0  (0%) | 0  (0%) | 3  (100%) | 1  (100%) |
|  | n(%) of positive & significant | 7  (88%) | 0  (0%) | 0  (0%) | 3  (75%) | 0  (0%) | 0  (0%) | 3  (100%) | 1  (100%) |
| **Multicomponent** | n of intervention groups | 15 | 7 | 6 | 5 | 13 | 1 | 8 | 1 |
|  | n of outcomes | 150 | 20 | 21 | 26 | 44 | 2 | 35 | 2 |
|  | n(%) of positive | 123  (82%) | 20  (100%) | 21  (100%) | 17  (65%) | 39  (89%) | 0  (0%) | 26  (74%) | 0  (0%) |
|  | n(%) of positive & significant | 78 (52%) | 12 (60%) | 10  (48%) | 9  (35%) | 33  (75%) | 0  (0%) | 14  (40%) | 0  (0%) |
| PA: physical activity, n: number.  This table presents the effect direction and statistical significance for outcome domains across primary studies. All intervention arms from the same study were included as a unique intervention group. Only randomised clinical trials with sample size ≥ 50 participants per group and PEDro score ≥6 were included. | | | | | | | | | |


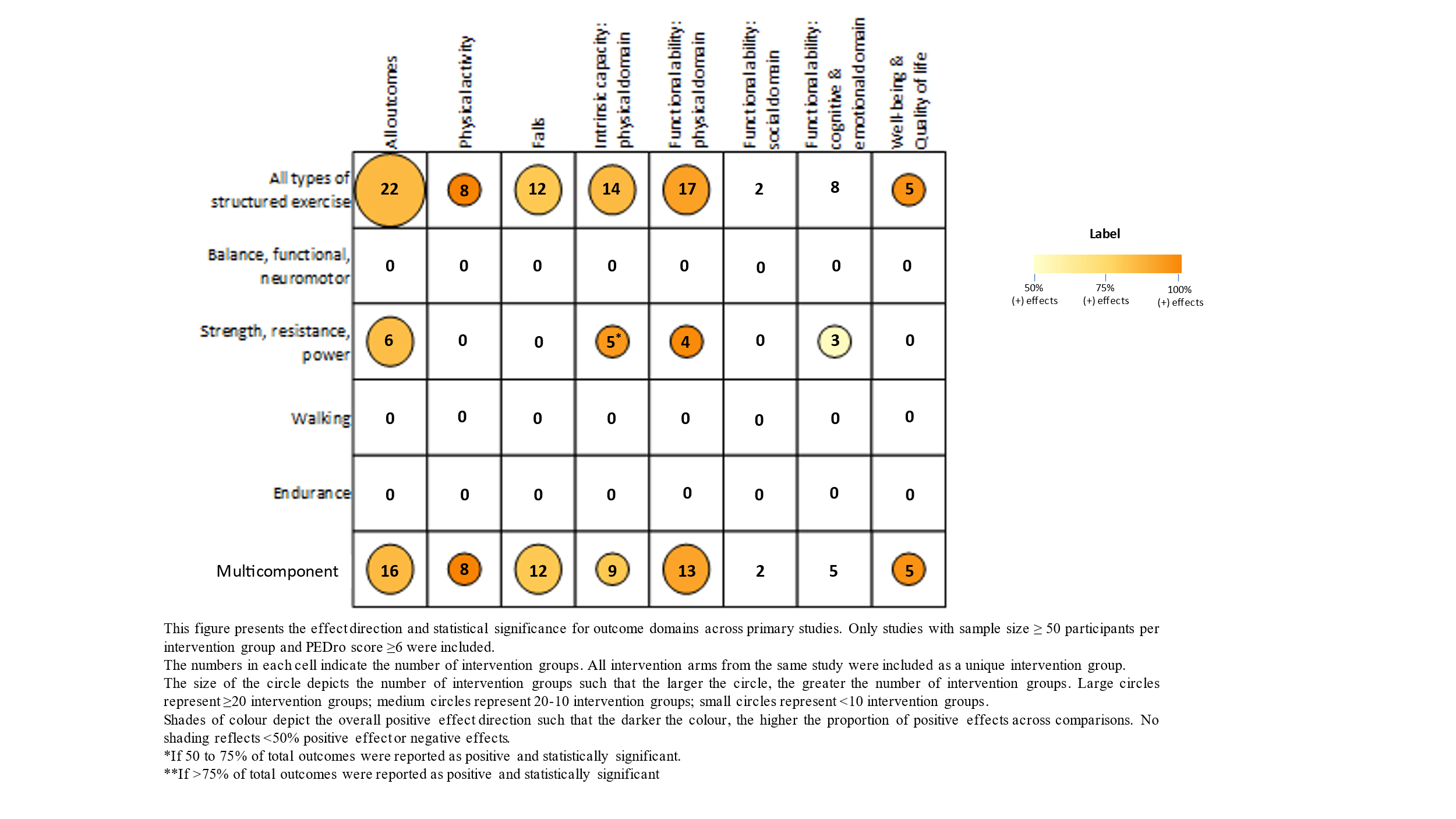


## Figure A.13.2. Physical activity in the home by type of structured exercise: impact on different outcome domains

## Table A.13.2. Physical activity in the home by type of structured exercise: impact on different outcome domains (data for Figure A.13.2)

|  |  | **All outcomes** | **Physical activity** | **Falls** | **Intrinsic capacity: physical domain** | **Physical domain:**  **​Functional ability** | **Functional ability: social domain** | **Functional ability: cognitive & emotional domain** | **Well-being &**  **Quality of life** |
| --- | --- | --- | --- | --- | --- | --- | --- | --- | --- |
| **All types of  structured exercise** | n of intervention groups | 22 | 8 | 12 | 14 | 17 | 2 | 8 | 5 |
|  | n of outcomes | 210 | 18 | 20 | 52 | 84 | 3 | 23 | 10 |
|  | n(%) of positive | 166  (79%) | 17  (94%) | 15  (75%) | 41  (79%) | 72  (86%) | 1  (33%) | 11  (48%) | 9  (90%) |
|  | n(%) of positive & significant | 75  (36%) | 6  (33%) | 8  (40%) | 17  (33%) | 39  (46%) | 1  (33%) | 2  (9%) | 2  (20%) |
| **Balance, functional, neuromotor** | n of intervention groups | 0 | 0 | 0 | 0 | 0 | 0 | 0 | 0 |
|  | n of outcomes | 0 | 0 | 0 | 0 | 0 | 0 | 0 | 0 |
|  | n(%) of positive | 0  (0%) | 0  (0%) | 0  (0%) | 0  (0%) | 0  (0%) | 0  (0%) | 0  (0%) | 0  (0%) |
|  | n(%) of positive & significant | 0  (0%) | 0  (0%) | 0  (0%) | 0  (0%) | 0  (0%) | 0  (0%) | 0  (0%) | 0  (0%) |
| **Strength, resistance, power** | n of intervention groups | 6 | 0 | 0 | 5 | 4 | 0 | 3 | 0 |
|  | n of outcomes | 40 | 0 | 0 | 16 | 12 | 0 | 12 | 0 |
|  | n(%) of positive | 31  (78%) | 0  (0%) | 0  (0%) | 14  (88%) | 11  (92%) | 0  (0%) | 6  50% | 0  (0%) |
|  | n(%) of positive & significant | 14  (35%) | 0  (0%) | 0  (0%) | 8  (50%) | 5  (42%) | 0  (0%) | 1  8% | 0  (0%) |
| **Walking** | n of intervention groups | 0 | 0 | 0 | 0 | 0 | 0 | 0 | 0 |
|  | n of outcomes | 0 | 0 | 0 | 0 | 0 | 0 | 0 | 0 |
|  | n(%) of positive | 0  (0%) | 0  (0%) | 0  (0%) | 0  (0%) | 0  (0%) | 0  (0%) | 0  (0%) | 0  (0%) |
|  | n(%) of positive & significant | 0  (0%) | 0  (0%) | 0  (0%) | 0  (0%) | 0  (0%) | 0  (0%) | 0  (0%) | 0  (0%) |
| **Endurance** | n of intervention groups | 0 | 0 | 0 | 0 | 0 | 0 | 0 | 0 |
|  | n of outcomes | 0 | 0 | 0 | 0 | 0 | 0 | 0 | 0 |
|  | n(%) of positive | 0  (0%) | 0  (0%) | 0  (0%) | 0  (0%) | 0  (0%) | 0  (0%) | 0  (0%) | 0  (0%) |
|  | n(%) of positive & significant | 0  (0%) | 0  (0%) | 0  (0%) | 0  (0%) | 0  (0%) | 0  (0%) | 0  (0%) | 0  (0%) |
| **Multicomponent** | n of intervention groups | 16 | 8 | 12 | 9 | 13 | 2 | 5 | 5 |
|  | n of outcomes | 170 | 18 | 20 | 36 | 72 | 3 | 11 | 10 |
|  | n(%) of positive | 135  (79%) | 17  (94%) | 15  (75%) | 27  (75%) | 61  (85%) | 1  (33%) | 5  (45%) | 9  (90%) |
|  | n(%) of positive & significant | 61  (36%) | 6  (33%) | 8  (40%) | 9  (25%) | 34  (47%) | 1  (33%) | 1  (9%) | 2  (20%) |
| PA: physical activity, n: number.  This table presents the effect direction and statistical significance for outcome domains across primary studies. All intervention arms from the same study were included as a unique intervention group. Only randomised clinical trials with sample size ≥ 50 participants per group and PEDro score ≥6 were included. | | | | | | | | | |


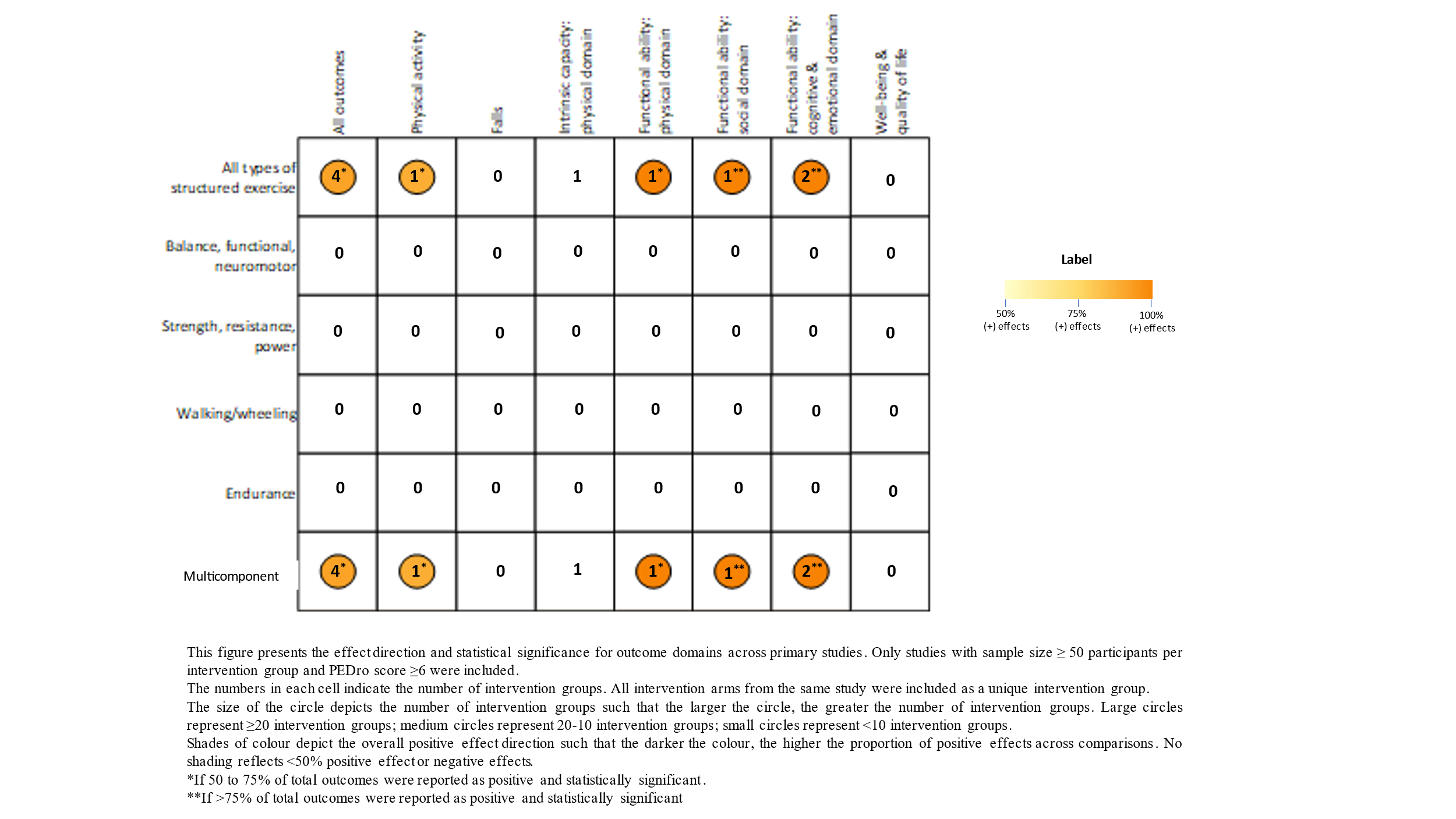


## Figure A.13.3. Physical activity in outpatient health facilities by type of structured exercise: impact on different outcome domains

## Table A.13.3. Physical activity in outpatient health facilities by type of structured exercise: impact on different outcome domains (data for Figure A.13.3)

|  |  | **All outcomes** | **Physical activity** | **Falls** | **Intrinsic capacity: physical domain** | **Physical domain:**  **​Functional ability** | **Functional ability: social domain** | **Functional ability: cognitive & emotional domain** | **Well-being &**  **Quality of life** |
| --- | --- | --- | --- | --- | --- | --- | --- | --- | --- |
| **All types of structured exercise** | n of intervention groups | 4 | 1 | 0 | 1 | 1 | 1 | 2 | 0 |
|  | n of outcomes | 22 | 4 | 0 | 4 | 9 | 3 | 2 | 0 |
|  | n(%) of positive | 18  (82%) | 3  (75%) | 0  (0%) | 1  (25%) | 9  (100%) | 3  (100%) | 2  (100%) | 0  (0%) |
|  | n(%) of positive & significant | 14  (64%) | 3  (75%) | 0  (0%) | 0  (0%) | 6  (67%) | 3  (100%) | 2  (100%) | 0  (0%) |
| **Balance, functional, neuromotor** | n of intervention groups | 0 | 0 | 0 | 0 | 0 | 0 | 0 | 0 |
|  | n of outcomes | 0 | 0 | 0 | 0 | 0 | 0 | 0 | 0 |
|  | n(%) of positive | 0  (0%) | 0  (0%) | 0  (0%) | 0  (0%) | 0  (0%) | 0  (0%) | 0  (0%) | 0  (0%) |
|  | n(%) of positive & significant | 0  (0%) | 0  (0%) | 0  (0%) | 0  (0%) | 0  (0%) | 0  (0%) | 0  (0%) | 0  (0%) |
| **Strength, resistance, power** | n of intervention groups | 0 | 0 | 0 | 0 | 0 | 0 | 0 | 0 |
|  | n of outcomes | 0 | 0 | 0 | 0 | 0 | 0 | 0 | 0 |
|  | n(%) of positive | 0  (0%) | 0  (0%) | 0  (0%) | 0  (0%) | 0  (0%) | 0  (0%) | 0  (0%) | 0  (0%) |
|  | n(%) of positive & significant | 0  (0%) | 0  (0%) | 0  (0%) | 0  (0%) | 0  (0%) | 0  (0%) | 0  (0%) | 0  (0%) |
| **Walking** | n of intervention groups | 0 | 0 | 0 | 0 | 0 | 0 | 0 | 0 |
|  | n of outcomes | 0 | 0 | 0 | 0 | 0 | 0 | 0 | 0 |
|  | n(%) of positive | 0  (0%) | 0  (0%) | 0  (0%) | 0  (0%) | 0  (0%) | 0  (0%) | 0  (0%) | 0  (0%) |
|  | n(%) of positive & significant | 0  (0%) | 0  (0%) | 0  (0%) | 0  (0%) | 0  (0%) | 0  (0%) | 0  (0%) | 0  (0%) |
| **Endurance** | n of intervention groups | 0 | 0 | 0 | 0 | 0 | 0 | 0 | 0 |
|  | n of outcomes | 0 | 0 | 0 | 0 | 0 | 0 | 0 | 0 |
|  | n(%) of positive | 0  (0%) | 0  (0%) | 0  (0%) | 0  (0%) | 0  (0%) | 0  (0%) | 0  (0%) | 0  (0%) |
|  | n(%) of positive & significant | 0  (0%) | 0  (0%) | 0  (0%) | 0  (0%) | 0  (0%) | 0  (0%) | 0  (0%) | 0  (0%) |
| **Multicomponent** | n of intervention groups | 4 | 1 | 0 | 1 | 1 | 1 | 2 | 0 |
|  | n of outcomes | 22 | 4 | 0 | 4 | 9 | 3 | 2 | 0 |
|  | n(%) of positive | 18  (82%) | 3  (75%) | 0  (0%) | 1  (25%) | 9  (100%) | 3  (100%) | 2  (100%) | 0  (0%) |
|  | n(%) of positive & significant | 14  (64%) | 3  (75%) | 0  (0%) | 0  (0%) | 6  (67%) | 3  (100%) | 2  (100%) | 0  (0%) |
| PA: physical activity, n: number.  This table presents the effect direction and statistical significance for outcome domains across primary studies. All intervention arms from the same study were included as a unique intervention group. Only randomised clinical trials with sample size ≥ 50 participants per group and PEDro score ≥6 were included. | | | | | | | | | |


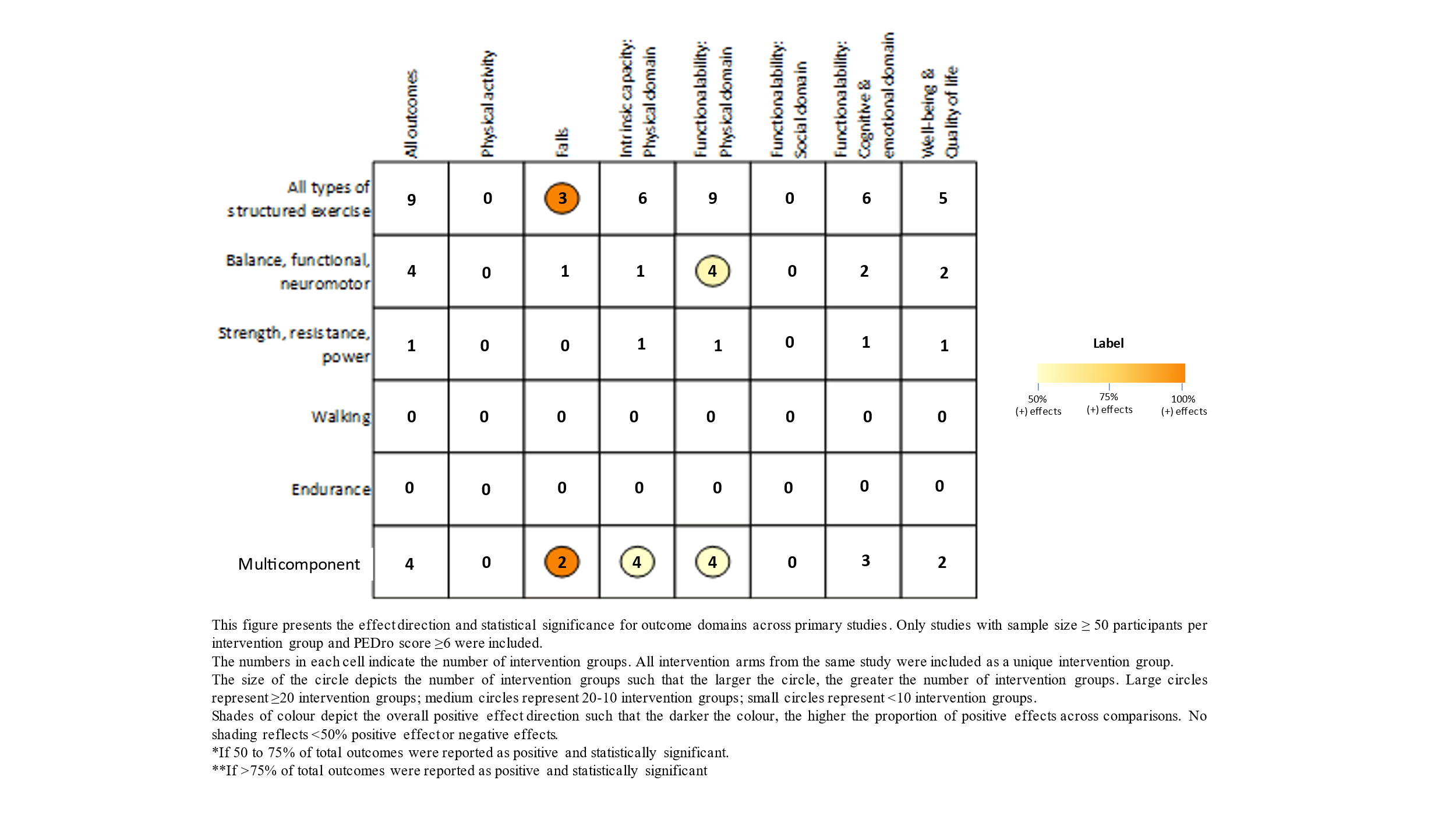


## Figure A.13.4. Physical activity in residential aged care facilities by type of structured exercise: impact on different outcome domains

## Table A.13.4. Physical activity in residential aged care facilities by type of structured exercise: impact on outcome domains (data for Figure A.13.4)

|  |  | **All outcomes** | **Physical activity** | **Falls** | **Intrinsic capacity: physical domain** | **Physical domain:**  **​Functional ability** | **Functional ability: social domain** | **Functional ability: cognitive & emotional domain** | **Well-being &**  **Quality of life** |
| --- | --- | --- | --- | --- | --- | --- | --- | --- | --- |
| **All types of  structured exercise** | n of intervention groups | 9 | 0 | 3 | 6 | 9 | 0 | 6 | 5 |
|  | n of outcomes | 92 | 0 | 10 | 12 | 41 | 0 | 24 | 5 |
|  | n(%) of positive | 36  (39%) | 0  (0%) | 10  (100%) | 4  (33%) | 19  (46%) | 0  (0%) | 3  (13%) | 0  (0%) |
|  | n(%) of positive & significant | 10  (11%) | 0  (0%) | 2  (20%) | 1  (8%) | 7  (17%) | 0  (0%) | 0  (0%) | 0  (0%) |
| **Balance, functional, neuromotor** | n of intervention groups | 4 | 0 | 1 | 1 | 4 | 0 | 2 | 2 |
|  | n of outcomes | 29 | 0 | 1 | 3 | 16 | 0 | 7 | 2 |
|  | n(%) of positive | 12  (41%) | 0  (0%) | 1  (0%) | 1  (33%) | 9  (56%) | 0  (0%) | 1  (14%) | 0  (0%) |
|  | n(%) of positive & significant | 3  (10%) | 0  (0%) | 0  (0%) | 0  (0%) | 3  (19%) | 0  (0%) | 0  (0%) | 0  (0%) |
| **Strength, resistance, power** | n of intervention groups | 1 | 0 | 0 | 1 | 1 | 0 | 1 | 1 |
|  | n of outcomes | 17 | 0 | 0 | 3 | 7 | 0 | 6 | 1 |
|  | n(%) of positive | 2  (12%) | 0  (0%) | 0  (0%) | 0  (0%) | 1  (14%) | 0  (0%) | 1  (17%) | 0  (0%) |
|  | n(%) of positive & significant | 0  (0%) | 0  (0%) | 0  (0%) | 0  (0%) | 0  (0%) | 0  (0%) | 0  (0%) | 0  (0%) |
| **Walking** | n of intervention groups | 0 | 0 | 0 | 0 | 0 | 0 | 0 | 0 |
|  | n of outcomes | 0 | 0 | 0 | 0 | 0 | 0 | 0 | 0 |
|  | n(%) of positive | 0  (0%) | 0  (0%) | 0  (0%) | 0  (0%) | 0  (0%) | 0  (0%) | 0  (0%) | 0  (0%) |
|  | n(%) of positive & significant | 0  (0%) | 0  (0%) | 0  (0%) | 0  (0%) | 0  (0%) | 0  (0%) | 0  (0%) | 0  (0%) |
| **Endurance** | n of intervention groups | 0 | 0 | 0 | 0 | 0 | 0 | 0 | 0 |
|  | n of outcomes | 0 | 0 | 0 | 0 | 0 | 0 | 0 | 0 |
|  | n(%) of positive | 0  (0) | 0  (0%) | 0  (0%) | 0  (0%) | 00 | 0  (0%) | 0  (0%) | 0  (0%) |
|  | n(%) of positive & significant | 00%) | 0  (0%) | 0  (0%) | 0  (0%) | 00 | 0  (0%) | 0  (0%) | 0  (0%) |
| **Multicomponent** | n of intervention groups | 4 | 0 | 2 | 4 | 4 | 0 | 3 | 2 |
|  | n of outcomes | 46 | 0 | 9 | 6 | 18 | 0 | 11 | 2 |
|  | n(%) of positive | 22  (48%) | 0  (0%) | 9  (100%) | 3  (50%) | 9  (50%) | 0  (0%) | 1  (9%) | 0  (0%) |
|  | n(%) of positive & significant | 7  (15%) | 0  (0%) | 2  (22%) | 1  (17%) | 4  (22%) | 0  (0%) | 0  (0%) | 0  (0%) |
| PA: physical activity, n: number.  This table presents the effect direction and statistical significance for outcome domains across primary studies. All intervention arms from the same study were included as a unique intervention group. Only randomised clinical trials with sample size ≥ 50 participants per group and PEDro score ≥6 were included. | | | | | | | | | |


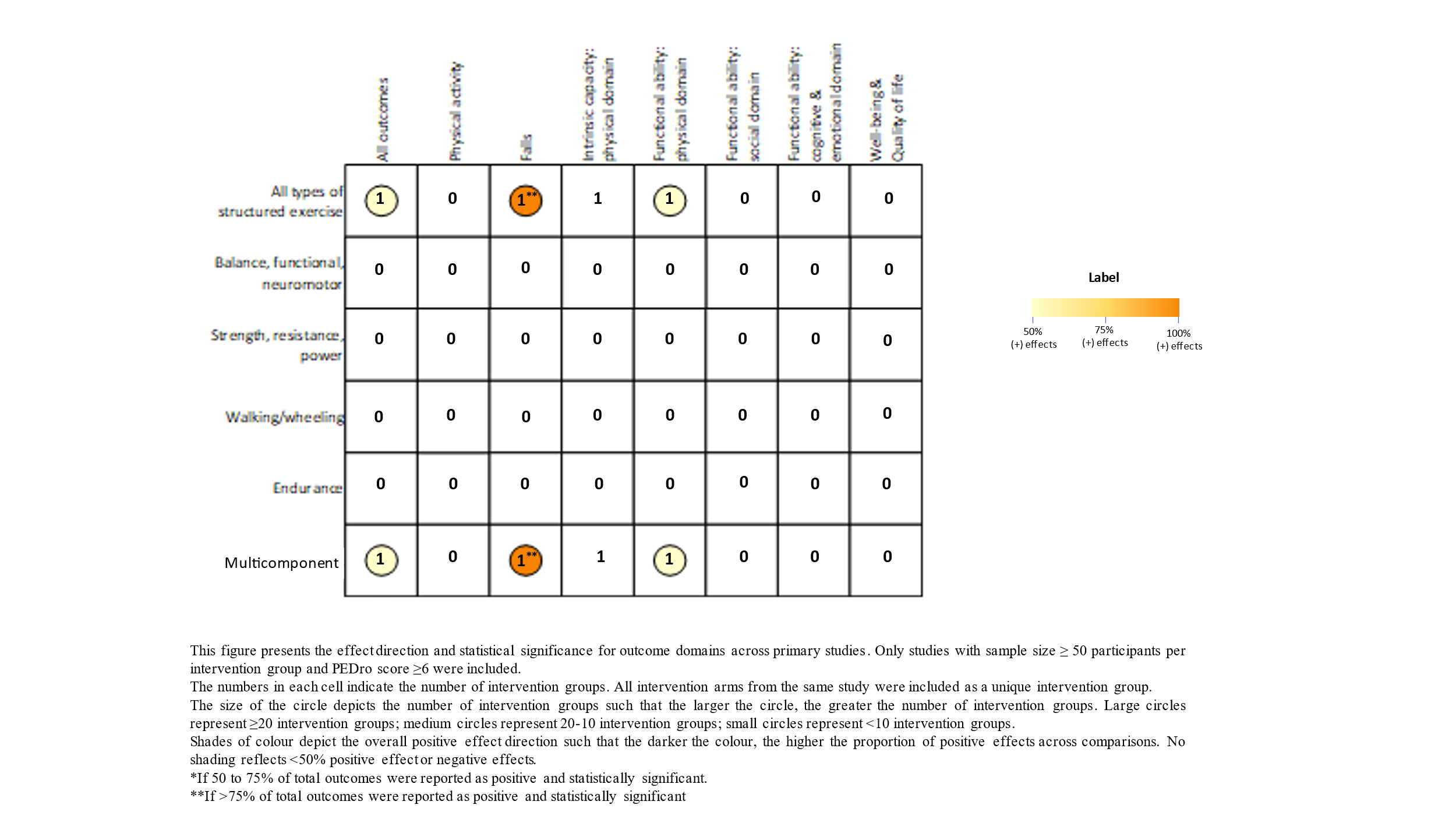


## Figure A.13.5. Physical activity in retirement villages by type of structured exercise: impact on different outcome domains

## Table A.13.5. Physical activity in retirement villages by type of structured exercise: impact on different outcome domains (data for Figure A.13.5)

|  |  | **All outcomes** | **Physical activity** | **Falls** | **Intrinsic capacity: physical domain** | **Physical domain:**  **​Functional ability** | **Functional ability: social domain** | **Functional ability: cognitive & emotional domain** | **Well-being &**  **Quality of life** |
| --- | --- | --- | --- | --- | --- | --- | --- | --- | --- |
| **All types of  structured exercise** | n of intervention groups | 1 | 0 | 1 | 1 | 1 | 0 | 0 | 0 |
|  | n of outcomes | 10 | 0 | 1 | 1 | 8 | 0 | 0 | 0 |
|  | n(%) of positive | 5  (50%) | 0  (0%) | 1  (100%) | 0  (0%) | 4  (50%) | 0  (0%) | 0  (0%) | 0  (0%) |
|  | n(%) of positive & significant | 3  (30%) | 0  (0%) | 1  (100%) | 0  (0%) | 2  (25%) | 0  (0%) | 0  (0%) | 0  (0%) |
| **Balance, functional, neuromotor** | n of intervention groups | 0 | 0 | 0 | 0 | 0 | 0 | 0 | 0 |
|  | n of outcomes | 0 | 0 | 0 | 0 | 0 | 0 | 0 | 0 |
|  | n(%) of positive | 0  (0%) | 0  (0%) | 0  (0%) | 0  (0%) | 0  (0%) | 0  (0%) | 0  (0%) | 0  (0%) |
|  | n(%) of positive & significant | 0  (0%) | 0  (0%) | 0  (0%) | 0  (0%) | 0  (0%) | 0  (0%) | 0  (0%) | 0  (0%) |
| **Strength, resistance, power** | n of intervention groups | 0 | 0 | 0 | 0 | 0 | 0 | 0 | 0 |
|  | n of outcomes | 0 | 0 | 0 | 0 | 0 | 0 | 0 | 0 |
|  | n(%) of positive | 0  (0%) | 0  (0%) | 0  (0%) | 0  (0%) | 0  (0%) | 0  (0%) | 0  (0%) | 0  (0%) |
|  | n(%) of positive & significant | 0  (0%) | 0  (0%) | 0  (0%) | 0  (0%) | 0  (0%) | 0  (0%) | 0  (0%) | 0  (0%) |
| **Walking** | n of intervention groups | 0 | 0 | 0 | 0 | 0 | 0 | 0 | 0 |
|  | n of outcomes | 0 | 0 | 0 | 0 | 0 | 0 | 0 | 0 |
|  | n(%) of positive | 0  (0%) | 0  (0%) | 0  (0%) | 0  (0%) | 0  (0%) | 0  (0%) | 0  (0%) | 0  (0%) |
|  | n(%) of positive & significant | 0  (0%) | 0  (0%) | 0  (0%) | 0  (0%) | 0  (0%) | 0  (0%) | 0  (0%) | 0  (0%) |
| **Endurance** | n of intervention groups | 0 | 0 | 0 | 0 | 0 | 0 | 0 | 0 |
|  | n of outcomes | 0 | 0 | 0 | 0 | 0 | 0 | 0 | 0 |
|  | n(%) of positive | 0  (0%) | 0  (0%) | 0  (0%) | 0  (0%) | 0  (0%) | 0  (0%) | 0  (0%) | 0  (0%) |
|  | n(%) of positive & significant | 0  (0%) | 0  (0%) | 0  (0%) | 0  (0%) | 0  (0%) | 0  (0%) | 0  (0%) | 0  (0%) |
| **Multicomponent** | n of intervention groups | 1 | 0 | 1 | 1 | 1 | 0 | 0 | 0 |
|  | n of outcomes | 10 | 0 | 1 | 1 | 8 | 0 | 0 | 0 |
|  | n(%) of positive | 5  (50%) | 0  (0%) | 1  (100%) | 0  (0%) | 4  (50%) | 0  (0%) | 0  (0%) | 0  (0%) |
|  | n(%) of positive & significant | 3  (30%) | 0  (0%) | 1  (100%) | 0  (0%) | 2  (25%) | 0  (0%) | 0  (0%) | 0  (0%) |
| PA: physical activity, n: number.  This table presents the effect direction and statistical significance for outcome domains across primary studies. All intervention arms from the same study were included as a unique intervention group. Only randomised clinical trials with sample size ≥ 50 participants per group and PEDro score ≥6 were included. | | | | | | | | | |

# APPENDIX 14. Impact of recreation/sport in different locations on different outcome domains


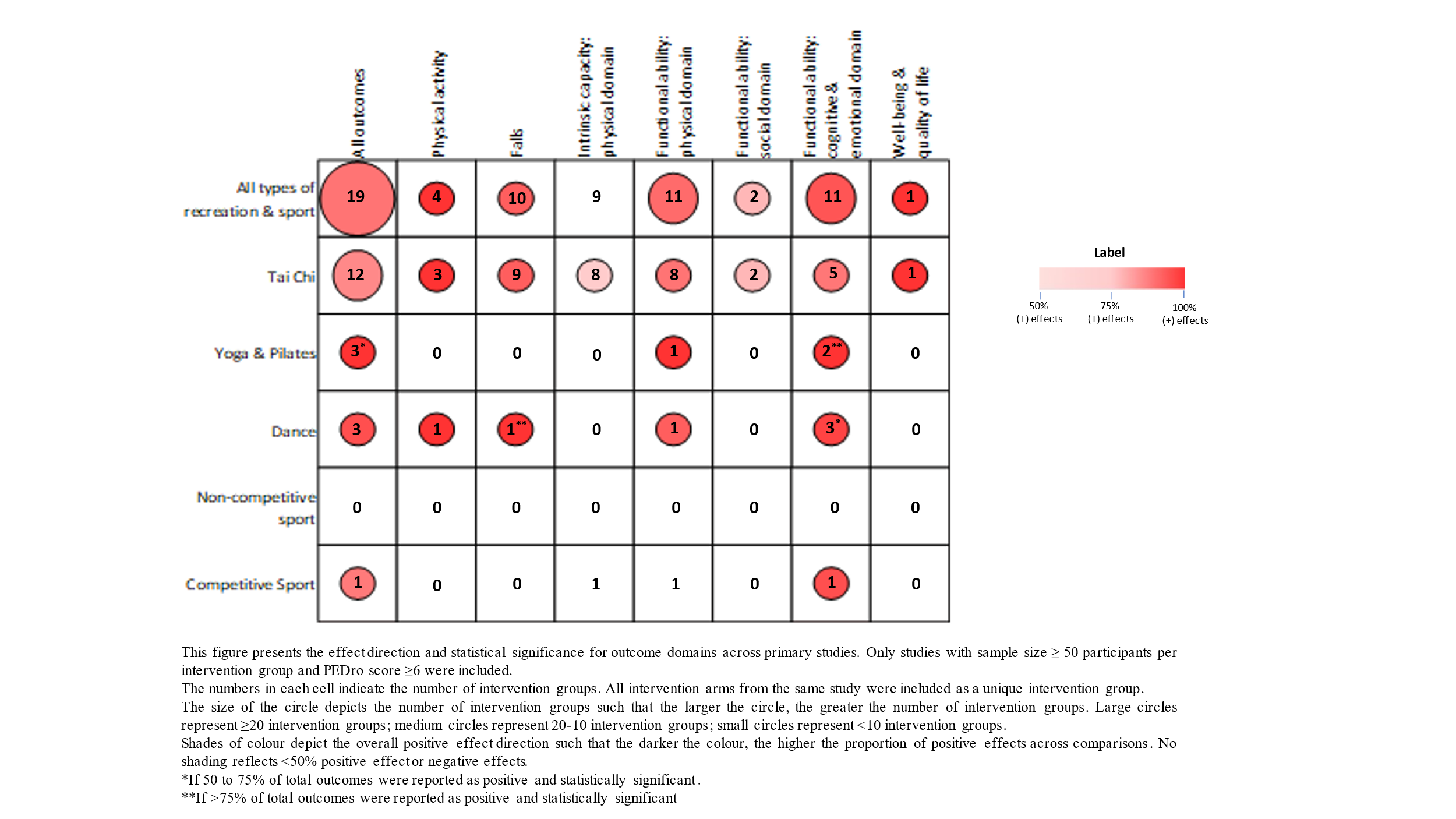


## Figure A.14.1. Physical activity in community facilities by type of recreation/sport: impact on different outcome domains

## Table A.14.1. Physical activity in community facilities by type of recreation/sport: impact on different outcome domains (data for Figure A.14.1)

|  |  | **All outcomes** | **Physical activity** | **Falls** | **Intrinsic capacity: physical domain** | **Physical domain:**  **​Functional ability** | **Functional ability: social domain** | **Functional ability: cognitive & emotional domain** | **Well-being &**  **Quality of life** |  |
| --- | --- | --- | --- | --- | --- | --- | --- | --- | --- | --- |
| **All types of**  **recreation & sport** | n of intervention groups | 19 | 4 | 10 | 9 | 11 | 2 | 11 | 1 |  |
|  | n of outcomes | 182 | 5 | 22 | 31 | 61 | 7 | 55 | 1 |  |
|  | n(%) of positive | 143  (79%) | 5  (100%) | 19  (86%) | 15  (48%) | 50  (82%) | 4  (57%) | 49  (89%) | 1  (100%) |  |
|  | n(%) of positive & significant | 59  (32%) | 0  (0%) | 9  (41%) | 4  (13%) | 22  (36%) | 0  (0%) | 24  (44%) | 0  (0%) |  |
| **Tai Chi** | n of intervention groups | 12 | 3 | 9 | 8 | 8 | 2 | 5 | 1 |  |
|  | n of outcomes | 109 | 4 | 21 | 30 | 28 | 7 | 18 | 1 |  |
|  | n(%) of positive | 78  (72%) | 4  (100%) | 18  (86%) | 15  (50%) | 22  (79%) | 4  (57%) | 14  (78%) | 1  (100%) |  |
|  | n(%) of positive & significant | 28  (26%) | 0  (0%) | 8  (38%) | 4  (13%) | 13  (46%) | 0  (0%) | 3  (17%) | 0  (0%) |  |
| **Yoga/ Pilates** | n of intervention groups | 3 | 0 | 0 | 0 | 1 | 0 | 2 | 0 |  |
|  | n of outcomes | 7 | 0 | 0 | 0 | 3 | 0 | 4 | 0 |  |
|  | n(%) of positive | 7  (100%) | 0  (0%) | 0  (0%) | 0  (0%) | 3  (100%) | 0  (0%) | 4  (100%) | 0  (0%) |  |
|  | n(%) of positive & significant | 4  (57%) | 0  (0%) | 0  (0%) | 0  (0%) | 0  (0%) | 0  (0%) | 4  (100%) | 0  (0%) |  |
| **Dance** | n of intervention groups | 3 | 1 | 1 | 0 | 1 | 0 | 3 | 0 |  |
|  | n of outcomes | 53 | 1 | 1 | 0 | 29 | 0 | 22 | 0 |  |
|  | n(%) of positive | 48  (91%) | 1  (100%) | 1  (100%) | 0  (0%) | 25  (86%) | 0  (0%) | 21  (95%) | 0  (0%) |  |
|  | n(%) of positive & significant | 24  (45%) | 0  (0%) | 1  (100%) | 0  (0%) | 9  (31%) | 0  (0%) | 14  (64%) | 0  (0%) |  |
| **Non-competitive sport** | n of intervention groups | 0 | 0 | 0 | 0 | 0 | 0 | 0 | 0 |  |
|  | n of outcomes | 0 | 0 | 0 | 0 | 0 | 0 | 0 | 0 |  |
|  | n(%) of positive | 0  (0%) | 0  (0%) | 0  (0%) | 0  (0%) | 0  (0%) | 0  (0%) | 0  (0%) | 0  (0%) |  |
|  | n(%) of positive & significant | 0  (0%) | 0  (0%) | 0  (0%) | 0  (0%) | 0  (0%) | 0  (0%) | 0  (0%) | 0  (0%) |  |
| **Competitive Sport** | n of intervention groups | 1 | 0 | 0 | 1 | 1 | 0 | 1 | 0 |  |
|  | n of outcomes | 13 | 0 | 0 | 1 | 1 | 0 | 11 | 0 |  |
|  | n(%) of positive | 10  (77%) | 0  (0%) | 0  (0%) | 0  (0%) | 0  (0%) | 0  (0%) | 10  (91%) | 0  (0%) |  |
|  | n(%) of positive & significant | 3  (23%) | 0  (0%) | 0  (0%) | 0  (0%) | 0  (0%) | 0  (0%) | 3  (27%) | 0  (0%) |  |
| PA: physical activity, n: number.  This table presents the effect direction and statistical significance for outcome domains across primary studies. All intervention arms from the same study were included as a unique intervention group. Only randomised clinical trials with sample size ≥ 50 participants per group and PEDro score ≥6 were included. | | | | | | | | | |  |


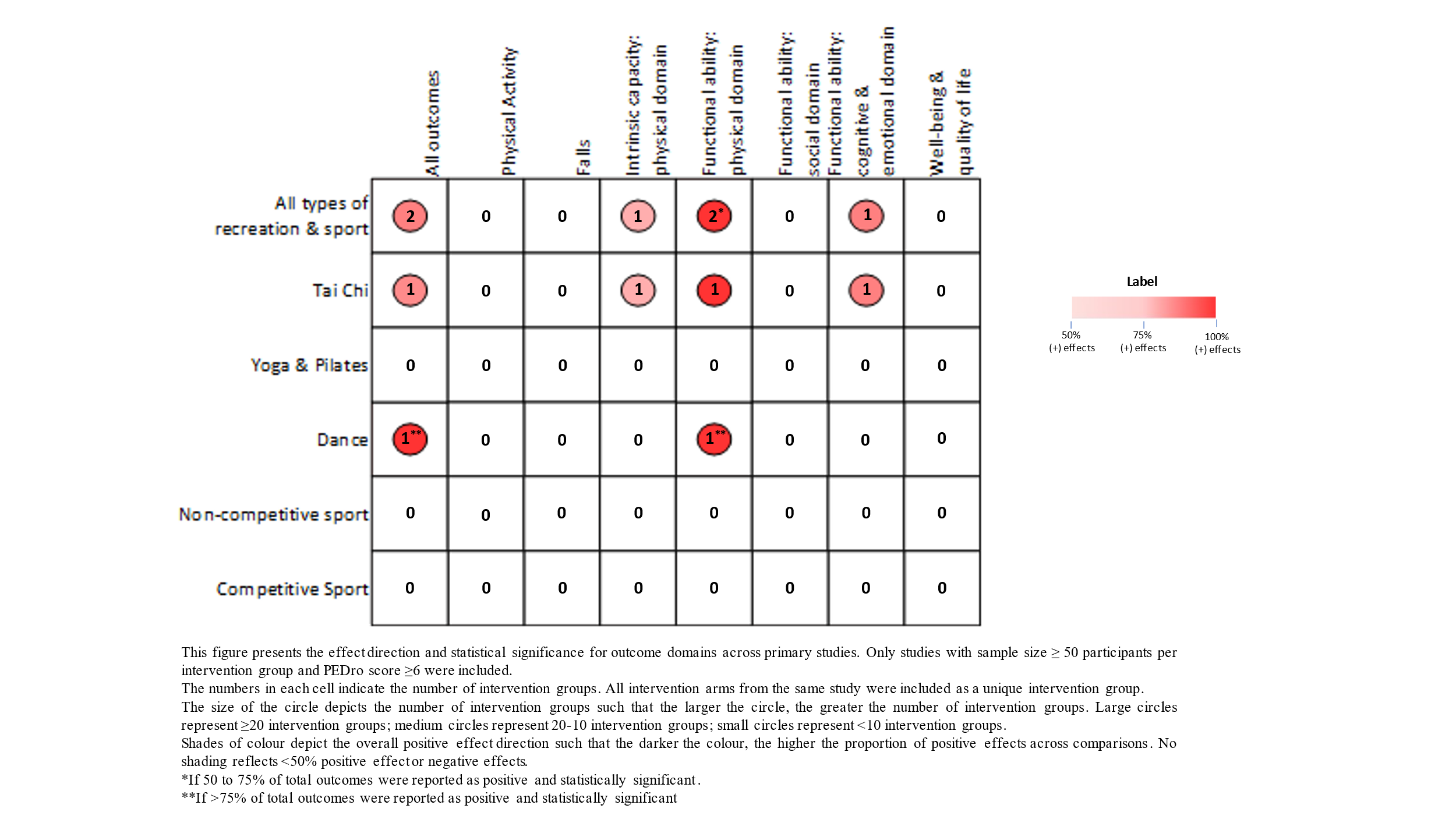


## Figure A.14.2. Physical activity in residential aged care facilities by type of recreation/sport: impact on different outcome domains

## Table A.14.2. Physical activity in residential aged care facilities by type of recreation/sport: impact on different outcome domains (data for Figure A.14.2)

|  |  | **All outcomes** | **Physical activity** | **Falls** | **Intrinsic capacity: physical domain** | **Physical domain:**  **​Functional ability** | **Functional ability: social domain** | **Functional ability: cognitive & emotional domain** | **Well-being &**  **Quality of life** |
| --- | --- | --- | --- | --- | --- | --- | --- | --- | --- |
| **All types of  recreation & sport** | n of intervention groups | 2 | 0 | 0 | 1 | 2 | 0 | 1 | 0 |
|  | n of outcomes | 16 | 0 | 0 | 5 | 3 | 0 | 8 | 0 |
|  | n(%) of positive | 12  (75%) | 0  (0%) | 0  (0%) | 3  (60%) | 3  (100%) | 0  (0%) | 6  (75%) | 0  (0%) |
|  | n(%) of positive & significant | 4  (25%) | 0  (0%) | 0  (0%) | 1  (20%) | 2  (67%) | 0  (0%) | 1  (13%) | 0  (0%) |
| **Tai Chi** | n of intervention groups | 1 | 0 | 0 | 1 | 1 | 0 | 1 | 0 |
|  | n of outcomes | 14 | 0 | 0 | 5 | 1 | 0 | 8 | 0 |
|  | n(%) of positive | 10  (71%) | 0  (0%) | 0  (0%) | 3  (60%) | 1  (100%) | 0  (0%) | 6  (75%) | 0  (0%) |
|  | n(%) of positive & significant | 2  (14%) | 0  (0%) | 0  (0%) | 1  (20%) | 0  (0%) | 0  (0%) | 1  (13%) | 0  (0%) |
| **Yoga/Pilates** | n of intervention groups | 0 | 0 | 0 | 0 | 0 | 0 | 0 | 0 |
|  | n of outcomes | 0 | 0 | 0 | 0 | 0 | 0 | 0 | 0 |
|  | n(%) of positive | 0  (0%) | 0  (0%) | 0  (0%) | 0  (0%) | 0  (0%) | 0  (0%) | 0  (0%) | 0  (0%) |
|  | n(%) of positive & significant | 0  (0%) | 0  (0%) | 0  (0%) | 0  (0%) | 0  (0%) | 0  (0%) | 0  (0%) | 0  (0%) |
| **Dance** | n of intervention groups | 1 | 0 | 0 | 0 | 1 | 0 | 0 | 0 |
|  | n of outcomes | 2 | 0 | 0 | 0 | 2 | 0 | 0 | 0 |
|  | n(%) of positive | 2  (100%) | 0  (0%) | 0  (0%) | 0  (0%) | 2  (100%) | 0  (0%) | 0  (0%) | 0  (0%) |
|  | n(%) of positive & significant | 2  (100%) | 0  (0%) | 0  (0%) | 0  (0%) | 2  (100%) | 0  (0%) | 0  (0%) | 0  (0%) |
| **Non-competitive sport** | n of intervention groups | 0 | 0 | 0 | 0 | 0 | 0 | 0 | 0 |
|  | n of outcomes | 0 | 0 | 0 | 0 | 0 | 0 | 0 | 0 |
|  | n(%) of positive | 0  (0%) | 0  (0%) | 0  (0%) | 0  (0%) | 0  (0%) | 0  (0%) | 0  (0%) | 0  (0%) |
|  | n(%) of positive & significant | 0  (0%) | 0  (0%) | 0  (0%) | 0  (0%) | 0  (0%) | 0  (0%) | 0  (0%) | 0  (0%) |
| **Competitive Sport** | n of intervention groups | 0 | 0 | 0 | 0 | 0 | 0 | 0 | 0 |
|  | n of outcomes | 0 | 0 | 0 | 0 | 0 | 0 | 0 | 0 |
|  | n(%) of positive | 0  (0%) | 0  (0%) | 0  (0%) | 0  (0%) | 0  (0%) | 0  (0%) | 0  (0%) | 0  (0%) |
|  | n(%) of positive & significant | 0  (0%) | 0  (0%) | 0  (0%) | 0  (0%) | 0  (0%) | 0  (0%) | 0  (0%) | 0  (0%) |
| PA: physical activity, n: number.  This table presents the effect direction and statistical significance for outcome domains across primary studies. All intervention arms from the same study were included as a unique intervention group. Only randomised clinical trials with sample size ≥ 50 participants per group and PEDro score ≥6 were included. | | | | | | | | | |


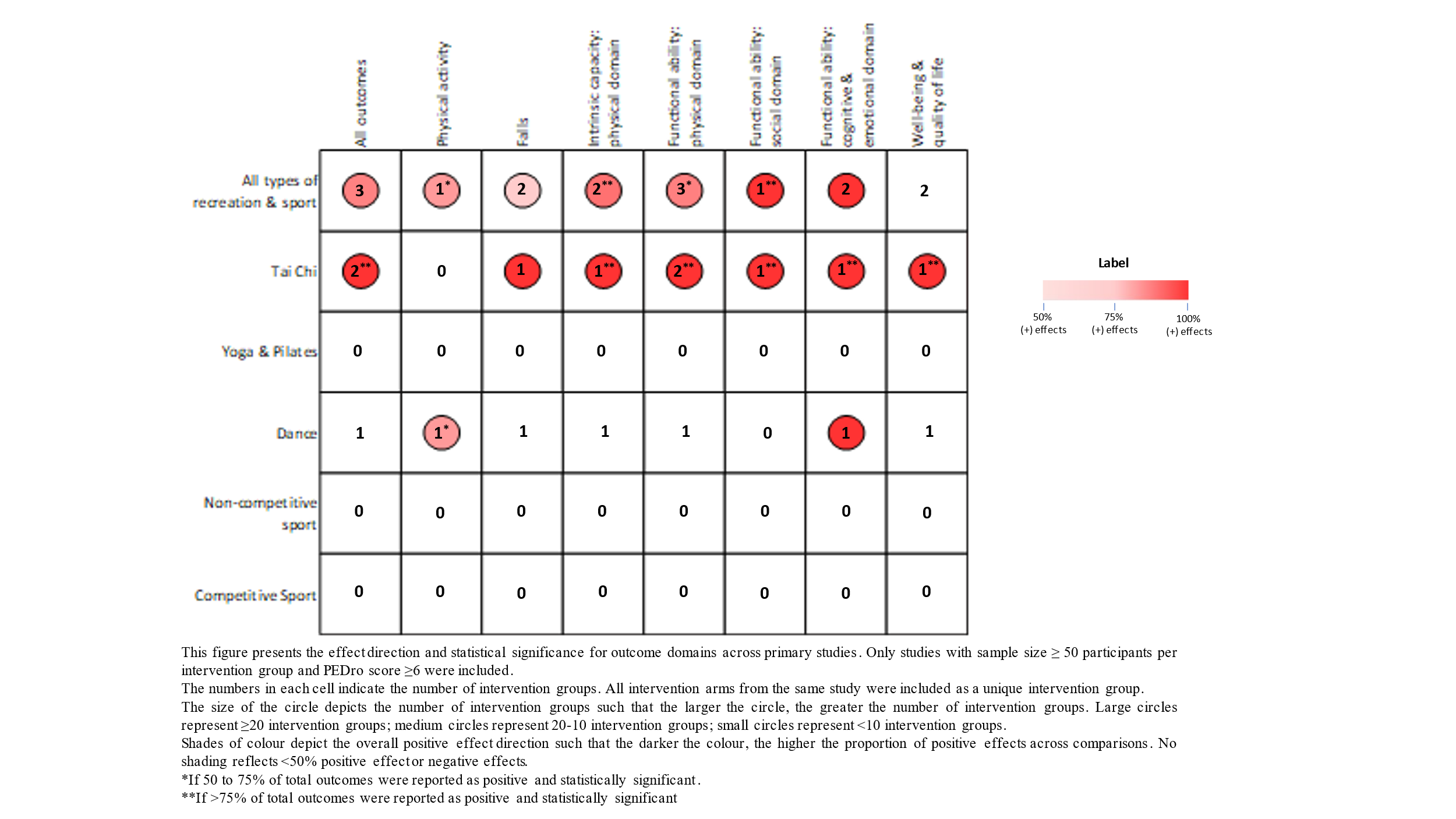


## Figure A.14.3. Physical activity in retirement villages by type of recreation/sport: impact on different outcome domains

## Table A.14.3. Physical activity in retirement villages by type of recreation/sport: impact on different outcome (data for Figure A.14.3)

|  |  | **All outcomes** | **Physical activity** | **Falls** | **Intrinsic capacity: physical domain** | **Physical domain:**  **​Functional ability** | **Functional ability: social domain** | **Functional ability: cognitive & emotional domain** | **Well-being &**  **Quality of life** |
| --- | --- | --- | --- | --- | --- | --- | --- | --- | --- |
| **All types of  recreation & sport** | n of intervention groups | 3 | 1 | 2 | 2 | 3 | 1 | 2 | 2 |
|  | n of outcomes | 35 | 3 | 2 | 5 | 16 | 1 | 5 | 3 |
|  | n(%) of positive | 26  (74%) | 2  (67%) | 1  (50%) | 4  (80%) | 12  (75%) | 1  (100%) | 5  (100%) | 1  (33%) |
|  | n(%) of positive & significant | 18  (51%) | 2  (67%) | 0  (0%) | 4  (80%) | 8  (50%) | 1  (100%) | 2  (40%) | 1  (33%) |
| **Tai Chi** | n of intervention groups | 2 | 0 | 1 | 1 | 2 | 1 | 1 | 1 |
|  | n of outcomes | 19 | 0 | 1 | 4 | 10 | 1 | 2 | 1 |
|  | n(%) of positive | 19  (100%) | 0  (0%) | 1  (100%) | 4  (100%) | 10  (100%) | 1  (100%) | 2  (100%) | 1  (100%) |
|  | n(%) of positive & significant | 16  (84%) | 0  (0%) | 0  (0%) | 4  (100%) | 8  (80%) | 1  (100%) | 2  (100%) | 1  (100%) |
| **Yoga** | n of intervention groups | 0 | 0 | 0 | 0 | 0 | 0 | 0 | 0 |
|  | n of outcomes | 0 | 0 | 0 | 0 | 0 | 0 | 0 | 0 |
|  | n(%) of positive | 0  (0%) | 0  (0%) | 0  (0%) | 0  (0%) | 0  (0%) | 0  (0%) | 0  (0%) | 0  (0%) |
|  | n(%) of positive & significant | 0  (0%) | 0  (0%) | 0  (0%) | 0  (0%) | 0  (0%) | 0  (0%) | 0  (0%) | 0  (0%) |
| **Dance** | n of intervention groups | 1 | 1 | 1 | 1 | 1 | 0 | 1 | 1 |
|  | n of outcomes | 16 | 3 | 1 | 1 | 6 | 0 | 3 | 2 |
|  | n(%) of positive | 7  (44%) | 2  (67%) | 0  (0%) | 0  (0%) | 2  (33%) | 0  (0%) | 3  (100%) | 0  (0%) |
|  | n(%) of positive & significant | 2  (13%) | 2  (67%) | 0  (0%) | 0  (0%) | 0  (0%) | 0  (0%) | 0  (0%) | 0  (0%) |
| **Non-competitive sport** | n of intervention groups | 0 | 0 | 0 | 0 | 0 | 0 | 0 | 0 |
|  | n of outcomes | 0 | 0 | 0 | 0 | 0 | 0 | 0 | 0 |
|  | n(%) of positive | 0  (0%) | 0  (0%) | 0  (0%) | 0  (0%) | 0  (0%) | 0  (0%) | 0  (0%) | 0  (0%) |
|  | n(%) of positive & significant | 0  (0%) | 0  (0%) | 0  (0%) | 0  (0%) | 0  (0%) | 0  (0%) | 0  (0%) | 0  (0%) |
| **Competitive Sport** | n of intervention groups | 0 | 0 | 0 | 0 | 0 | 0 | 0 | 0 |
|  | n of outcomes | 0 | 0 | 0 | 0 | 0 | 0 | 0 | 0 |
|  | n(%) of positive | 0  (0%) | 0  (0%) | 0  (0%) | 0  (0%) | 0  (0%) | 0  (0%) | 0  (0%) | 0  (0%) |
|  | n(%) of positive & significant | 0  (0%) | 0  (0%) | 0  (0%) | 0  (0%) | 0  (0%) | 0  (0%) | 0  (0%) | 0  (0%) |
| PA: physical activity, n: number.  This table presents the effect direction and statistical significance for outcome domains across primary studies. All intervention arms from the same study were included as a unique intervention group. Only randomised clinical trials with sample size ≥ 50 participants per group and PEDro score ≥6 were included. | | | | | | | | | |

1. WHO. World report on ageing and health: World Health Organization (WHO), 2015. [↑](#footnote-ref-2)
2. WHO. International Classification of Functioning, Disability and Health (ICF): World Health Organization (WHO), 2018. [↑](#footnote-ref-3)
3. WHO. World report on ageing and health: World Health Organization (WHO), 2015. [↑](#footnote-ref-4)
4. WHO. World report on ageing and health: World Health Organization (WHO), 2015. [↑](#footnote-ref-5)
5. WHO. International Classification of Functioning, Disability and Health (ICF): World Health Organization (WHO), 2018. [↑](#footnote-ref-6)
