# Supplementary material for: Impact of physical activity programs and services for older adults: a rapid review
Source: Int J Behav Nutr Phys Act. 2022 Jul 14;19:87. doi: 10.1186/s12966-022-01318-9 (PMC9284866; doi:10.1186/s12966-022-01318-9)
Supplement: Supplementary file 3 — Additional file 3: [file 12966_2022_1318_MOESM3_ESM.docx]

**IJBN-D-22-00168 Impact of physical activity programs and services for older adults: a rapid review**

**Response to Reviewer Comments**

|  | **Reviewer comments** | **Author responses** | **Change/s made to manuscript** | **Location of change/s made** |
| --- | --- | --- | --- | --- |
| **Editor** | | | | |
| 1 | Please include the relevant populated checklist(s) with the submission of your revision. | We have now completed the PRISMA Scoping review checklist and uploaded this with our submission. | See attached PRISMA-Scr checklist | PRISMA-Scr checklist |
| 2 | Please add a statement on the role of the funding body under the relevant subheading of the Declarations section. | We have now added the role of the funding body. | *Added to Funding:*  Staff at the WHO (authors F.C.B. and J.W.) provided consultation on the scope, aims and methods of the review but were not involved in analysing data or interpreting results. | Lines 560-562 |
| **Reviewer 1** | | | | |
| 3 | P 5: It is unclear how the initial 39 + 342 reviews from the previous scoping review became 350 to be screened in this paper. Please explain. | There was overlap in the papers for the two research questions for the scoping reviews - 31 out of the 39 reviews included to answer question 1 were also included in question 2. We have now added this information to the manuscript to provide clarification to the reader. | *Added to Methods:*  Our previous scoping review identified 39 systematic reviews of physical activity interventions and 342 systematic reviews of physical activity programs/services for older adults.[6] Of the 39 reviews of interventions, 31 reviews were also included again as programs/services, resulting in 350 unique reviews. Therefore, for the current rapid review, we screened primary studies included in these 350 systematic reviews. | Lines 117-121 |
| 4 | P 9: The aim of the paper is to inform policy and thus reach a broad audience, who may not be familiar with all outcomes. I suggest that the concepts intrinsic capacity and functional ability are briefly explained in the methods section. In my opinion it is not enough to just refer to the appendix. | We have now added a definition of these concepts in the Methods section. | *Added to Methods*:  Intrinsic capacity refers to the composite of all the mental and physical capacities of an individual, while functional ability comprises the health-related attributes that enable people to be and to do what they have reason to value, encompassing both intrinsic capacity and environmental characteristics*.*[14] | Lines 213-216 |
| 5 | Table 1: It would be more logical with: mean (SD), median, range. | We have now made this change. | *Order of sample size data changed in Table 1:*  mean (SD), median, range  126 (295), 55, 6-5893 | Table 1 |
| 6 | P 11: …the actual number of participants included is smaller… How much smaller? Does this refer to the 26,861 on p 12? Could this be clarified? | We did not calculate the actual total number of participants in included studies for Objective 1- only the total number across all records. We have added this clarification in the text for the reader. The n=26,861 refers to the total number of participants included in studies for Objective 2.  We have now added ‘for Objective 2’ to clarify the sample size of 26,861 reported. | *Added to Results*:  The included records investigated a total of 178 622 participants, although the actual number of participants included in the studies is smaller as there are multiple publications from the same study, however we did not calculate the actual total number of participants included across individual studies in Objective 1.  The total number of participants across studies included in Objective 2 was 26,861 | Lines 260-261  Lines 288-289 |
| 7 | P 12: please add SD to the number of participants. | We have now added the SD for Objective 2. | *Added to Results:*  (mean 309, standard deviation 259, median 207, range 100 to 1,635). | Line 289 |
| 8 | It is helpful with the condensed presentation of the main results on pp 14-19. However, there are some inconsistences that are confusing: several numbers of intervention groups and some numbers of outcomes or % positive presented in the text are not the same as in the tables in the appendix. This involves tables A.9.1., A.9.2., A.9.3., A.9.4., and A.10.  For example on p 14: Across all studies combined, positive effects (>50% between-group comparisons positive) were evident for outcomes of physical activity (44 intervention groups, 125 individual outcomes), falls (38 intervention groups, 82 outcomes), intrinsic capacity: physical domain (56 intervention groups, 231 outcomes)… In table A.9.1.: physical activity (42 intervention groups, 122 individual outcomes), falls (36 intervention groups, 82 outcomes), intrinsic capacity: physical domain (52 intervention groups, 231 outcomes)… Please check if these numbers are correct or explain why the numbers differ. | Thank you for noticing this! We had updated the results after conducting a double-checking process, but we unfortunately uploaded the previous Appendix version to our submission. We have now uploaded the revised Appendix document and have checked to ensure consistency between tables, figures, and manuscript text. | Figures 3-6, Appendices 9-14 updated and/or checked |  |
| 9 | I think some of the >50% should be ≥50% - please check throughout the manuscript. | Yes you are correct - we have now amended this throughout. | ‘>50%’ changed to ‘≥50%’ throughout manuscript. | e.g., line 53 |
| 10 | P 19: The lack of information about the different measurement methods for physical activity in the studies are discussed, which is good. I suggest to also comment on how that could have affected the results. | We have now added further comment on this in the Limitations section. | *Added to Discussion:*  We also did not classify studies according to measurement tool, which could have affected overall results as different measurement tools have different properties such as the ability to detect change. | Lines 489-491 |
| 11 | P 20: In perspectives for future research, I think it also would be valuable with more studies on the oldest age group, 80+. | We have now added this to the Discussion. | *Added to Discussion:*  and v) the effectiveness of physical activity programs in the oldest age group ≥80 years (four out of 87 studies focused on participants aged ≥80 years). | Lines 471-472 |
| 12 | P 21: I think it is problematic that a different concept was used for multicomponent exercise compared to the WHO guidelines. Please explain why you decided to make this choice and discuss how the results in this manuscript are affected by this decision. How should the results from this study inform practice and policies in relation to the guidelines (and the ProFaNE taxonomy)? | Our definition of multicomponent exercise is consistent with the WHO guidelines (two or more types of exercise) but differs from the Sherrington et al Cochrane review. As this work was conducted to inform the WHO and others on the effectiveness of interventions, | *Added to Discussion:*  Hence, given that we classified a high number of interventions as ‘multicomponent’ in the current review, it is possible that some of these interventions could be re-classified as another type of structured exercise (e.g. strength/resistance/power or balance/functional/neuromotor) using the definition from the Cochrane review. However, our definition of multicomponent exercise is consistent with the WHO *Guidelines on Physical Activity and Sedentary Behaviour*[2] which emphasise incorporating strength and balance exercises for health benefits. | Lines 496-502 |
| **Reviewer 2** | | | | |
| 13 | p9, L210: although you describe concepts in Appendix 6, I feel that 'intrinsic capacity' needs spelling out here in the main text. It is not clear what it is. | We have now added this information to the Methods. | *Added to Methods:*  Intrinsic capacity refers to the composite of all the mental and physical capacities of an individual, while functional ability comprises the health-related attributes that enable people to be and to do what they have reason to value, encompassing both intrinsic capacity and environmental characteristics*.*[14] | Lines 213-216 |
| 14 | p19, L442: I can't believe that extraction of measurement tool details was not possible, given all of the other things extracted. This needs stating as a limitation of the study. | We have now added this as a limitation. | *Added to Discussion*:  We also did not classify studies according to measurement tool, which could have affected overall results as different measurement tools have different properties such as the ability to detect change. | Lines 489-491 |
| 15 | p21, L482: multi-component programs are mentioned in the 2020 WHO guidelines, so may be mention how they define it? | We have now added mention of the WHO guidelines in the Discussion. | *Added to Discussion:*  Hence, given that we classified a high number of interventions as ‘multicomponent’ in the current review, it is possible that some of these interventions could be re-classified as another type of structured exercise (e.g. strength/resistance/power or balance/functional/neuromotor) using the definition from the Cochrane review. However, our definition of multicomponent exercise is consistent with the WHO *Guidelines on Physical Activity and Sedentary Behaviour*[2] which emphasise incorporating strength and balance exercises for health benefits. | Lines 496-502 |
